# Reduced stress-associated FKBP5 DNA methylation together with gut microbiota dysbiosis is linked with the progression of obese PCOS patients

**DOI:** 10.1038/s41522-021-00231-6

**Published:** 2021-07-15

**Authors:** Fu Chen, Zhangran Chen, Minjie Chen, Guishan Chen, Qingxia Huang, Xiaoping Yang, Huihuang Yin, Lan Chen, Weichun Zhang, Hong Lin, Miaoqiong Ou, Luanhong Wang, Yongsong Chen, Chujia Lin, Wencan Xu, Guoshu Yin

**Affiliations:** 1 Department of Clinical Nutrition, The First Affiliated Hospital of Shantou University Medical College, Shantou, Guangdong Province China; 2 Institute for Microbial Ecology, School of Medicine, Xiamen University, Xiamen, Fujian Province China; 3 Department of Endocrinology, the First Affiliated Hospital of Shantou University Medical College, Shantou, Guangdong Province China; 4 Laboratory of Molecular Cardiology and Laboratory of Molecular Imaging, the First Affiliated Hospital of Shantou University Medical College, Shantou, Guangdong Province China; 5 Department of Reproductive Center, the First Affiliated Hospital of Shantou University Medical College, Shantou, Guangdong Province China; 6 Department of Gynecological tumor, Tumor Hospital Affiliated to Shantou University Medical College, Shantou, Guangdong Province China

**Keywords:** Clinical microbiology, Clinical microbiology

## Abstract

Polycystic ovary syndrome (PCOS) is a common endocrine disease in females that is characterized by hyperandrogenemia, chronic anovulation, and polycystic ovaries. However, the exact etiology and pathogenesis of PCOS are still unknown. The aim of this study was to clarify the bacterial, stress status, and metabolic differences in the gut microbiomes of healthy individuals and patients with high body mass index (BMI) PCOS (PCOS-HB) and normal BMI PCOS (PCOS-LB), respectively. Here, we compared the gut microbiota characteristics of PCOS-HB, PCOS-LB, and healthy controls by 16S rRNA gene sequencing, FK506-binding protein 5 (FKBP5) DNA methylation and plasma metabolite determination. Clinical parameter comparisons indicated that PCOS patients had higher concentrations of total testosterone, androstenedione, dehydroepiandrosterone sulfate, luteinizing hormone, and HOMA-IR while lower FKBP5 DNA methylation. Significant differences in bacterial diversity and community were observed between the PCOS and healthy groups but not between the PCOS-HB and PCOS-LB groups. Bacterial species number was negatively correlated with insulin concentrations (both under fasting status and 120 min after glucose load) and HOMA-IR but positively related to FKBP5 DNA methylation. Compared to the healthy group, both PCOS groups had significant changes in bacterial genera, including *Prevotella*_9, *Dorea*, *Maihella*, and *Slackia*, and plasma metabolites, including estrone sulfate, lysophosphatidyl choline 18:2, and phosphatidylcholine (22:6e/19:1). The correlation network revealed the complicated interaction of the clinical index, bacterial genus, stress indices, and metabolites. Our work links the stress responses and gut microbiota characteristics of PCOS disease, which might afford perspectives to understand the progression of PCOS.

## Introduction

Polycystic ovary syndrome (PCOS) is characterized by hyperandrogenemia, chronic anovulation, and polycystic ovaries and has a prevalence ranging from 6 to 20% among females of reproductive age; in addition, PCOS is the most common endocrine disorder and a cause of infertility in reproductive-aged women^1,2^. Many reports indicate that PCOS is linked to a higher risk of metabolic disorders, such as insulin resistance (IR), type 2 diabetes mellitus (T2DM), dyslipidemia, and cardiovascular diseases^3,4^. To date, the etiology of PCOS is unknown but multifactorial elements, including inherent genetics, intrauterine environment, lifestyle, and potential alteration in the gut microbiota, are thought to be involved in its development^5^.

Accumulating evidence indicates that gut inhabitants play a significant role in the development of obesity, obesity-associated inflammation, and insulin resistance^6–8^. For instance, Zhao et al.^9^ revealed that short-chain fatty acid (SCFA) producers may be beneficial in the improvement of hemoglobin A1c levels in T2DM partly by increasing glucagon-like peptide-1(GLP-1) production. The glutamate-fermenting commensal, *Bacteroides thetaiotaomicron*, reduced plasma glutamate concentration and alleviated diet-induced body weight gain and adiposity in mice, and its abundance was related to bariatric surgery efficiency^10^. A recent randomized controlled trial showed that fecal microbiota transplantation (FMT) halted the decline in endogenous insulin production in recently diagnosed patients with T1DM in 12 months^11^. PCOS has obvious heterogeneity in which a large proportion of patients have IR and metabolic abnormalities; however, there are still some patients with normal body mass index (BMI) and metabolic normalities. In addition to race, lifestyle, and diet habits, different subtypes caused by different etiologies may be an important reason for the difference in gut microbiota. Limited research has shown that PCOS patients have decreased alpha diversity^12–14^ and featured bacteria from Bacteroidaceae, Clostridiaceae, Erysipelotrichidae, Lachnospiraceae, Lactobacillaceae, Porphyromonadaceae, Prevotellaceae, Ruminococcaceae^15^, and Actinobacteria^5,12,16,17^. Qi et al.^14^ revealed that *Bacteroides vulgatus* was markedly elevated in the gut microbiota of PCOS individuals, accompanied by reduced glycodeoxycholic acid and tauroursodeoxycholic acid levels. Hyperandrogenism is associated with gut microbial dysbiosis, indicating that androgens may modulate the gut microbial community and that modulation of the gut microbiota may be a potential treatment target for PCOS^18^. For example, FMT from PCOS women or exposure to certain bacteria resulted in a PCOS-like phenotype in mice, while exposure to a healthy gut microbiome resulted in protection from developing PCOS-like traits in mice^19^. The PCOS-associated lipidomic analysis revealed that PCOS patients had distinguished lipid characteristics that made potential lipid biomarkers for PCOS diagnosis possible^20,21^. For example, Jiang et al.^22^ revealed that phosphatidylcholine (PC) was higher, whereas lysophosphatidylcholine was lower in PCOS women than in healthy controls. In addition, metabolomics analysis between the healthy and PCOS groups also revealed metabolic disorders associated with lipid and amino acid metabolism^23^.

Depression and anxiety are more common in patients with PCOS^24,25^, and these unhealthy psychological conditions may play a role in the development of PCOS. The gut microbiota composition has changed as a response to stressful conditions^26,27^. The brain-gut-microbiota axis plays a role in the pathogenesis of stress-related psychiatric disorders^28,29^. FK506-binding protein 5 (FKBP5) is an important modulator of the stress response, and Zannas et al. suggested that methylation measurement of FKBP5 CpGs may be associated with stress-related disease^8^. Interestingly, with respect to hormone-binding function, FKBP5 serves to increase androgen receptor (AR) function, suggesting that FKBP5 can directly or indirectly target the ligand-binding domains of AR^30^. However, the methylation level of FKBP5 CpGs in PCOS patients and its relationship with gut microbiota have not been reported.

To elucidate the relationship among gut microbiota, metabolites, and PCOS clinical features, we characterized gut microbiota from a large number of PCOS patients with high BMI (BMI ≥ 24) and normal BMI (BMI < 24) using 16S rRNA gene sequencing and untargeted metabolomics, and their association with FKBP5 CpG methylation was also investigated. Our findings can help to illuminate the microbiome-based process underpinned in PCOS disease and in the development of advanced approaches to develop biomarkers for diagnose of PCOS.

## Results

### Clinical characteristics of the patients with PCOS and healthy individuals

**Fig. 1:**
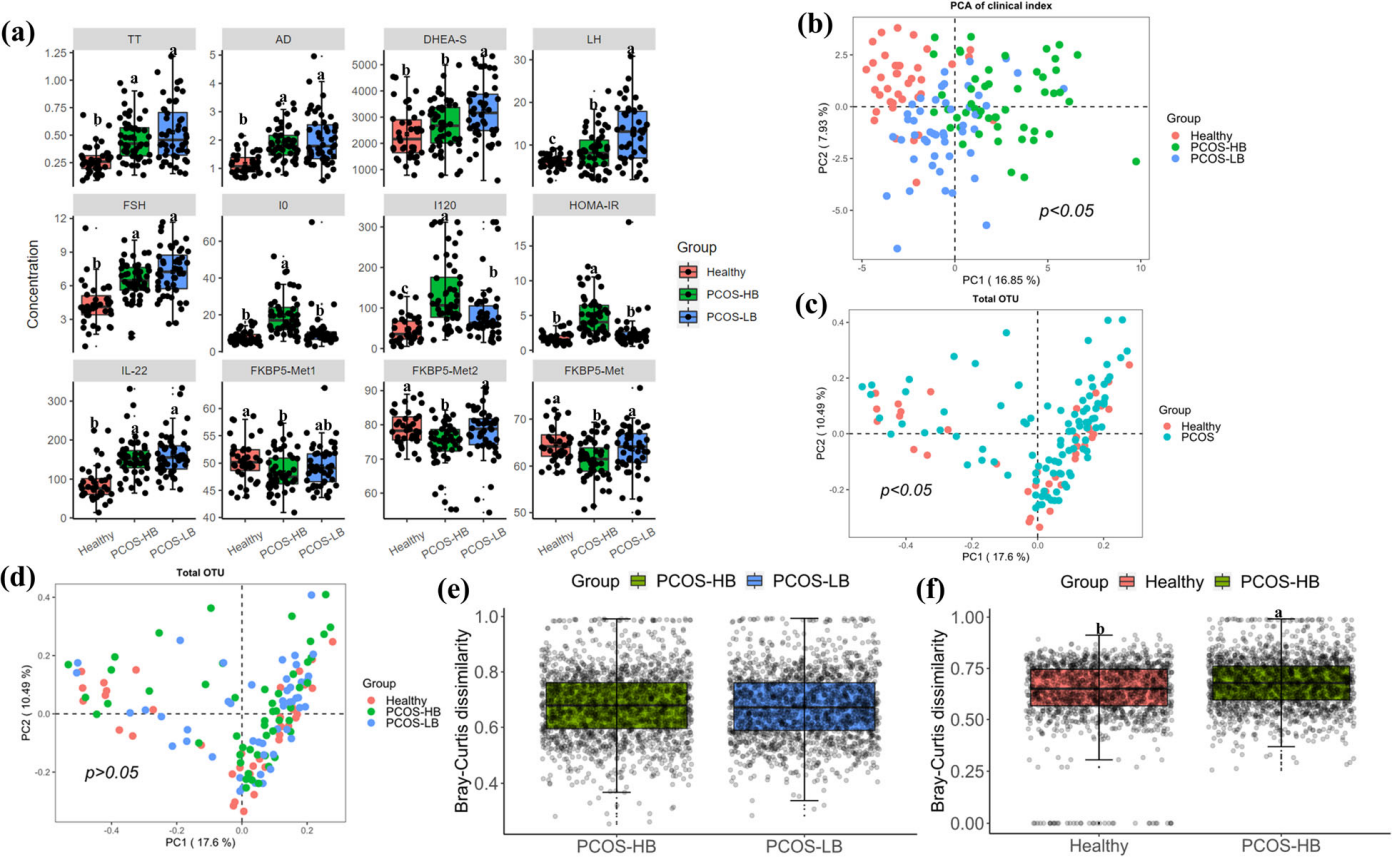
Clinical and bacterial community characteristics comparison. **a** Differences in clinical index among healthy participants, and participants with PCOS-HB and PCOS-LB, respectively. The data are shown as the mean ± SD and error bar was used. **b** Differences in clinical index structures among healthy participants, and participants with PCOS-HB and PCOS-LB, respectively. **c** Differences in clinical index structures between healthy participants and participants with PCOS. **d** Differences in bacterial structures among healthy participants, and participants with PCOS-HB and PCOS-LB, respectively. **e** Comparison of Bray–Curtis distance between the PCOS-HB and PCOS-LB groups relative to the healthy group. **f** Comparison of Bray–Curtis distance between the PCOS-HB and healthy relative to the PCOS-LB group. The data are shown as the mean ± SD and error bar was used. Red, green, and blue color represent Healthy, PCOS-HB and PCOS-LB group separately. PCOS-LB, normal BMI (BMI < 24); PCOS-HB, high BMI (BMI ≥ 24).

The participant demographics are shown in Table 1. Among all of the groups, there were no significant differences in age, aspartate aminotransferase (AST), albumin (ALB), globulin (GLB), ALB/GLB ratio, total bilirubin (TBIL), direct bilirubin(DBIL), or indirect bilirubin (IBIL) (*p* > 0.05), while glucose level at fasting status (G0) and glucose level at 120 min after glucose load (G120), insulin level at fasting status (I0) and insulin level at 120 min after glucose load (I120), FKBP5-Met1, FKBP5-Met2, and FKBP5-Met (The methylation results of CpG sites at Chr6: 35657180 and Chr6:35657202 were expressed as FKBP5-Met1 and FKBP5-Met2 respectively. FKBP5-Met was the average level of FKBP5-Met1 and FKBP5-Met2.) differed significantly (*p* < 0.05). Within PCOS patients, the PCOS-HB group was featured as higher I0 and I120 while lower dehydroepiandrosterone (DHEA), dehydroepiandrosterone sulfate (DHEA-S), FKBP5-Met, FKBP5-Met2, and luteinizing hormone (LH) than the PCOS-LB group. PCOS-LB patients and healthy individuals had similar levels of DHEA, DNA methylation, and LH/follicle-stimulating hormone(FSH) ratios (Fig. 1a). The differences between the clinical parameters are displayed via PCoA ordination (Fig. 1b), which shows significant differences among the healthy, PCOS-HB and PCOS-LB subjects (Df = 2, *F* = 10.45, *R*^2^ = 0.14, *p* < 0.05).

**Table 1:** Clinical characteristics baseline in the healthy and PCOS group.

Parameter	Characteristic	Healthy (*n* = 38)	PCOS-LB (*n* = 48)	PCOS-HB (*n* = 50)	Significance	FDR
Demographic characteristics	Age (years)	29.26 ± 4.18	29.48 ± 3.39	29.64 ± 4.06	*p* > 0.05	0.920212
	BMI (kg/m^2^)	20.19 ± 1.6b	20.57 ± 2.12b	28.18 ± 2.82a	***p*** < **0.001**	2.93E-37
Liver function	LHD (U/L)	176.84 ± 35.27ab	169.73 ± 34.14b	191.88 ± 34.45a	***p*** < **0.01**	0.010185
	AST (U/L)	22.11 ± 12.2	21.67 ± 7.11	24.3 ± 11.42	*p* > 0.05	0.451829
	ALT (U/L)	15.42 ± 11.65b	20.81 ± 13.21b	30.66 ± 24.97a	***p*** < **0.001**	0.001024
	GGT (U/L)	18.08 ± 11.03b	22.15 ± 12.44b	31.22 ± 19.06a	***p*** < **0.001**	0.000456
	ALP (U/L)	63.76 ± 16.06b	70.69 ± 18.44ab	79.02 ± 23.78a	***p*** < **0.01**	0.003977
	CHE (U/ml)	7.35 ± 1.64b	7.91 ± 1.35b	9.42 ± 1.44a	***p*** < **0.001**	9.11E-09
	MAO (U/L)	3.58 ± 1.37ab	3.04 ± 1.03b	4.06 ± 1.45a	***p*** < **0.001**	0.001547
	AFU (U/L)	24.61 ± 6.91b	26.56 ± 6.15ab	29.24 ± 7.08a	***p*** < **0.01**	0.009844
	TP (g/L)	75.85 ± 4.07b	78.04 ± 3.6a	76.77 ± 4.35ab	***p*** < **0.05**	0.061122
	ALB (g/L)	44.08 ± 2.09	44.91 ± 2.34	44.13 ± 2.49	*p* > 0.05	0.1879
	GLB (g/L)	31.78 ± 2.8	33.13 ± 2.97	32.65 ± 3.22	*p* > 0.05	0.158354
	ALB/GLB	1.39 ± 0.12	1.37 ± 0.14	1.36 ± 0.15	*p* > 0.05	0.451829
	TBIL (µmol/L)	11.06 ± 4.13	11.94 ± 4.77	11.14 ± 3.1	*p* > 0.05	0.534788
	DBIL (µmol/L)	2.07 ± 0.81	2.13 ± 0.85	1.92 ± 0.65	*p* > 0.05	0.408893
	IBIL (µmol/L)	9.01 ± 3.39	9.81 ± 3.99	9.22 ± 2.54	*p* > 0.05	0.534788
Renal function	BUN (mmol/L)	4.67 ± 1.14a	4.06 ± 0.95b	4.35 ± 1.09ab	***p*** < **0.05**	0.046988
	Cr (µmol/L)	74.84 ± 7.31	70.94 ± 7.08	72.62 ± 7.95	*p* > 0.05	0.081796
	CO_2_ (mmol/L)	25.39 ± 2.57	26.29 ± 1.97	25.96 ± 2.37	*p* > 0.05	0.223886
Metabolic index	UA (µmol/L)	331.65 ± 85.39b	337.71 ± 65.99b	410.35 ± 92.22a	***p*** < **0.001**	1.5E-05
	TC (mmol/L)	4.59 ± 0.7	4.87 ± 0.66	4.95 ± 0.82	*p* > 0.05	0.08753
	TG (mmol/L)	0.93 ± 0.32b	1.04 ± 0.46b	1.48 ± 0.81a	***p*** < **0.001**	7.36E-05
	HDL-C (mmol/L)	1.52 ± 0.21a	1.49 ± 0.3a	1.2 ± 0.21b	***p*** < **0.001**	9.11E-09
	LDL -C(mmol/L)	2.79 ± 0.55b	3.05 ± 0.45a	3.29 ± 0.61a	***p*** < **0.001**	0.000493
Thyroid function	FT3 (pmol/L)	5.04 ± 0.82b	5.25 ± 0.87b	5.61 ± 0.55a	***p*** < **0.01**	0.003062
	FT4 (pmol/L)	10.99 ± 1.72	10.87 ± 1.17	10.93 ± 1.25	*p* > 0.05	0.920212
	TSH (mIU/L)	2.06 ± 1a	1.49 ± 0.61b	2.47 ± 1.8a	***p*** < **0.001**	0.002038
Sex hormone	LH (mIU/ml)	6.09 ± 1.56c	12.67 ± 6.8a	8.54 ± 4.68b	***p*** < **0.001**	1.89E-07
	FSH (mIU/ml)	4.31 ± 1.84b	7.24 ± 2.15a	6.45 ± 1.67a	***p*** < **0.001**	2.19E-09
	LH/FSH	1.92 ± 2.39	1.82 ± 0.95	1.37 ± 0.8	*p* > 0.05	0.1879
	PRL (ng/ml)	25.57 ± 15.59a	15.49 ± 7.65b	15.14 ± 7.1b	***p*** < **0.001**	1.5E-05
	E2 (pg/ml)	28.32 ± 15.03c	68.68 ± 39.85a	51.41 ± 26.14b	***p*** < **0.001**	1.49E-07
	PROG (nmol/L)	0.87 ± 0.35a	0.75 ± 0.47ab	0.58 ± 0.43b	***p*** < **0.01**	0.009844
Androgen	AD (ng/ml)	1.15 ± 0.37b	2.03 ± 0.89a	1.87 ± 0.6a	***p*** < **0.001**	1.49E-07
	TT(ng/ml)	0.28 ± 0.13b	0.53 ± 0.26a	0.47 ± 0.2a	***p*** < **0.001**	1.33E-06
	DHEA (ng/ml)	10.07 ± 5.04	11.72 ± 7.66	9.3 ± 4.86	*p* > 0.05	0.170052
	DHEA-S (ng/ml)	2348.03 ± 957.26b	3233.54 ± 1121.45a	2684.35 ± 953.11b	***p*** < **0.001**	0.000817
	FAI	1.56 ± 0.87c	4.4 ± 2.64b	9.04 ± 5.41a	***p*** < **0.001**	5.29E-15
	SHBG (nmol/L)	68.55 ± 26.31a	48.64 ± 23.09b	21.63 ± 10.46c	***p*** < **0.001**	1.84E-17
Glucose tolerance	G0 (mmol/L)	4.81 ± 0.41b	5.31 ± 0.81a	5.56 ± 1.03a	***p*** < **0.001**	0.000409
	G120 (mmol/L)	5.69 ± 1.6c	6.98 ± 1.91b	8.18 ± 3.28a	***p*** < **0.001**	8.54E-05
Insulin	I0 (mIU/L)	7.78 ± 3.3b	10.52 ± 9.89b	19.67 ± 10.71a	***p*** < **0.001**	2.61E-08
	I120 (mIU/L)	46.88 ± 33.87c	86.5 ± 68.75b	133.89 ± 82.69a	***p*** < **0.001**	3.22E-07
	HOMA-IR	1.69 ± 0.8b	2.52 ± 2.59b	4.89 ± 2.75a	***p*** < **0.001**	1.02E-08
Inflammatory factor	IL-22 (pg/ml)	88.77 ± 46.24b	161.71 ± 52.87a	154.61 ± 50.27a	***p*** < **0.001**	2.19E-09
	IL-8 (pg/ml)	4.09 ± 6.55	2.21 ± 2.43	2.52 ± 2.23	*p* > 0.05	0.100205
Methylation	FKBP5-Met1	50.43 ± 3.75a	49.39 ± 3.78ab	48.29 ± 3.5b	***p*** < **0.05**	0.041665
	FKBP5-Met2	78.98 ± 4.78a	77.76 ± 7.1a	74.47 ± 6.9b	***p*** < **0.001**	0.005897
	FKBP5-Met	64.7 ± 3.9a	63.57 ± 4.84a	61.38 ± 4.17b	***p*** < **0.001**	0.003062

The data are shown as the mean ± SD. *n* = 38 in the control group, *n* = 48 in the PCOS-LB, and *n* = 50 in the PCOS-HB group for all outcomes. Letters indicate the ANOVA grouping among groups.

*BMI* body mass index, *LDH* lactate dehydrogenase, *AST* aspartate aminotransferase, *ALT* alanine aminotransferase, *GGT* glutamyltransferase, *ALP* alkaline phosphatase, *CHE* cholinesterase, *MAO* monoamine oxidase, *AFU* α-l-fucosidase, *TP* total protein, *ALB* albumin, *GLB* globulin, *TBIL* total bilirubin, *DBIL* direct bilirubin, *IBIL* indirect bilirubin, *BUN* urea nitrogen, *Cr* creatinine, *UA* uric acid, *TG* triglycerides, *TC* total cholesterol, *HDL-C* high-density lipoprotein cholesterol, *LDL-C* low-density lipoprotein cholesterol, *FT3* free triiodothyronine, *FT4* free thyroxine, *TSH* thyroid-stimulating hormone, *LH* luteinizing hormone, *FSH* follicle-stimulating hormone, *PRL* prolactin, *E2* estrogen, *PROG* progesterone, *AD* androstenedione, *TT* total testosterone, *DHEA* dehydroepiandrosterone, *DHEA-S* dehydroepiandrosterone sulfate, *FAI* free androgen index, *SHBG* sex hormone-binding globulin, *G0* glucose level at fasting status, *G120* glucose level at 120 min after glucose load, *I0* insulin level at fasting status, *I120* insulin level at 120 min after glucose load, *HOMA-IR* homeostasis model assessment for IR, *IL-22* interleukin-22, *IL-8* interleukin-8, *FKBP5-Met1* FKBP5 DNA methylation at CpG 35657180/hg19, *FKBP5-Met2* FKBP5 DNA methylation at CpG 35657202/hg19, *FKBP5-Met* average of FKBP5 DNA methylation at CpG 35657180 and 35657202/hg19.

### Comparison of alpha- and beta-diversities in the gut microbiome among the three participant groups

An average of 69,379 raw reads, 68,424 clean reads, 59,441 OTU sequences/samples, and 227 OTUs were obtained from 136 samples (Supplementary Table 2). Permutational multivariate analysis of variance (PERMANOVA) (Df = 1, *F* = 1.73, *R*^2^ = 0.01, *p* < 0.05) showed significant differences in the overall bacterial community between the PCOS and healthy groups (Fig. 1c). However, there were no significant differences in beta diversity among the healthy, PCOS-HB and PCOS-LB groups (PERMANOVA, Df = 2, *F* = 1.21, *R*^2^ = 0.02, *p* > 0.05) (Fig. 1d), and there was no significant difference (Df = 1, *F* = 0.63, *p* > 0.05) in Bray–Curtis distance between the PCOS-HB and PCOS-LB groups relative to the healthy group (Fig. 1e), indicating that the bacterial community was homogenized within PCOS patients. However, the bacterial community difference between healthy individuals and PCOS-LB is significantly smaller (Df = 1, *F* = 78.48, *p* < 0.05) than that of the difference between PCOS-HB and PCOS-LB (Fig. 1f).

**Fig. 2:**
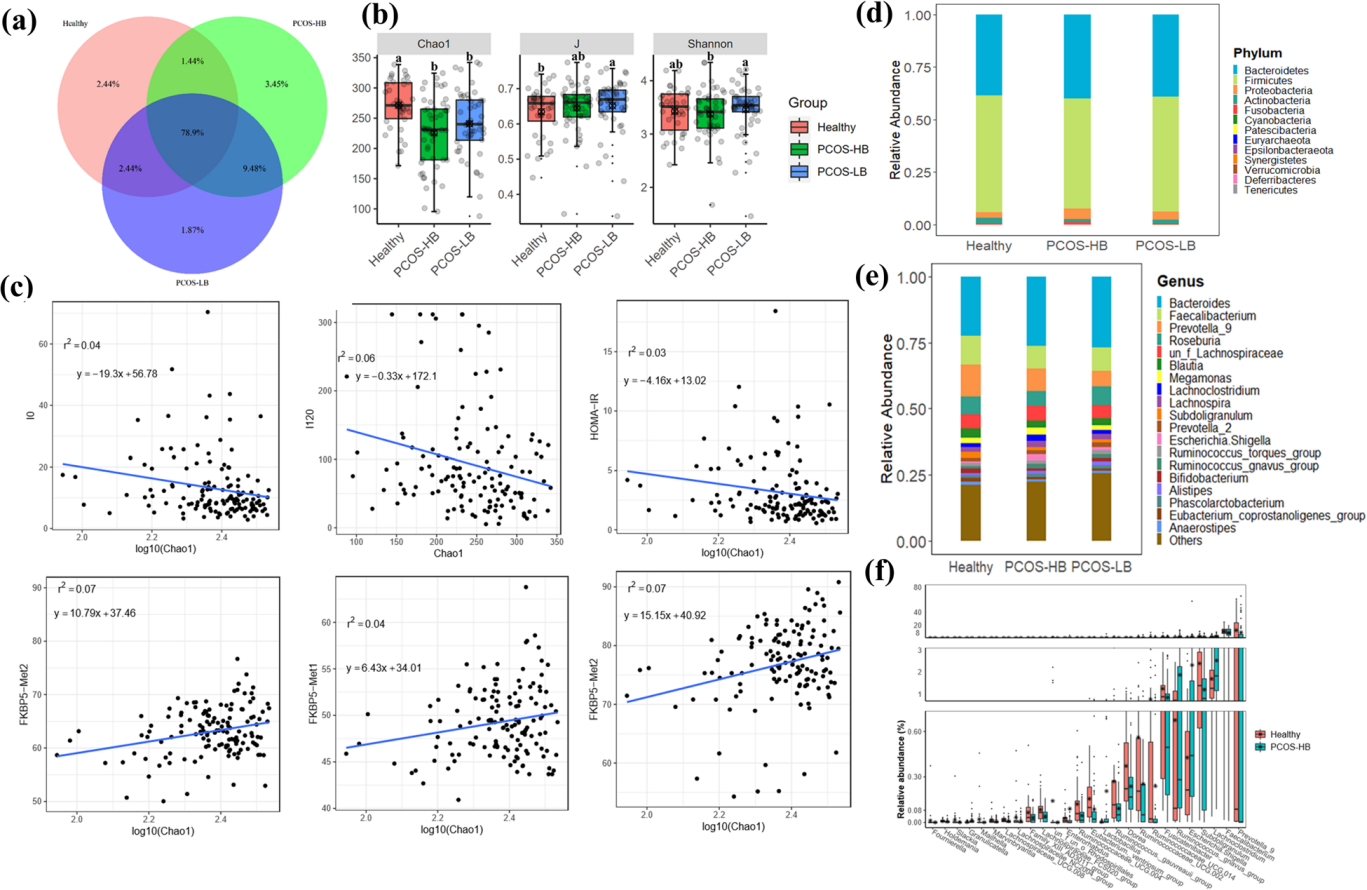
Changes in bacterial diversity and community composition. **a** Venn diagram showing the shared and unique OTUs among healthy, PCOS-HB and PCOS-LB subjects. **b** The bacterial diversity comparison among healthy subjects, and subjects with PCOS-HB and PCOS-LB, respectively. The data are shown as the mean ± SD and error bar was used. **c** The association between bacterial diversity and clinical indices. **d** Distribution of bacterial taxa at the phylum level. **e** Distribution of bacterial taxa at the genus level. **f** The distinguished bacterial genera screened by the Wilcoxon test. Letters indicate the ANOVA grouping. Red, green, and blue color represent Healthy, PCOS-HB and PCOS-LB group separately. PCOS-LB, normal BMI (BMI < 24); PCOS-HB, high BMI (BMI ≥ 24).

Shared “universal” OTUs (found in samples from all PCOS and healthy individuals) accounted for 78.9% of the total OTUs. Individuals in the PCOS-HB group had more exclusive OTUs (3.45%) than patients who were in the PCOS-LB group (1.87%) and individuals in the healthy group of (2.44%) (Fig. 2a). Compared with healthy participants, patients with PCOS-LB displayed a lower Chao1 index but higher J indices. No significant difference (*p* > 0.05) was observed between PCOS-HB and PCOS-LB groups for either Chao1 or J indices, while reduced Shannon indices were observed in the PCOS-HB group relative to the PCOS-LB group (Supplementary Table 3 and Fig. 2b). Further correlation between the bacterial diversity indices and clinical parameters showed that observed species, Chao1 and ACE values were significantly negatively related to I0 and I120 but were significantly positively related to FKBP5-Met1 and FKBP5-Met2 (*p* < 0.05) (Supplementary Table 4). The synergistic negatively linear relationship between Chao1 and I0 (DF = 134, *F* value = 5.83, *p* = 0.017, adjusted *R*^2^ = 0.03), Chao1 and I120 (DF = 134, *F* value = 8.16, *p* = 0.005, adjusted *R*^2^ = 0.05), Chao1 and HOMA-IR (DF = 134, *F* value=4.36, *p* = 0.04, adjusted *R*^2^ = 0.02) while positively linear relationship between Chao1 and FKBP5-Met1 (DF = 134, *F* value=5.33, *p* = 0.02, adjusted *R*^2^ = 0.03), Chao1 and FKBP5-Met2 (DF = 134, *F* value = 9.54, *p* = 0.002, adjusted *R*^2^ = 0.06), Chao1 and FKBP5-Met (DF = 134, *F* value=10.62, *p* = 0.001, adjusted *R*^2^ = 0.07) were depicted in Fig. 2c.

### Gut microbial community shift in patients with PCOS

Thirteen bacterial phyla were detected, and Firmicutes (healthy, 55.41%; PCOS, 53.47%), Bacteroidetes (healthy, 38.63%; PCOS, 39.57%), Proteobacteria (healthy, 2.82%; PCOS, 4.45%), and Actinobacteria (healthy, 2.50%; PCOS, 1.51%) were the dominant taxa (occupying ~99.0%) (Supplementary Table 5 and Supplementary Figure 1a). Firmicutes and Actinobacteria were abundant in the healthy group, while Bacteroidetes and Proteobacteria were lower in the PCOS group. PCOS-HB group was featured as higher abundance of Proteobacteria and Fusobacteria (Fig. 2d). The abundant bacterial genus (relative abundance > 1%) added up to 76.89%; therein, the average abundance of *Bacteroides* (25.49%), *Faecalibacterium* (9.34%), *Prevotella*_9 (8.63%), *Roseburia* (6.23%), un_f_Lachnospiraceae (5.41%), *Blautia* (2.86%), and *Megamonas* (2.06%) reached >2% (Supplementary Table 6). Healthy individuals were featured as higher *Faecalibacterium* and *Prevotella*_9 while lower *Bacteroides*, and the PCOS-HB group had a higher abundance of *Bacteroides* and *Megamonas* than the healthy group (Fig. 2e).

To screen out the differentiated microbiota taxa, the Wilcox test between groups was conducted. The comparison between the PCOS and healthy groups identified a total of 29 distinct bacterial genera. Compared with the healthy group, the highly increased taxon in the PCOS group was *Escherichia. Shigella* (*p* < 0.05, FC = 4.12), *Gemalla* (*p* < 0.05, FC = 3.80), *Granulicatella* (*p* < 0.05, FC = 4.26), *Prevotella*_2 (*p* < 0.05, FC = 1.18), *Romboutsia* (*p* < 0.05, FC = 7.01), and *Ruminococcus gnavus* groups (*p* < 0.05, FC = 2.42), while the significantly downward taxa consisted of *Alloprevotella*, *Coprobacillus*, *Lactococcus*, *Maihella*, *Ruminococcus*_1, *Ruminococcus gauvreauii*, and *Slackia* (Table 2, Supplementary Fig. 2, and Supplementary Table 7). There were 26 differential genera in the comparison between healthy and PCOS-HB groups and *Prevotella_9*, *Faecalibacterium*, *Lachnoclostridium*, *Subdoligranulum*, and *Escherichia. Shigella* ranked as the fifth most-abundant genera (Fig. 2f). *Prevotella_9*, *Megamonas*, *Alistipes*, *Romboutsia*, *Dorea,* and *Alloprevotella* were the most abundant taxa, which differentiated the healthy and PCOS-LB groups (Supplementary Fig. 1b), and among the selected distinguishing taxa, *Lactococcus*, *Romboutsia*, and *Slackia* were increased in the PCOS-LB group, while a low amount of *Mailhella* only existed in the group of healthy individuals. Further comparisons between PCOS-HB and PCOS-LB were made, and 18 distinguished genus taxa were screened out (Supplementary Fig. 1c). For example, the relative abundances of Erysipelotrichaceae_UCG.003, *Holdemania*, *Sellimonas*, *Terrisporobacter*, *Turicibacter*, and un_f_Flavobacteriaceae in PCOS-LB were twice higher than those in PCOS-HB (Supplementary Table 8).

**Table 2:** Bacterial genus comparison between healthy and PCOS patients.

Genus	Healthy (RA)	PCOS (RA)	*p* value	Fold change (PCOS/healthy)	Trend
*Alloprevotella*	0.0049956	0.00047869	0.02042	0.095821759	Decrease
*Coprobacillus*	0.000283648	1.1821E-05	0.0395	0.041675466	Decrease
*Dorea*	0.003719603	0.00244005	0.00532	0.655998003	Decrease
*Escherichia.Shigella*	0.0042742	0.01762568	0.0151	4.123738398	Increase
*Eubacterium coprostanoligenes_*group	0.014135001	0.00960852	0.0497	0.679767712	Decrease
*Eubacterium ventriosum*_group	0.001557949	0.0012568	0.01153	0.806700495	Decrease
*Faecalibacterium*	0.110499221	0.08677147	0.04239	0.785267664	Decrease
*Fusicatenibacter*	0.012491533	0.00898242	0.03508	0.719080499	Decrease
*Gemella*	7.62632E-06	2.8898E-05	0.02924	3.789242405	Increase
*Granulicatella*	2.2023E-05	9.391E-05	0.001	4.264160754	Increase
gut_metagenomeun_o_Rhodospirillales	9.48317E-05	0	0.02334	0	Decrease
Lachnospiraceae_FCS020_group	0.000837398	0.00046588	0.00562	0.556346574	Decrease
Lachnospiraceae_UCG.008	0.000180355	0.00011	0.00574	0.609889874	Decrease
*Lactococcus*	0.00032091	4.7608E-05	0.02511	0.148353161	Decrease
*Mailhella*	0.00021083	2.6265E-06	0.00197	0.012458022	Decrease
*Marvinbryantia*	0.000157499	9.2262E-05	0.01597	0.585796988	Decrease
*Prevotella*_2	0.013640525	0.01611411	0.04456	1.181341114	Increase
*Prevotella*_9	0.120546303	0.07298184	0.00019	0.605425791	Decrease
Prevotellaceae_NK3B31_group	0.000916144	0.00020389	0.02405	0.22254725	Decrease
*Romboutsia*	0.001324257	0.00928611	0.03992	7.012315071	Increase
Ruminococcaceae_UCG.002	0.005613696	0.0031676	0.03004	0.564263034	Decrease
Ruminococcaceae_UCG.004	0.001207413	0.00056306	0.00694	0.466339893	Decrease
Ruminococcaceae_UCG.014	0.007713543	0.00424645	0.04458	0.550518813	Decrease
*Ruminococcus*_1	0.011817551	0.00542379	0.03948	0.45896079	Decrease
*Ruminococcus gauvreauii*_group	0.002712018	0.00098627	0.00714	0.363664921	Decrease
*Ruminococcus gnavus*_group	0.006745751	0.01636757	0.02766	2.426352615	Increase
*Slackia*	0.000150718	1.6417E-05	0.00025	0.108922665	Decrease
*Subdoligranulum*	0.023987333	0.0127354	0.01066	0.530922024	Decrease
un_f_un_o_Rhodospirillales	0.001419089	0	0.00028	0	Decrease

*RA* relative abundance.

### Functional profiling of the gut microbiota by PICRUSt analysis

To predict bacterial functions coded by the gut microbiome, PICRUSt analysis was performed to compare the difference between the PCOS and healthy groups. The mean nearest sequenced taxon index (NSTI) value was 0.078 ± 0.025 for all samples (Supplementary Table 9). The Wilcox test was performed to compare the significantly different functions in KEGG level 3, and those featuring |logFC | >1 (the abundance in one group was twice more than that in the other group) were determined. Compared with PCOS patients, the p53 signaling and cardiac muscle contraction pathways were more highly expressed in the healthy group (Supplementary Fig. 3a), the two of which were also distinguished pathways between healthy and PCOS-LB patients (Supplementary Fig. 3b). However, cardiac muscle contraction was the only function that occurred between the healthy and PCOS-HB groups. The relative abundance of alpha-linolenic acid metabolism in PCOS-HB patients was twice more than that in PCOS-LB patients (Supplementary Fig. 3c).

### Metabolite profile related with PCOS disease

**Fig. 3:**
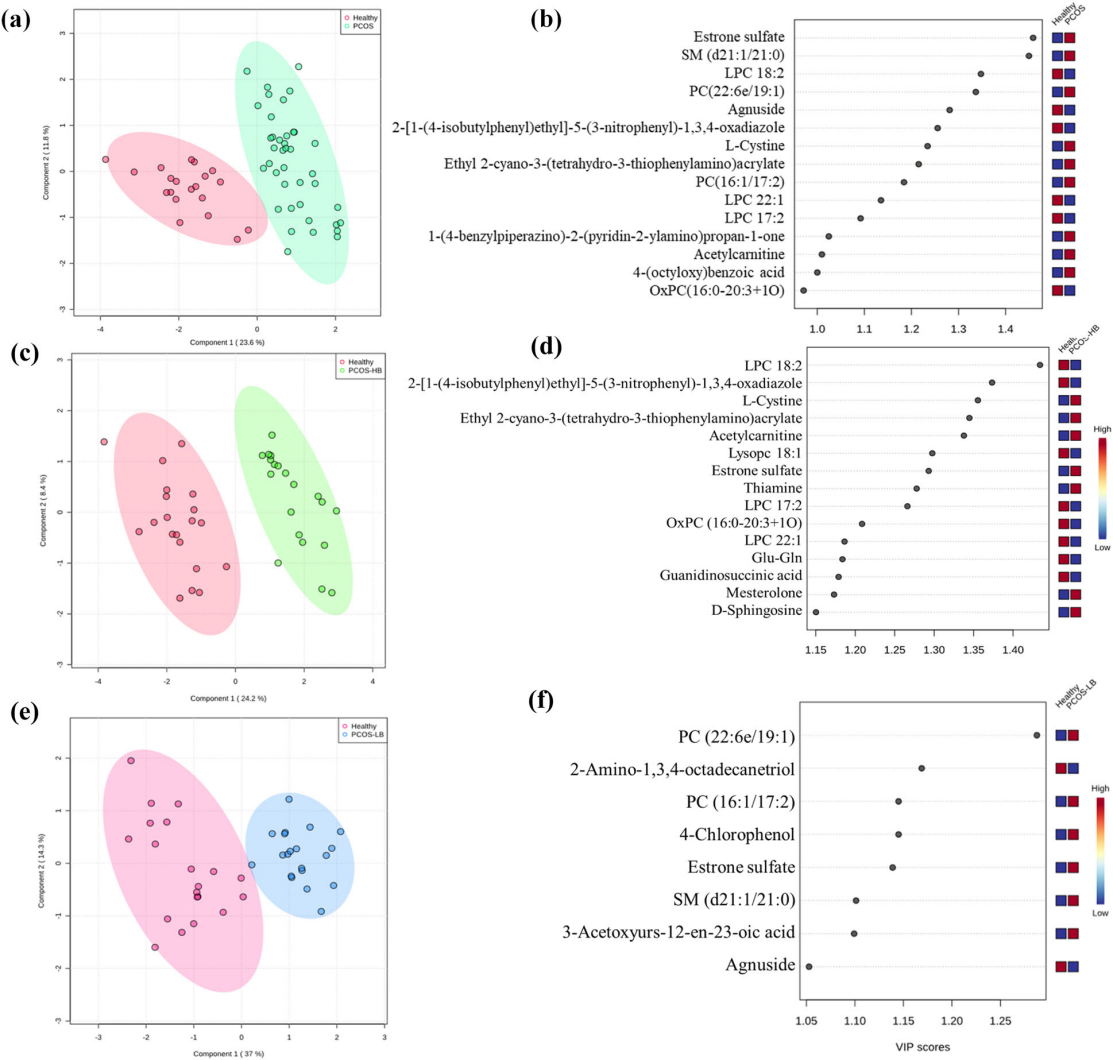
Comparison of pairwise metabolite profile characteristics. **a** The PLS-DA plot showing the distribution pattern difference between healthy patients and patients with PCOS. **b** VIP scores of PLS-DA in comparison between healthy patients and patients with PCOS. **c** The PLS-DA plot showing the distribution pattern difference between healthy patients and patients with PCOS-HB. **d** VIP scores of PLS-DA in comparison between healthy patients and patients with PCOS-HB. **e** The PLS-DA plot showing the distribution pattern difference between healthy patients and patients with PCOS-LB. **f** VIP scores of PLS-DA in comparison between healthy patients and patients with PCOS-LB. VIP scores were used to rank the discriminating power of different taxa between the PCOS and control groups. A taxon with a VIP score of >1 was considered important in the discrimination. Red, green, and blue color represent Healthy, PCOS-HB and PCOS-LB group separately.

**Fig. 4:**
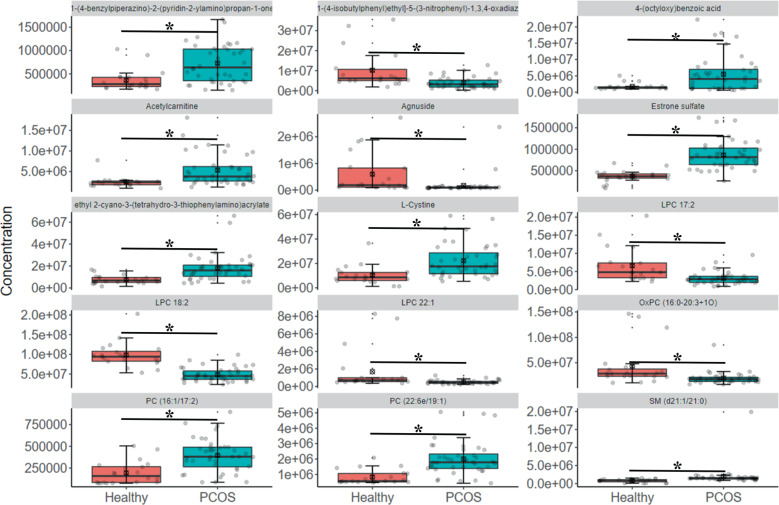
The distribution of distinguished metabolites in PCOS patients. The metabolites were screened out based on metabolites with VIP > 1, *p* < 0.05, and FC ≥ 2 or FC ≤ 0.5. Red and green color represent Healthy and PCOS group separately. *p < 0.05 denotes significant difference. The data are shown as the mean ± SD and error bar was used.

To assess whether the profiles of plasma metabolites were associated with PCOS, 20 healthy individuals, 20 PCOS-HB and 20 PCOS-LB patients were included for the untargeted metabolome analysis. Significant differences in the composition of plasma metabolites were observed between the healthy group and the PCOS group (Fig. 3a, b, Fig. 4, and Supplementary Fig. 4a). With the screening criteria of *p* < 0.05, fold change > 2 or fold change < 0.5 and VIP > 1, we observed the healthy group-enriched metabolites were 2-[1-(4-isobutylphenyl)ethyl]-5-(3-nitrophenyl)-1,3,4-oxadiazole, agnuside, lysophosphatidyl choline (LPC) 17:2, LPC 18:2, LPC 22:1, OxPC (16:0-20:3 + 1O) while 1-(4-benzylpiperazino)-2-(pyridin-2-ylamino) propan-1-one, 4-(octyloxy)benzoic acid, acetylcarnitine, estrone sulfate, l-Cystine, PC (16:1/17:2). PC (22:6e/19:1) and ethyl 2-cyano-3-(tetrahydro-3-thiophenylamino) acrylate and sphingosine (SM) (d21:1/21:0) were higher in PCOS patients. Estrone sulfate, SM (d21:1/21:0) and LPC 18:2 ranked ahead in the difference comparison between the two groups. With regard to the comparison between healthy and PCOS-HB patients, the lipids LPC 18:2, LPC 17:2, and LPC 22:1 had different abundances (Fig. 3c, d, Supplementary Fig. 4b, and Supplementary Table 10). Although they had similar BMI, the healthy group and the PCOS-LB group had distinguished metabolite profiles mainly in PC (22:6e/19:1), 2-Amino-1,3,4-octadecanetriol, PC (16:1/17:2), 4-Chlorophenol, estrone sulfate, SM (d21:1/21:0), 3-Acetoxyyurs-12-en-23-oic acid and agnuside (Fig. 3e, f, Supplementary Fig. 4c, and Supplementary Table 11). It is intriguing that estrone sulfate existed in the difference comparison between the healthy and PCOS groups, indicating its potential link with the progression of PCOS. We observed great similarity between the PCOS-HB and PCOS-LB groups, and the metabolite comparison results between the PCOS-HB and PCOS-LB groups showed that bilirubin, 4-chlorophenol, hydrocinnamic acid, caffeine, quinoline, prostaglandin E2, and PC(2:0/16:1) were the main distinguished metabolite types (Supplementary Fig. 5).

### Association between microbial taxa, metabolites and clinical parameters

**Fig. 5:**
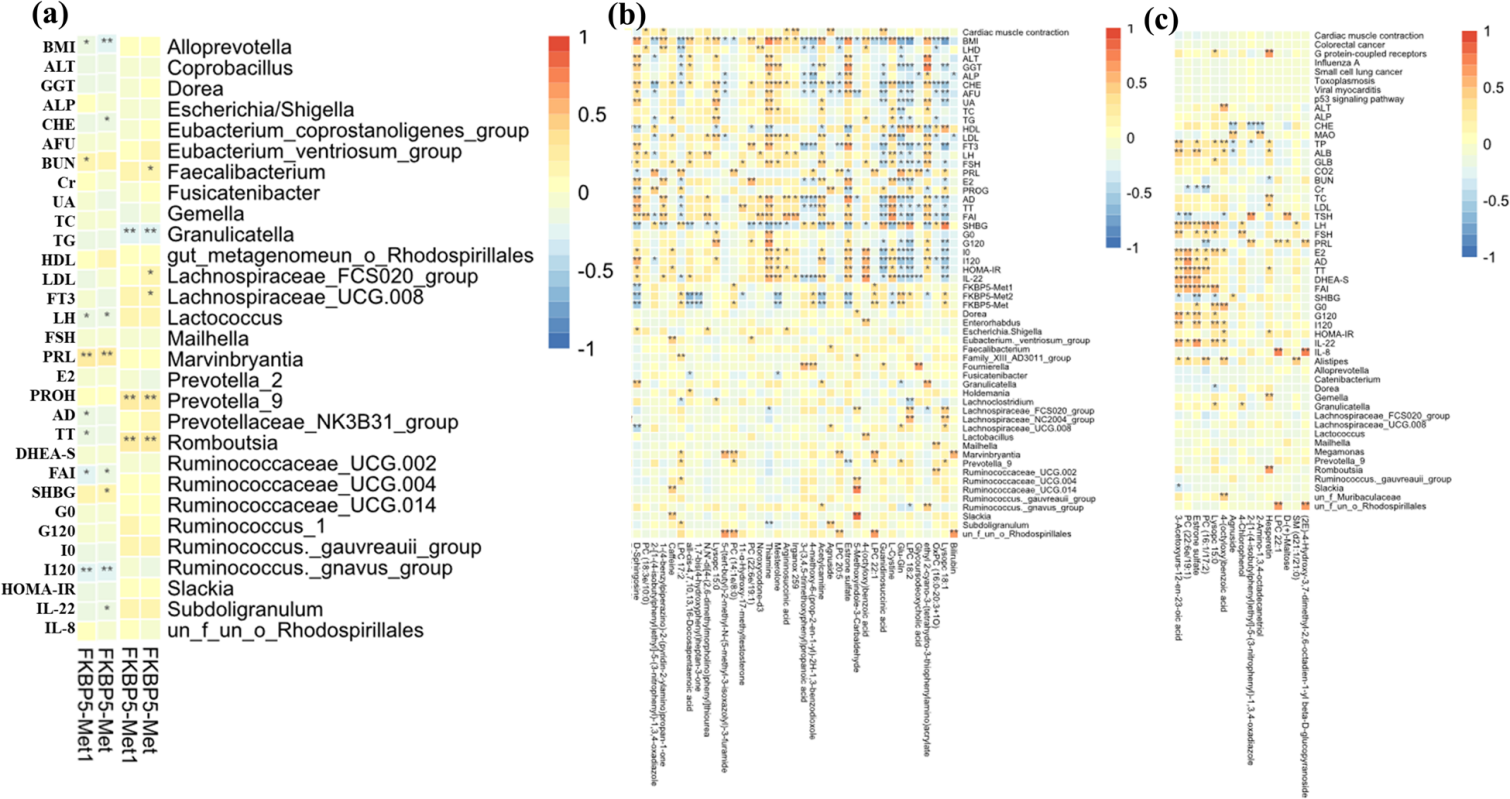
Clinical characteristics correlate with bacterium and pathways. In the heat map, *p < 0.05 denotes significant correlations between pairs. **a** DNA methylation correlation with clinical indices and bacterial taxa. **b** Correlation between metabolites and other factors screened from comparison between healthy patients and patients with PCOS-HB. **c** Correlation between metabolites and other factors screened from comparison between healthy patients and patients with PCOS-LB. PCOS-LB, normal BMI (BMI < 24); PCOS-HB, high BMI (BMI ≥ 24).

**Fig. 6:**
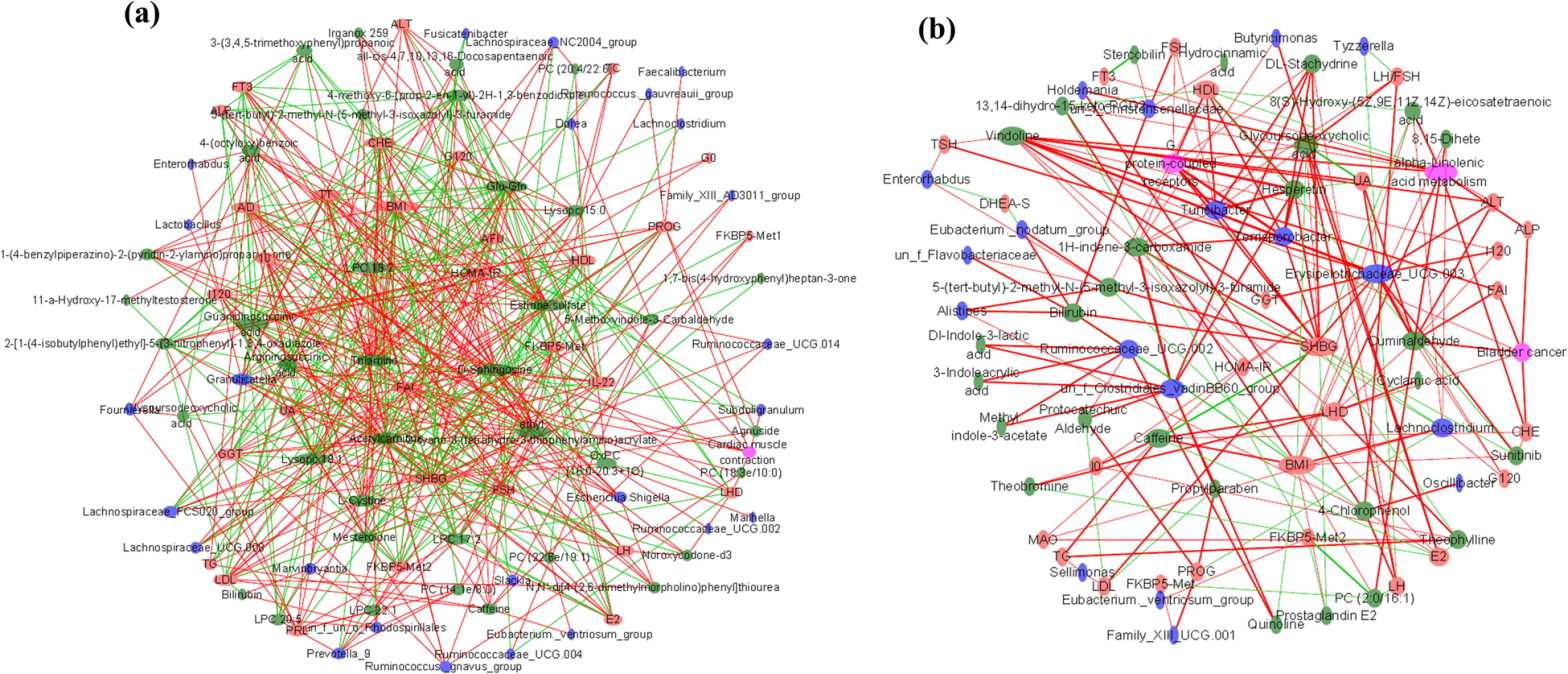
Co-occurrence network. **a** Network characteristics screened from comparisons between healthy patients and patients with PCOS-HB. **b** Network characteristics screened from comparison between PCOS-HB and PCOS-LB groups. Green, blue, red, and pink ellipses denote metabolites, bacteria, clinical parameters, and predicted pathways, respectively. The red and green lines denote positive and negative correlations, respectively. The size of the ellipse denotes the correlation degree. Featured clinical parameters, stress indices DNA methylation, bacterial species, and predicted functional pathways were used. PCOS-LB, normal BMI (BMI < 24); PCOS-HB, high BMI (BMI ≥ 24).

The significantly distinguished clinical properties and gut bacterial taxa were screened out between patients with PCOS and healthy individuals. The relationships between different clinical properties and FKBP5-Met were determined, and the results showed that methylation was positively associated with urea nitrogen (BUN), prolactin (PRL) and sex hormone-binding globulin (SHBG) but negatively related to BMI, cholinesterase (CHE), LH, free androgen index (FAI), and interleukin-22 (IL-22) and I120. In addition, FKBP5-Met displayed a significantly positive correlation with *Faecalibacterium*, Lachnospiraceae, *Prevotella*_9, and *Romboutsia* but was negatively correlated with *Granulicatella* (Fig. 5a). Correlation of featured metabolites with clinical properties and bacterial taxa screened in the comparison between healthy individuals and patients with PCOS-HB indicated that metabolites generally correlated with clinical indices better than bacterial taxa. Bilirubin, LysoPC, OxPC (16:0-20:3 + 1), glycoderodeoxycholic acid, LPC 18:2, Glu-Gln, and LPC 22:1 were negatively related to most clinical parameters while estrone sulfate, acetylcamitine, Irganox, mesterolone, and thiamine were significantly positively correlated with most clinical parameters. It is intriguing that the healthy-abundant taxon, *Prevotella*_9, was positively correlated with Lysopc 18:1, Glu-Gln, LPC 22:1, PC(14:1E/8:0), and LPC 17:2 but was negatively correlated with estrone sulfate. Among all the screened metabolites, estrone sulfate was the only metabolite that was significantly correlated with *Prevotella*_9 (Figs. 5b and 6a). The situation was much different in the correlation of healthy vs PCOS-LB dataset; for example, significantly positive correlation mainly occurred between LH, FSH, estrogen (E2), androstenedione (AD), total testosterone (TT), DHEA-S, FAI, G0, G120, IL-22 and 4-(octyloxy)benzoic acid, Lysopc 15:0, and PC (16:1/17:2), estrone sulfate, PC (22:6e/19:1), and 3-acetoxyurs-12-en-23-oic acid. However, there were no significant correlations between *Prevotella*_9 and all VIP-screened metabolites. In the comparison between PCOS-HB and PCOS-LB groups, bilirubin was significantly related to BMI, SHBG, *Alistipes*, *Eubacterium*, *Ruminococcaceae*, and 4-chlorophenol with LH, E2, *Alistipes*, Holdemania, and *Oscillibacter* and hydrocinnamic acid with free triiodothyronine (FT3). Prostaglandin E2, PC (2:0/16:1) and 13,14-dihydro-15-keto-PGD2 were negatively related to FKBP5-Met2 (Figs. 5c and 6b).

## Discussion

PCOS is a common endocrine disease in females of reproductive age. To date, the exact etiology and pathogenesis of PCOS are unknown. Genetic factors, unhealthy lifestyle, and neuroendocrine, immune and metabolic dysfunctions^31^ are believed to be involved in the pathogenesis of PCOS. Recently, increasing evidence has indicated the gut microbiota characteristics of PCOS patients and linked the gut dysbiosis with the progression of the disease, which helps researchers understand the pathogenesis of PCOS from a new perspective^5,12,13,32,33^. Nevertheless, there have been no reports associating FKBP5 DNA methylation with PCOS and gut microbiota differentiation among PCOS patients with different BMI conditions.

α diversity indices reflect thespecies type and abundance and productivity of the local ecosystem of the intestine. PCOS patients have been shown to have decreased bacterial α diversity compared with that of healthy controls^5,12,13,32–34^, with which our results were consistent. Our results further showed that the Chao1 index is negatively correlated with HOMA-IR, I0 and I120 while positively correlated with level of FKBP5 DNA methylation . Most studies have demonstrated a reduction in gut microbiota diversity and richness in obese subjects^35^. Exposure to social stress also leads to a decrease in diversity in the gut microbiota of mice and Syrian hamsters^36,37^. Our results showed that PCOS patients, especially obese individuals, often have IR and a reduced level of stress-associated FKBP5 DNA methylation. Therefore, these two pathophysiological status may partly explain the decline in gut microbiota diversity.

Some researchers reported that β diversity was significantly different between patients with PCOS and healthy controls^32,38,39^, which was also the case in this study, suggesting that PCOS patients have a specific gut microbiota composition. PCOS patients have been shown to have a higher abundance *of Catenibacterium*, *Kandleria*, Ruminococcaceae, Bacteroidaceae, Parabacteroides, *Clostridium*, *Prevotella*, and *Alistipes*^40^ while a lower abundance of Prevotellaceae^39^. The differences in predominant bacteria in the gut microbiota of PCOS patients have been shown to be significantly affected by race, lifestyle disease severity and sample size. Qi et al.^14^ reported that *Bacteroides vulgatus* content was markedly elevated in the gut microbiota of individuals with PCOS, with reduced IL-22 secretion, which was different from our comparison that was featured as *Prevotella*_9. Several reasons may account for the different results. First, the patients and the control subjects in Qi’s study were located in northern China, but the subjects in our study were located in southeastern China. The differences in lifestyle expecailly diet and anthropometrics between northern and southern China are very substantial. It has been reported that diet^7,41,42^, drug useand anthropometrics together explain 20% of gut microbiota variability^43^. Regarding dietary habits, Yamashita et al.(2019) reported that native Japanese and Japanese-American individuals had similar genetic backgrounds but different diets that led to gut microbiota composition changes^44^. Second, the characteristics, including BMI and HOMA-IR, of the recruited PCOS patients and the control individuals were somewhat different from those in Qi’s and our study. For individuals without PCOS, a higher abundance of Firmicutes in those with obesity was observed, and the Firmicutes/Bacteroidetes ratio seemed to be higher in women with a high BMI^45–47^. This ratio was reduced with a decrease in Firmicutes and an increase in Bacteroidetes abundance after bariatric surgery and weight loss^48^. However, some studies have pointed out that the decrease of Bacteroidetes abundance is accompanied by an increase in Actinobacteria rather than Firmicutes abundance^49^, or a synchronous increase in Firmicutes and Actinobacteria abundance. A meta-analysis found that no significant difference in the Bacteroidetes*-*to*-*Firmicutes ratio between obese and lean rodents^50^.

Furthermore, we paid attention to gut microbiota compositions in PCOS patients with different BMI levels. Liu et al.^17^ found that *Bacteroides*, *Escherichia/Shigella*, and *Streptococcus* were positively correlated with BMI while *Akkermansia* and Ruminococcaceae were negatively correlated with BMI. Adolescents who had PCOS and obesity were found to have a higher abundance of Actinobacteria, lower abundance of Bacteroidetes, and similar abundances of Firmicutes and Proteobacteria compared with those of controls who had obesity^5^. Zeng et al.^39^ found that Prevotellaceae abundance was dramatically lower in PCOS patients, especially in the IR-PCOS group. *Lachnoclostridium*, *Fusobacterium*, *Coprococcus_*2, and *Tyzzerela* 4 were found to be the characteristic genera of patients with PCOS and obesity^34^. Our results showed that there were obvious differences in α and β diversities between normal subjects and PCOS patients, but the difference was not obvious between PCOS patients with elevated BMI and normal BMI. These results suggest that the effect of PCOS on the gut microbiota is greater than that of BMI.

PCOS women tend to experience mildly elevated anxiety and depression significantly more often than women without PCOS^51–53^ and to have a higher level of perceived stress^25^. Whether these unhealthy psychological states are the results of PCOS or one of the reasons that cause or deteriorate PCOS is still unknown. Patients with PCOS and obesity have been found to have higher depression scores than women with PCOS who do not have obesity, and depression scores were found to be significantly correlated with IR and lipid parameters and with the number of metabolic syndrome components^24^. Livadas et al.^54^ showed that the degree of anxiety, state, and trait (STAI-S, STAI-T) appeared to vary in a pattern similar to that of hyperandrogenemia and IR independent of age and BMI in 130 PCOS patients. FKBP5 is an important modulator of stress responses. Aging/stress-driven FKBP5-NF-κB signaling mediates inflammation, potentially contributing to cardiovascular risk, and may thus point to novel biomarkers and treatment possibilities^55–59^. Furthermore, FKBP5 is also a positive regulator of the androgen receptor^30^, and this mechanism may be related to the incidence and severity of PCOS. Our study showed that patients in the PCOS-HB group had the lowest levels of FKBP5-Met compared with the PCOS-LB and control groups. The level of FKBP5 DNA methylation was proved to be associated with age and stress. In our study, the patients with PCOS had a narrow range of ages and there was no difference in age among groups. Therefore, the level of FKBP5 DNA methylation was more obvious in stress-associated individuals than in age-associated individuals. In our study, the results of FKBP5 DNA methylation analysis suggested that patients with a high BMI had more stress than the patients with a normal BMI. Stress may be related at least in part to certain clinical features of PCOS, including obesity and hirsutism^60^ or an awareness of PCOS^61^. Furthermore, a low level of FKBP5-Met would lead to higher expression of FKBP5 protein and increased androgenic activity, which might be involved in the occurrence and development of PCOS in patients who have a high BMI.

The gut microbiota composition changes as a response to stressful situations and interventions that can modulate microbiota stress in human and animal models^26,27^. Stressor exposure was shown to decrease the relative abundance of bacteria in the genus *Bacteroides* while increasing the relative abundance of bacteria in the genus *Clostridium* in mice^62^. Mice colonized with gut microbiota from stressed mice with a lower relative abundance of *Lactobacillus* and a higher relative abundance of *Akkermansia* showed similar behaviors^63^. Probiotics as interventions can improve anxiety symptoms^64^. Overall, stress may be involved in PCOS in multiple ways. Among them, an important factor to target may be the composition of the gut microbiota, while enhancing androgen receptor activity through FKBP5 may be another option.

In the nontargeted metabolomics in our study, we found that metabolic products, especially lipid profiles, were different among the PCOS-LB, PCOS-HB, and control groups. Li et al.^65^ reported that polyunsaturated fatty acids levels were reduced and that long-chain saturated fatty acids levels were increased in patients with PCOS and obesity compared with that in lean controls. We screened the differentially abundant metabolites that could be linked with gut microbiota to explore the possible pathogenesis. On the other hand, our findings can be used as a potential basis for disease diagnosis. For example, Daan et al.^66^ showed that retinol-binding protein 4,(RBP-4), dipeptidyl peptidase IV(DPP-IV), and adiponectin, as potential discriminative markers for PCOS with obvious hyperandrogenemia, had a specifically strong correlation in cases with a higher BMI. Our results showed that estrone sulfate, which is the most abundant estrogen precursor found in the bloodstream of women and men^67,68^, was notable in the difference comparison between the healthy and PCOS groups, indicating its potential role in the progression of PCOS.

Our study had limitations. First, although it included healthy individuals with normal BMI, additional healthy participants with a high BMI are also be needed to reveal the background difference between healthy subjects with a normal BMI and those with a high BMI. Second, our trial needs to be repeated in other geographical locations since microbiome composition is affected by ethnicity and diet. Third, it is difficult to conclude whether the change in FKBP5 gene methylation and gut microbiota composition is the cause or the result of PCOS, as most data were not functionally validated, the characteristic biomarkers should be verified in the future to reveal their clinical potential in disease diagnose. Lastly, we mainly used amplicon-based metagenomics and nontargeted metabolomics tools, and shotgun metagenome sequencing and lipidomics strategies could be used further to supply better characteristic resolution.

In conclusion, based on multi-omics data from patients with PCOS and healthy controls, our results suggest that PCOS patients with different BMI levels have differentially altered stress responses, gut microbiota compositions, and metabolites. The association between the top contributing genus, *Prevotella*_9, and the metabolite, estrone sulfate, in the comparison between patients with PCOS and healthy controls revealed their potential involvement in PCOS which needs further causal verification. In addition, the close connection among the stress-associated index, FKBP5-Met, and the PCOS-related phenotype may provide a therapeutic target.

## Materials and methods

### Human participants

The study and all experimental procedures were approved by the Ethics Committee of the First Affiliated Hospital Shantou University Medical College according to the Council for International Organizations of Medical Science (ChiCTR2000041108). All participants were recruited from the Department of Endocrinology at the First Affiliated Hospital Shantou University Medical College between June 2019 and September 2020. Written informed consent was obtained from all participants.

The healthy volunteers who had regular menstrual cycles and normal ovarian morphology were from the general community. Their hormone levels, BMI, glucose tolerance status, blood pressure and serum lipids were all in the normal ranges. Women who were breastfeeding or pregnant within the past year were excluded from the study. Women with PCOS were diagnosed according to the 2003 Rotterdam criteria, which require the presence of at least two of the following: (1) oligo-ovulation and/or anovulation; (2) clinical and/or biochemical signs of hyperandrogenism; and (3) ultrasound findings of polycystic ovaries in 1 or 2 ovaries, ≥12 follicles measuring 2–9 mm in diameter, and/or ovarian volume ≥10 mL. Diagnoses of PCOS were made after the exclusion of other etiologies for hyperandrogenemia or ovulatory dysfunction^8^. All PCOS patients were first-visit patients and had not received PCOS-related treatment.

### Clinical parameters determination

Ninety-eight PCOS patients with a normal BMI (PCOS-LB, BMI < 24), 50 PCOS patients with high BMI (PCOS-HB, BMI ≥ 24), and 38 healthy individuals with a normal BMI were recruited from the First Affiliated Hospital Shantou University Medical College Hospital between September 2018 and July 2020. All the participants were asked to come to our department during days 2–4 of spontaneous cycles after an overnight fast. Height, body weight, and BMI were calculated. Blood pressure was measured after at least 15 min of rest. Peripheral blood samples were collected from all subjects for TT, AD, DHEA,DHEA-S, E2, SHBG, LH, FSH, PRL, progesterone (PROG), FT3, free thyroxine (FT4), thyrotropin (TSH), G0, I0, and biochemical indexes included liver function, kidney function, and blood lipid measurement. Blood was also collected for plasma metabolomics and FKBP5 DNA methylation assays. The oral glucose tolerance test (OGTT) and insulin-releasing test were performed on the same day. After the procurement of the first blood sample, within 5–10 min, all the subjects ingested a solution containing 75 g glucose diluted in 300 mL of water. Subsequently, one additional blood sample was obtained at 120 min after the ingestion of the solution to estimate the glucose level (G120: glucose level after 120 min) and insulin concentrations (I120: insulin level after 120 min). SHBG was measured using a luminescence immunoassay (Siemens, New York, USA). The levels of serum FSH, LH, PRL, E2, PROG, FT3, FT4, and TSH were tested by radioimmunoassay (Beckman, MN, USA). Serum insulin concentration was estimated using a direct chemiluminometric assay (Siemens, New York, USA). A liquid chromatography (LC) (ACQUITY UPLC I Class, Water, MA, USA) coupled to tandem mass spectrometry (MS) (Triple Quad™5500, AB SCIEX, MA, USA) system was used to quantitate TT, AD, DHEA, and DHEA-S with the multiple reaction monitoring (MRM) model in BGI (Shenzhen, China). IL-22 and IL-8 was measured by ELISA (R&D, Minneapolis, MN, USA). The levels of plasma glucose and biochemical indexes included liver function, kidney function, and blood lipid were measured using an autoanalyzer (Beckman Coulter AU5800, MN, USA). HOMA-IR was defined as I0 (mIU/L) × G0 (mmol/L)/22.5. The free androgen index (FAI) was calculated with the formula FAI = TT (ng/mL) × 100 × 3.467/SHBG (nmol/L).

### DNA methylation determination

FKBP5 methylation of CpG sites cg20813374 and cg00130530 (Chr6: 35657180 and Chr6:35657202, respectively; Assembly: hg19) was assessed by targeted bisulfite pyrosequencing according to a previous study^55^. They used Illumina HumanMethylation450 BeadChip (450 K) data from three independent cohorts with broad age range and documented stress related phenotypes and analyses included all available CpGs covered by the 450 K within or in close proximity (10 kb upstream or downstream) to the FKBP5 locus (chromosome 6p21.31). After controlling for potential confounders and after false discovery rate (FDR) correction for multiple comparisons, these two CpGs showed consistent and robust age-related decrease in methylation across all cohorts^55^. Genomic DNA was extracted from whole blood using the Gentra Puregene Blood Kit (QIAGEN, GmbH, Hilden). Genomic DNA (500 ng) was bisulfite converted using the DNA Methylation Kit (QIAGEN, GmbH, Hilden) with 140 μL reaction mix of bisulfite, DNA protective solution, and RNase-free water. After 25 min at room temperature, DNA was transformed in the ABI 9700 PCR System (Applied Biosystems) with the conditions of 95 °C 5 min, 60 °C – 25 min, 95 – 5 min, 60 °C – 85 min, 95 °C – 5 min, 95 °C – 175 min, and 4 °C – ∞ . Bisulfite-converted DNA was amplified in a 50 µL reaction mix (40 µL DNA; each bisulfite-specific primer with a final concentration of 0.2 µM, FKBP5-F: 5′-TTGGTTAGGTTAGTTTTAGGAAGTAAT-3′ and FKBP5-R-biot: biotin 5′-ACCAAAAAAAAATATAATCTTTACAATCAC-3′) using Taq (KAPA). The cycling conditions of the touchdown PCR were 95 °C for 3 min, 40× (94 °C – 30 s, 54 °C – 30 s, 72 °C – 60 s), 72 °C for 7 min, and cooling to 4 °C. Pretreatment of PCR amplicons was facilitated with the PyroMark Q96 Vacuum Workstation (QIAGEN GmbH, Hilden). Sequencing of FKBP5 CpGs methylation was performed on a PyroMark Q96 ID system (QIAGEN GmbH, Hilden) using PyroMark Gold Q96 reagents and the following sequencing primer: FKBP5-S (CpG 35657180, 35657202/hg19): 5′-AAGTAATTTTATTAAGTTTAAGATG-3′. Pyro Q-CpG Software (QIAGEN GmbH, Hilden) was used for data analysis. The methylation results of CpG sites at Chr6: 35657180 and Chr6:35657202 were expressed as FKBP5-Met1 and FKBP5-Met2 respectively. FKBP5-Met was the average level of FKBP5-Met1 and FKBP5-Met2.

### 16S rRNA gene sequencing and data analysis

Fecal samples from healthy individuals and PCOS patients were collected on the day of the medical examination and immediately frozen at −80 °C. Fecal microbial DNA was extracted from approximately 200 mg of the fecal samples using HiPure Stool DNA Kits B (D3141-03B, Guangzhou Meiji Biotechnology Co., Ltd., China) according to the manufacturer’s protocols. The purity and concentration of the isolated DNAs were assessed by Qubit3.0 Fluorometer (Invitrogen, Carlsbad, CA, USA). DNA samples were stored at −20 °C before being used as templates for 16S rRNA gene sequencing library construction. The 16S rRNA gene was amplified from the DNA samples with barcoded forward primers (5′-CCTACGGRRBGCASCAGKVRVGAAT-3′) and reverse primers (5′-GGACTACNVGGGTWTCTAATCC-3′), which were designed by GENEWIZ (Suzhou, China) (Supplementary Table 1), and aimed at relatively conserved regions bordering the V3 and V4 hypervariable regions of bacteria and Archaea 16S rDNA^69,70^. DNA library concentrations were validated by Qubit3.0 Fluorometer and further multiplexed and loaded on an Illumina MiSeq instrument according to the manufacturer’s instructions (Illumina, San Diego, CA, USA). The bioinformatics analysis procedure for raw reads was performed according to previously described methods^71,72^. The sequence data will be available at NIH Sequence Read Archive under SUB8691740.

### Untargeted metabolomics measured by LC-MS/MS

Plasma samples (100 μL) and prechilled methanol (400 μL) were mixed by vortexing. LC-MS/MS analyses were performed using a Vanquish UHPLC system (Thermo Fisher) coupled with an Orbitrap Q Exactive series mass spectrometer (Thermo Fisher)^73^. Identification and quantification of metabolites were performed using the mzCloud database by the search engine Compound Discoverer 3.0 (Thermo Fisher Scientific). Partial least squares discriminant analysis (PLS‐DA) and featured metabolites based on variable importance in projection (VIP) scores were performed at MetaboAnslyst (https://www.metaboanalyst.ca/). We applied univariate analysis to evaluate significance. The metabolites with VIP > 1, *p* < 0.05 and FC ≥ 2 or FC ≤ 0.5 were considered to be distinguishingly featured metabolites.

### Statistical analysis

Bacterial diversity was determined by sampling-based OTU analysis and is presented by Shannon, Simpson, Chao1, ACE, observed species, and Pielou’s evenness (J) index, which was calculated using the R program package ‘vegan’ (version 2.5.6). Bray–Curtis distance-based β-diversity metrics were obtained with vegdist and PERMANOVA with the Adonis function, and analysis of similarity (ANOSIM) was conducted to compare the bacterial differences among different sample subgroups. The shared OTUs were calculated and visualized using the R package VennDiagram (version 1.6.20). The significantly distinguished taxa and predicted pathways by PICRUSt were screened by comparison between the PCOS and healthy groups by the Wilcoxon test. LEfSe analysis was performed to identify taxa with differentiating abundance in the different groups^74^. Pearson’s correlation between the abundances of differential genus taxa and pathways was computed by the R package stats (version 3.6.0), and the package pheatmap (version 1.0.12) was used to conduct the correlation heatmap. The network graphs were made using Cytoscape.

### Reporting summary

Further information on research design is available in the Nature Research Reporting Summary linked to this article.

## Supplementary information

Supplementary information

Reporting Summary

## Data Availability

The dataset supporting the conclusions of this article is available in the NCBI Sequence Read Archive repository under the accession number PRJNA737206. Code and scripts used in the analyses are available upon request.
