# Supplementary material for: Reduced stress-associated FKBP5 DNA methylation together with gut microbiota dysbiosis is linked with the progression of obese PCOS patients
Source: NPJ Biofilms Microbiomes. 2021 Jul 15;7:60. doi: 10.1038/s41522-021-00231-6 (PMC8282850; doi:10.1038/s41522-021-00231-6)
Supplement: Supplementary file 1 — Supplementary information [file 41522_2021_231_MOESM1_ESM.pdf]

Reduced stress-associated FKBP5 DNA methylation together with gut microbiota dysbiosis is linked with the progression of obese PCOS patients

Fu Chen[1]*, Zhangran Chen[2]*, Minjie Chen[3,4], Guishan Chen[3], Qingxia Huang[3], Xiaoping Yang[3], Huihuang Yin[3,4], Lan Chen[3], Weichun Zhang[3], Hong Lin[5], Miaoqiong Ou[1], Luanhong Wang[6], Yongsong Chen[3], Chujia Lin[3], Wencan Xu[3], Guoshu Yin[3#]

[1]Department of Clinical Nutrition, The First Affiliated Hospital of Shantou University Medical College, Shantou, Guangdong Province, China. 515041

[2]Institute for Microbial Ecology, School of Medicine, Xiamen University, Xiamen, China

[3]Department of Endocrinology, the First Affiliated Hospital of Shantou University Medical College, Shantou, Guangdong Province, China. 515041

[4]Laboratory of Molecular Cardiology and Laboratory of Molecular Imaging, the First Affiliated Hospital of Shantou University Medical College, Shantou, Guangdong Province, China. 515041

[5]Department of Department of Reproductive Center, the First Affiliated Hospital of Shantou University Medical College, Shantou, Guangdong Province, China. 515041

[6]Department of Gynecological tumor, Tumor Hospital Affiliated to Shantou University Medical College, Guangdong Province, China. 515031

*Fu Chen and Zhangran Chen contributed to this study equally.

Correspondence Author

[#]Guoshu Yin, Email: yinguoshu@126.com.

Supplemental Figures

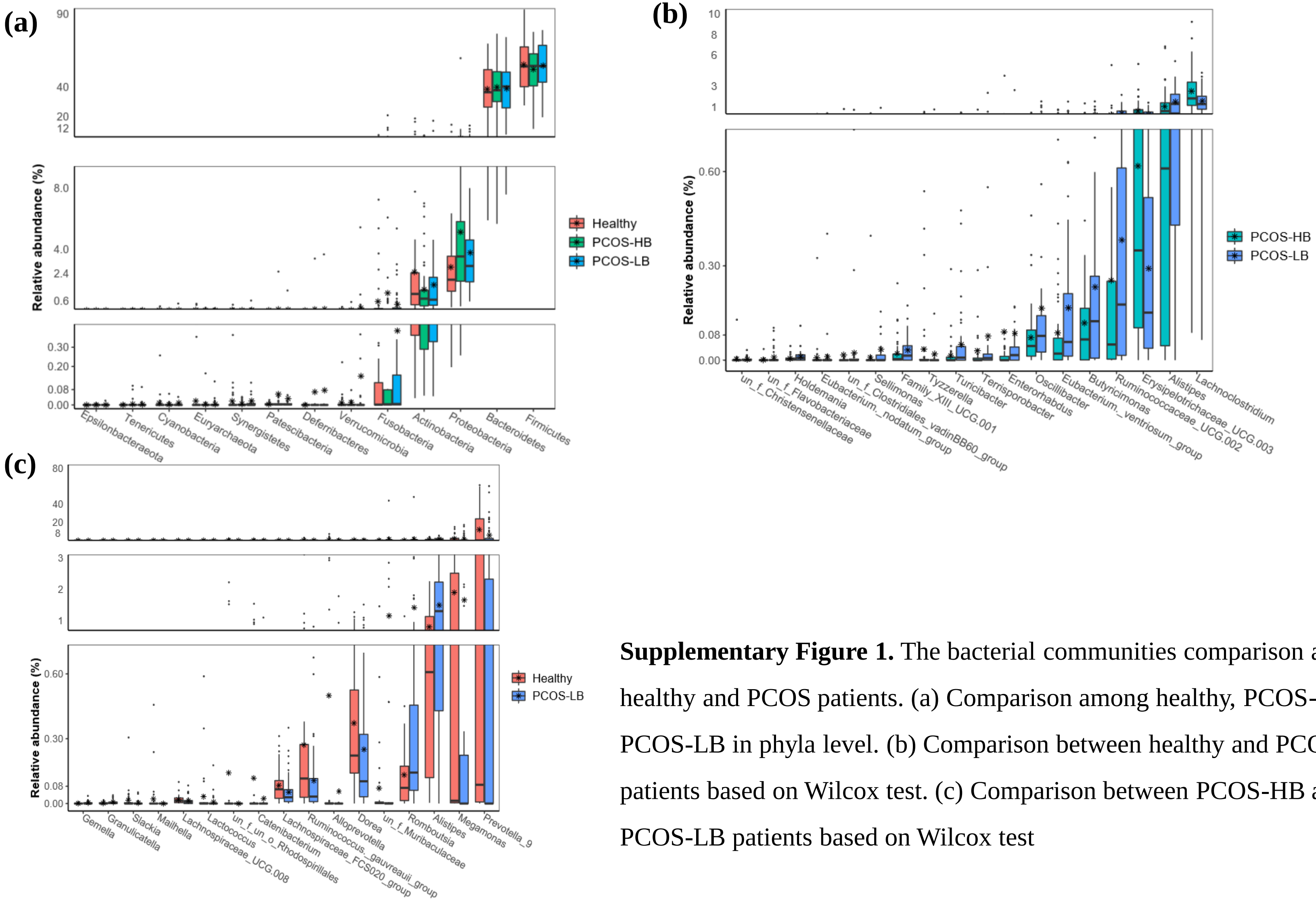

Supplementary Figure 1. The bacterial communities comparison among healthy and PCOS patients. (a) Comparison among healthy, PCOS-HB and PCOS-LB in phyla level. (b) Comparison between healthy and PCOS-LB patients based on Wilcox test. (c) Comparison between PCOS-HB and PCOS-LB patients based on Wilcox test

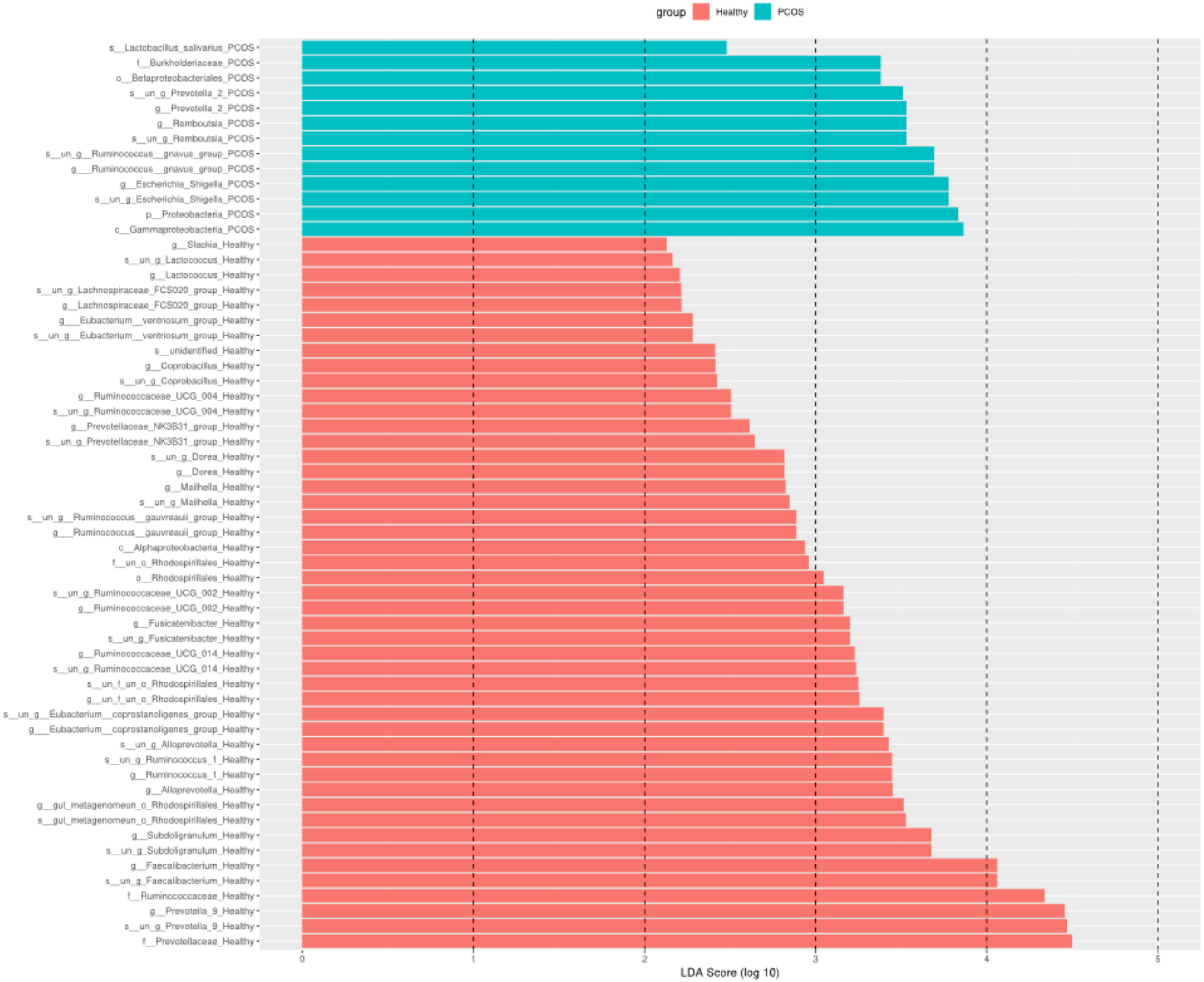

Supplementary Figure 2. Reveal of characteristic bacterial taxa based on LDA Effect Size (LEfSe) analysis

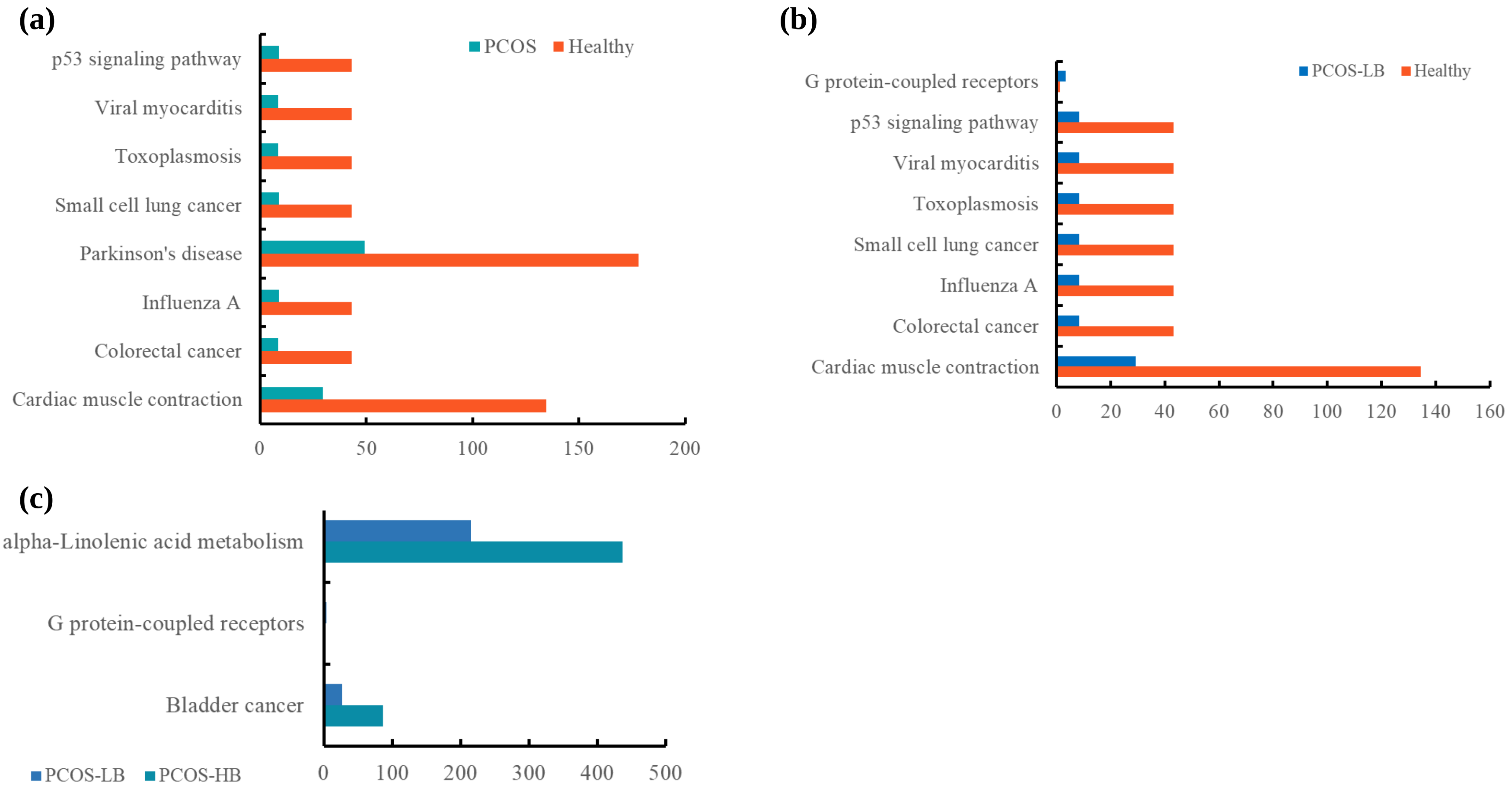

Supplementary Figure 3. The predicted pathway comparison among healthy and PCOS patients. (a) Comparison between healthy and PCOS. (b) Comparison between healthy and PCOS-LB patients. (c) Comparison between PCOS-HB and PCOS-LB patients

(a)

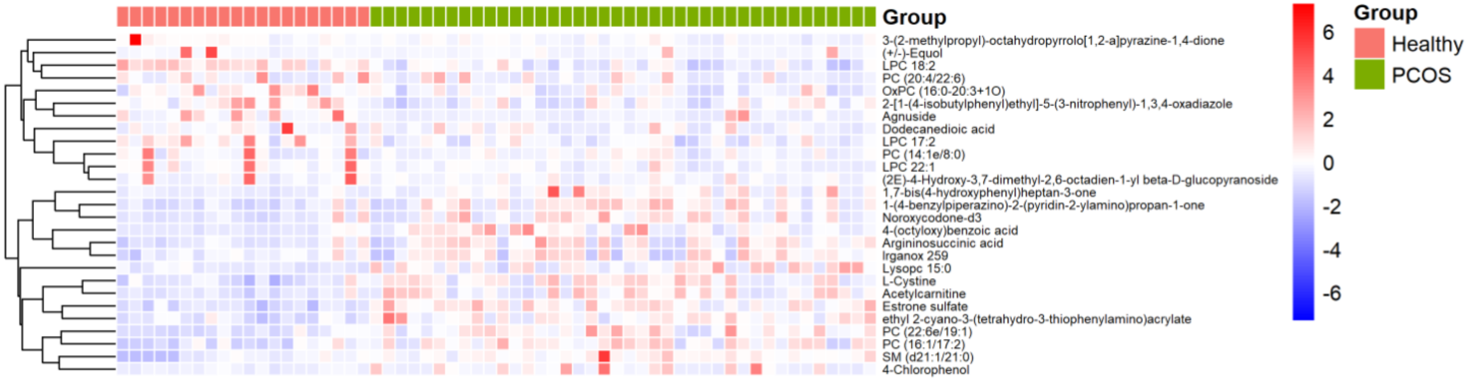

(b)

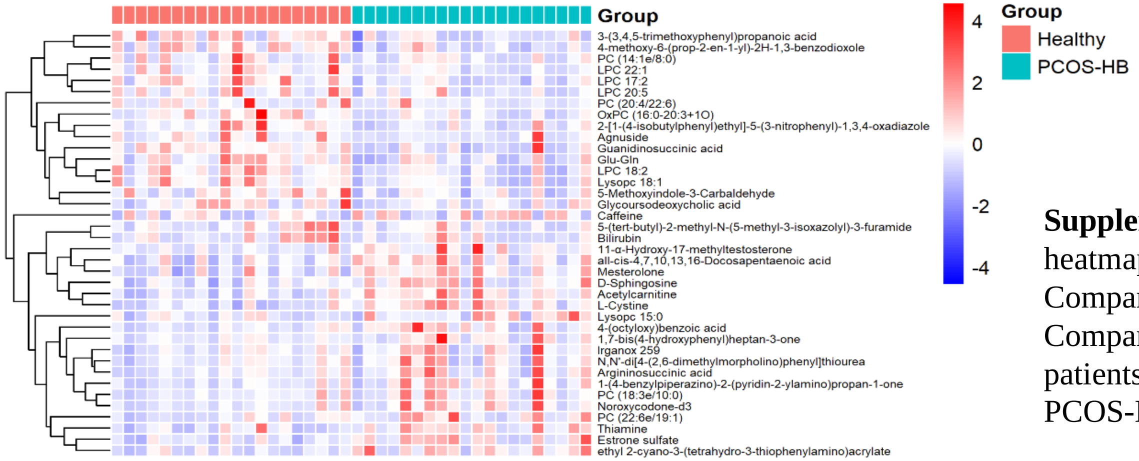

(c)

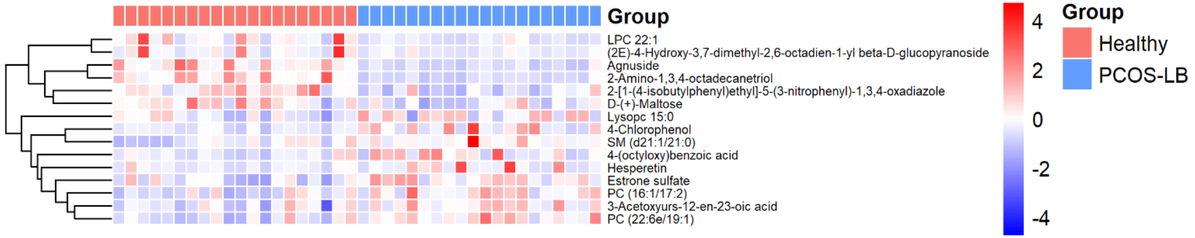

Supplementary Figure 4. The distinguished metabolites heatmap among healthy participants and PCOS patients. (a) Comparison between healthy participants and PCOS. (b) Comparison between healthy participants and PCOS-HB patients. (c) Comparison between healthy participants and PCOS-LB patients

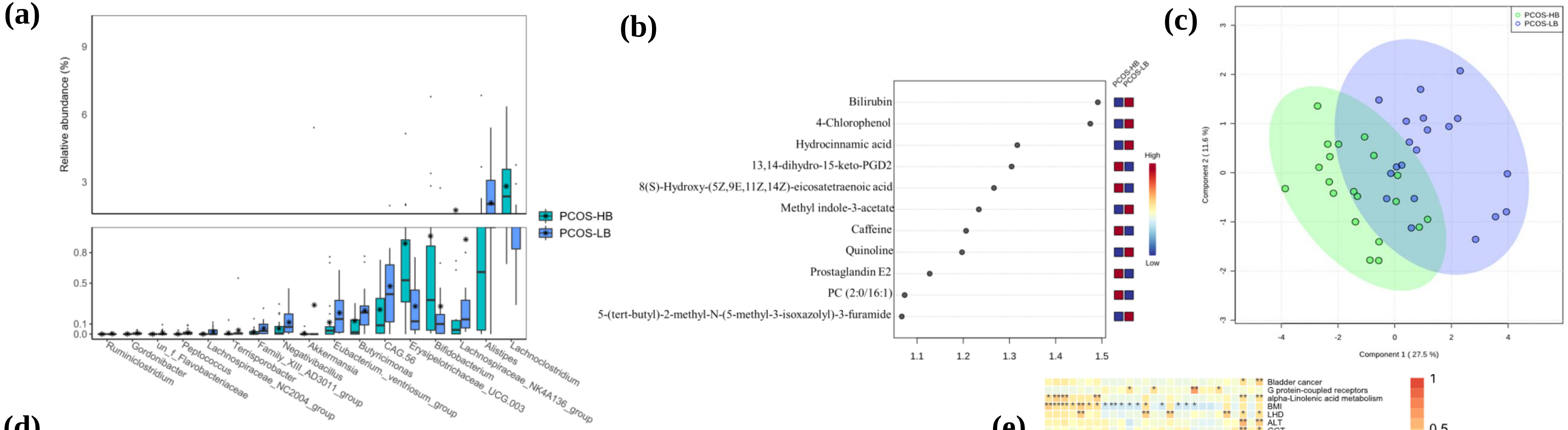

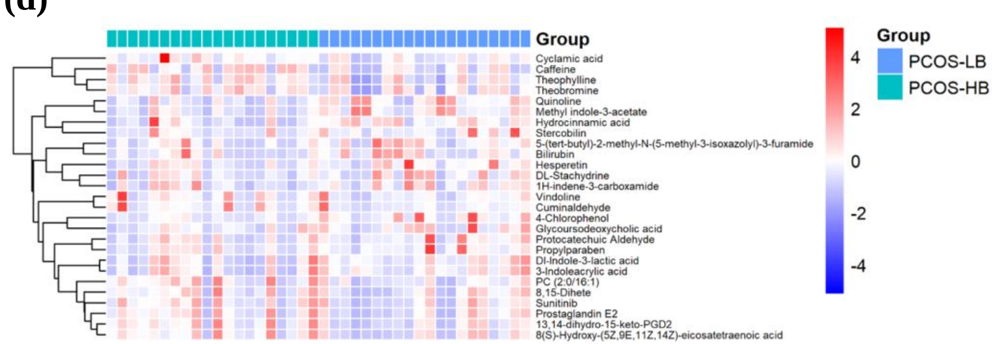

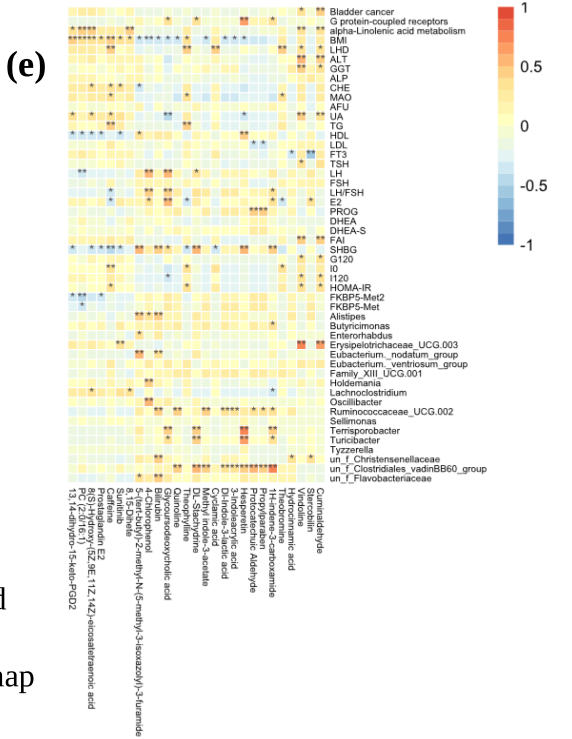

Supplementary Figure 5. The paired comparison of bacterial taxa, metabolites and their association between PCOS-HB and PCOS-LB group. (a) PLS-DA plot revealing the metabolites composition of extreme high or low BMI PCOS patients. (b) Comparison of characterized bacterial genus. (c) VIP scores of PLS-DA in metabolites comparison. VIP scores were used to rank the discriminating power of different taxa between the PCOS and control groups. A taxon with a VIP score of >1 was considered important in the discrimination. (d) Comparison of characterized metabolites profile. (e) Correlation heatmap of distinguished clinical parameters, bacterial taxa, predicted pathway with metabolites

Supplementary Table 1 All primers used in this study

Primer	Sequence
FKBP5-F	5'-TTGGTTAGGTTAGTTTTAGGAAGTAAT-3'
FKBP5-R-biot	5'-ACCAAAAAAAAATATAATCTTTACAATCAC-3'
FKBP5-S	5'-AAGTAATTTTATTAAGTTTAAGATG-3'
16S Forward	5’-CCTACGGRRBGCASCAGKVRVGAAT-3’
16S Reverse	5’-GGACTACNVGGGTWTCTAATCC-3’

Supplementary Table 2 Sequence process information about PCOS patients and healthy individuals

sample	RawReads	CleanReads	Chimerta_check	OTUseqs	OTUs
N1	60728	60236	50381	49758	235
N2	53766	53043	48362	47993	304
N3	64144	63563	52604	52133	284
N4	47953	47488	38800	38548	276
N5	74329	73595	61483	60378	183
N6	79453	78691	69430	69202	150
N7	63922	63273	57654	57475	259
N8	46677	46279	39071	37782	271
N10	79831	79160	70872	70080	276
N11	79365	78279	72885	72404	323
N12	63957	63077	55257	54778	225
N14	100238	98787	83717	83307	271
N15	88085	87338	72052	69116	277
N16	64363	63708	55166	55005	232
N17	60300	59768	50352	49628	293
N18	43976	43462	39762	39677	254
N19	61260	60502	56334	55914	278
N20	41042	40766	35079	34804	196
N21	108354	107454	97018	96595	282
N22	93267	92471	84032	83159	216
N23	45095	44719	39535	38738	186
N24	62916	62415	52162	48983	162
N26	57159	56587	50299	50186	255
N27	77341	76700	65207	63897	206
N28	57060	56514	50159	49984	207
N29	36666	36332	31499	31080	248
N30	65593	65091	58491	57939	201

N31	59493	59014	51098	50671	262
N32	69313	68722	60112	59848	223
N33	57886	57191	52841	52711	258
N34	43979	43552	37377	36750	235
N35	59271	58702	56084	55950	277
N36	52803	52278	45736	45291	214
N37	58743	57992	54607	54245	280
N38	65296	64779	51546	51078	245
N39	66358	65745	54682	53671	284
N40	77675	76896	64314	63271	257
N41	77745	76890	60865	58463	296
P001	60603	59622	54599	48047	236
P002	51546	50460	44964	43218	155
P003	43392	42892	40243	39552	183
P004	57626	56766	51027	51018	136
P005	62700	59702	53123	53056	219
P006	82465	81694	75214	74520	306
P007	72566	71701	67717	67646	166
P008	59863	59473	53713	53105	172
P009	65286	64665	55764	54636	300
P010	57053	56483	52095	52000	123
P012	76360	72793	56255	55750	176
P013	69607	68965	64075	62524	240
P014	60066	59463	56090	54952	277
P015	86286	84579	74737	72142	268
P016	89937	88164	75483	75033	232
P017	91151	89204	75339	73853	307
P018	66183	64285	54818	54121	194
P019	88241	86188	69596	69100	314

P020	87471	85601	73019	72270	246
P021	78898	77327	66436	65881	284
P022	78570	77902	69936	69355	274
P023	99708	98174	90082	83421	247
P024	81761	80481	75685	73869	227
P025	130218	128246	112565	110405	145
P026	70586	66733	58496	58048	102
P027	69325	67623	62158	61983	168
P028	94935	93443	84646	84024	255
P030	109095	107943	101887	102074	171
P031	135710	134077	116505	115493	270
P033	94714	93011	82712	82691	213
P034	89332	85341	75639	75588	156
P035	76044	74845	67907	67436	288
P036	58991	58242	54888	54602	224
P037	87183	86007	65747	64826	259
P038	75692	74905	66890	66376	285
P039	54060	53122	45924	45898	171
P040	67342	66515	59960	58562	208
P041	81333	79472	72633	71415	244
P042	40173	39515	35342	35270	204
P043	59875	59477	55598	55334	247
P044	63768	62331	54688	54583	248
P045	109309	107627	99105	98939	234
P046	58101	56993	52103	52005	141
P047	85414	84026	71952	71967	128
P048	49516	49060	44246	43997	270
P049	60840	59583	53438	53323	236
P050	58283	57983	57152	57158	83

P051	46348	45805	42199	42112	201
P052	69290	68275	58779	57196	193
P053	100418	99371	88284	87801	179
P054	55838	54558	49926	49921	200
P056	110680	109129	99423	98440	224
P057	52525	51830	48044	46038	201
P058	76064	74824	61901	60973	187
P059	63078	62294	58026	56996	256
P060	62548	61671	57360	56894	248
P061	72575	71096	58297	57928	219
P062	67812	66460	53505	52478	289
P063	91879	90988	70550	70164	236
P064	64723	63616	49316	48974	264
P065	73101	70624	59086	59135	104
P066	51670	51124	45610	45467	191
P067	63195	62551	51780	51263	148
P068	74976	74343	62469	62080	218
P069	64419	63688	60109	60016	224
P070	64562	63950	56084	55802	186
P071	57952	57070	50263	50097	251
P072	55254	54683	47521	47259	236
P073	79629	78488	66471	66107	165
P074	57856	57275	47027	46745	227
P075	48837	48385	39419	37598	220
P076	62873	62200	57146	57081	220
P077	60491	59941	51039	49720	240
P078	75512	74882	63491	63077	301
P079	76030	75337	61303	60837	294
P080	52943	52497	44792	43705	244

P081	67360	65584	62534	61703	246
P082	65204	64733	55625	53247	167
P083	75214	74595	62756	62540	221
P084	76606	75795	66936	66855	198
P085	62480	61830	52660	52522	197
P086	61056	60479	51698	48856	259
P087	69699	68966	64532	64489	190
P088	63047	62418	55799	55685	226
P089	63978	63415	55375	55074	222
P090	70883	70206	60702	60463	298
P092	80025	79302	71312	70268	296
P093	68841	68126	57673	57031	221
P094	72012	71330	63786	63650	207
P095	54436	53931	48302	48264	176
P096	63228	62408	57606	57456	201
P097	74092	73472	65592	65112	236
P099	52041	51600	46581	45598	250
P100	59783	59150	52898	52260	213
P101	69905	69186	63695	62961	268
P102	67300	66793	61212	61056	225
P103	68755	68203	58092	57938	212
P104	63956	63492	58095	57544	153

Supplementary Table 3 Bacterial diversity among PCOS patients and healthy individuals

sample	Group	Observe	Chao1	ACE	Shannon	Simpson	J
N1	Healthy	210	248.154	236.246	3.62963	0.94912	0.6788
N2	Healthy	285	312.774	317.849	4.19086	0.97301	0.74142
N3	Healthy	270	323.04	314.833	2.92546	0.80639	0.52255
N4	Healthy	269	305.964	309.756	3.48794	0.92234	0.62343
N5	Healthy	165	213.462	211.159	3.42058	0.9348	0.66992
N6	Healthy	128	171.5	164.685	3.03	0.9091	0.62448
N7	Healthy	225	281.438	265.206	3.60898	0.93859	0.66634
N8	Healthy	260	277.4	280.042	3.76349	0.942	0.6768
N10	Healthy	230	273.333	267.221	3.75815	0.95201	0.69108
N11	Healthy	292	309.826	325.693	4.18375	0.97059	0.737
N12	Healthy	203	266.588	257.085	3.20625	0.90794	0.60345
N14	Healthy	209	252.75	268.472	3.02925	0.90121	0.56703
N15	Healthy	249	318.176	295.781	3.55646	0.92192	0.64458
N16	Healthy	212	261.583	238.41	3.00854	0.8746	0.56165
N17	Healthy	262	309	306.438	3.675	0.93132	0.65998
N18	Healthy	245	296	286.228	2.99655	0.87626	0.5447
N19	Healthy	258	328.5	303.3	3.72563	0.92585	0.67093
N20	Healthy	195	269.1	235.514	3.59911	0.95076	0.68255
N21	Healthy	249	259.833	264.509	3.76821	0.9455	0.68296
N22	Healthy	187	233.75	217.202	3.36643	0.93707	0.64354
N23	Healthy	179	194.615	204.283	2.83667	0.87129	0.54684
N24	Healthy	148	206	179.798	3.33618	0.93637	0.66761
N26	Healthy	225	260.455	255.23	2.42326	0.70846	0.44742
N27	Healthy	172	195	190.087	3.41972	0.9186	0.66435
N28	Healthy	192	265.929	252.427	3.54036	0.9494	0.67339
N29	Healthy	248	279.364	291.201	2.82701	0.82944	0.51275
N30	Healthy	184	221.714	217.338	3.37138	0.93339	0.64649
N31	Healthy	235	293.235	279.766	3.9072	0.96189	0.71566
N32	Healthy	192	231	236.674	3.26906	0.90313	0.62179
N33	Healthy	238	264.037	267.221	3.5499	0.94022	0.64871
N34	Healthy	227	315.773	305.056	2.76116	0.78278	0.50897
N35	Healthy	238	338.625	325.516	3.41344	0.93305	0.62377
N36	Healthy	199	244.769	235.667	3.85596	0.95997	0.72846
N37	Healthy	262	307	298.452	3.903	0.96371	0.70093
N38	Healthy	232	274	259.895	3.57701	0.9108	0.65673
N39	Healthy	259	321.636	308.457	3.85296	0.95499	0.69337
N40	Healthy	232	266.44	264.705	2.7303	0.78738	0.50127
N41	Healthy	267	319.556	305.419	3.75879	0.94395	0.67274
P001	PCOS-LB	220	272.556	266.417	3.59596	0.94438	0.66671
P002	PCOS-HB	144	158.438	162.959	3.14827	0.90752	0.63348
P003	PCOS-HB	175	212.8	198.196	3.46488	0.93477	0.67087
P004	PCOS-HB	123	144.375	137.445	3.11616	0.92977	0.64756
P005	PCOS-HB	202	231.75	237.263	3.87753	0.96697	0.73047
P006	PCOS-LB	280	333.813	316.808	4.19422	0.96909	0.74434
P007	PCOS-LB	147	174	171.09	2.95962	0.90906	0.59306
P008	PCOS-HB	160	181	174.864	3.40261	0.93642	0.67044
P009	PCOS-LB	279	339.273	328.119	4.01318	0.96325	0.71267
P010	PCOS-HB	112	137.5	129.94	3.15523	0.92243	0.66869
P012	PCOS-HB	160	169.5	174.32	3.6094	0.95421	0.71119
P013	PCOS-HB	219	230.5	235.558	3.76963	0.95209	0.69949

P014	PCOS-LB	249	282.3	276.13	3.94373	0.96047	0.71477
P015	PCOS-HB	234	262.636	262.038	3.60213	0.9236	0.6603
P016	PCOS-LB	211	266.714	253.811	3.5321	0.93069	0.65998
P017	PCOS-LB	281	330.583	303.452	4.21612	0.97192	0.74776
P018	PCOS-HB	186	221.429	212.873	3.48877	0.94078	0.66761
P019	PCOS-HB	285	316.316	308.478	4.3338	0.97404	0.76671
P020	PCOS-LB	218	242.231	251.984	3.47048	0.92885	0.64453
P021	PCOS-HB	261	298.625	300.481	3.07221	0.80885	0.55211
P022	PCOS-LB	244	287.043	285.564	3.43272	0.90046	0.62445
P023	PCOS-LB	217	228.55	229.321	3.57957	0.93991	0.66536
P024	PCOS-HB	203	265	230.299	3.7133	0.94935	0.69888
P025	PCOS-HB	117	134.143	128.326	2.90686	0.89278	0.61041
P026	PCOS-HB	90	95.6	93.3039	2.46021	0.82193	0.54674
P027	PCOS-HB	148	183.1	172.03	3.3206	0.92148	0.66449
P028	PCOS-LB	226	269.333	264.635	3.5161	0.9287	0.64866
P030	PCOS-LB	150	285.333	180.047	3.48331	0.94751	0.69518
P031	PCOS-LB	221	228.65	231.082	3.79463	0.94743	0.70295
P033	PCOS-HB	171	199.05	214.557	3.49205	0.93748	0.67917
P034	PCOS-LB	142	155.154	161.581	3.70372	0.96191	0.74735
P035	PCOS-HB	260	324.474	301.25	3.63307	0.94643	0.65335
P036	PCOS-LB	201	246.556	246.246	3.6384	0.94382	0.68606
P037	PCOS-LB	238	279.053	274.013	3.66068	0.94722	0.66895
P038	PCOS-HB	257	305.75	286.232	3.98844	0.96389	0.71876
P039	PCOS-HB	159	181.545	194.511	3.47693	0.94906	0.68593
P040	PCOS-HB	191	202.5	209.204	3.42643	0.92296	0.65237
P041	PCOS-HB	218	264.867	254.083	3.35153	0.90499	0.62244
P042	PCOS-HB	199	232.214	223.666	3.59285	0.94302	0.67875
P043	PCOS-HB	237	255	256.795	3.78015	0.95782	0.69131
P044	PCOS-LB	231	260.077	252.406	4.12256	0.96954	0.75749
P045	PCOS-HB	186	214.05	214.809	3.22538	0.93151	0.61721
P046	PCOS-HB	128	176.462	165.181	1.67143	0.63371	0.34448
P047	PCOS-LB	115	119.875	124.301	3.24672	0.94292	0.68425
P048	PCOS-LB	254	289.455	289.92	3.78457	0.95655	0.68346
P049	PCOS-HB	217	274	252.98	3.86657	0.96395	0.71871
P050	PCOS-LB	75	88	88.0585	1.46007	0.48735	0.33817
P051	PCOS-LB	193	220.556	217.51	2.84172	0.84186	0.53998
P052	PCOS-LB	182	195.125	198.543	3.70323	0.95365	0.71161
P053	PCOS-LB	152	160.75	161.769	3.51649	0.94718	0.69995
P054	PCOS-LB	181	279.333	259.85	2.27498	0.74464	0.43762
P056	PCOS-LB	185	214.176	212.181	3.11051	0.90173	0.59584
P057	PCOS-LB	196	231.2	229.964	3.53952	0.93963	0.6706
P058	PCOS-HB	164	180.867	185.274	3.30322	0.92467	0.64771
P059	PCOS-LB	225	252.75	253.712	3.49552	0.93081	0.64539
P060	PCOS-HB	225	276.476	267.389	3.66109	0.95543	0.67596
P061	PCOS-LB	200	223.4	220.104	3.55175	0.92968	0.67035
P062	PCOS-LB	269	308.808	314.589	3.85895	0.94342	0.68975
P063	PCOS-LB	204	239	239.714	3.65768	0.94609	0.68778
P064	PCOS-LB	247	277.273	276.287	3.83944	0.95828	0.69689
P065	PCOS-HB	91	101.111	106.917	2.83976	0.90538	0.62954
P066	PCOS-LB	182	195.154	193.158	3.73382	0.95871	0.71749
P067	PCOS-HB	137	150.909	148.843	3.16581	0.90785	0.64346
P068	PCOS-LB	198	219.75	227.564	3.37754	0.93051	0.63869

P069	PCOS-LB	205	224.773	229.598	3.61255	0.9528	0.67867
P070	PCOS-LB	173	202.063	206.627	3.27733	0.90524	0.63597
P071	PCOS-HB	238	279.778	285.447	3.67556	0.94665	0.67167
P072	PCOS-HB	223	262.261	260.309	3.64878	0.95199	0.6748
P073	PCOS-HB	134	151.273	153.301	3.05516	0.90835	0.62378
P074	PCOS-HB	214	268.077	245.846	3.9201	0.96741	0.73055
P075	PCOS-LB	214	241.75	249.291	3.55345	0.92308	0.66222
P076	PCOS-HB	191	230	228.458	2.98467	0.88366	0.56826
P077	PCOS-HB	218	263.556	258.578	3.82041	0.9533	0.70952
P078	PCOS-LB	274	342.143	324.43	3.53823	0.93057	0.63035
P079	PCOS-HB	272	305.3	297.589	4.34233	0.97887	0.77461
P080	PCOS-HB	227	303.154	272.465	3.58673	0.93002	0.66116
P081	PCOS-HB	222	255.158	254.354	3.70004	0.94826	0.68485
P082	PCOS-LB	151	159.053	164.69	3.452	0.93924	0.68802
P083	PCOS-HB	195	230.2	218.413	3.08178	0.88026	0.58445
P084	PCOS-LB	178	211.476	215.239	2.63315	0.82607	0.50815
P085	PCOS-LB	177	232.5	213.589	2.98835	0.89868	0.57733
P086	PCOS-LB	238	292.667	276.044	3.60564	0.93759	0.65889
P087	PCOS-LB	157	179.556	185.838	3.51661	0.95388	0.6955
P088	PCOS-HB	206	251.316	249.244	2.85768	0.8334	0.53636
P089	PCOS-LB	199	241	234.648	3.48669	0.92117	0.6587
P090	PCOS-HB	270	309	315.118	3.1066	0.88754	0.55491
P092	PCOS-LB	257	298	291.593	3.42946	0.88995	0.61802
P093	PCOS-LB	192	228.909	216.339	3.59294	0.94782	0.68339
P094	PCOS-LB	176	224.75	215.134	3.17726	0.91738	0.6145
P095	PCOS-LB	159	190.909	180.602	2.47614	0.76531	0.4885
P096	PCOS-HB	184	232.235	227.695	3.10926	0.90271	0.59622
P097	PCOS-HB	197	248.75	258.538	3.21513	0.89446	0.60856
P099	PCOS-LB	236	288.929	267.696	3.81549	0.94972	0.69832
P100	PCOS-HB	188	227	228.648	3.48689	0.94324	0.66589
P101	PCOS-HB	242	281.417	279.479	3.39756	0.92106	0.61898
P102	PCOS-HB	200	224.667	233.699	2.54034	0.78367	0.47946
P103	PCOS-HB	185	233.462	221.625	3.36232	0.92209	0.64408
P104	PCOS-HB	132	157	157.663	3.03712	0.88045	0.622

Supplementary Table 4 Correlation between diversity and clinical parameters

	Observe	Chao1	ACE	Shannon	Simpson	J
TT	-0.15	**-0.19**	**-0.19**	0	0.05	0.06
AD	-0.09	-0.1	-0.12	0.02	0.05	0.06
DHEA-S	-0.09	-0.11	-0.12	0.14	0.17	**0.2**
LH	-0.05	-0.09	-0.08	0.04	0.02	0.07
FSH	-0.14	-0.17	-0.17	0.04	0.05	**0.1**
I0	**-0.23**	**-0.23**	**-0.24**	-0.12	-0.08	-0.06
I120	**-0.22**	**-0.24**	**-0.23**	-0.05	-0.04	0.02
HOMA-IR	**-0.21**	**-0.2**	**-0.22**	-0.12	-0.08	-0.06
IL-22	-0.07	-0.12	-0.12	0.03	0.01	0.06
FKBP5-Met1	0.11	**0.17**	**0.18**	-0.15	-0.14	**-0.22**
FKBP5-Met2	**0.21**	**0.25**	**0.24**	-0.01	-0.04	-0.1
FKBP5-Met	**0.2**	**0.26**	**0.25**	-0.07	-0.09	-0.16

	FKBP5-Met1	FKBP5-Met2	FKBP5-Met
TT	**-0.18**	-0.1	-0.15
AD	-0.17	-0.11	-0.15
DHEA-S	-0.04	0.01	-0.01
LH	**-0.18**	-0.14	**-0.18**
FSH	-0.08	-0.06	-0.08
I0	-0.11	-0.12	-0.14
I120	**-0.21**	**-0.2**	**-0.23**
HOMA-IR	-0.12	-0.1	-0.13
IL-22	-0.11	**-0.19**	**-0.18**

Supplementary Table 5 The relative abundance of bacterial phylum taxa among healthy and PCOS pateints

sample	Actinobac	Bacteroide	Cyanobacteria	Deferribacteres	Epsilonbacteraeota	Euryarchaeota	Firmicutes	Fusobacteria	Patescibacteria
N1	0.00534	0.52059	0	0	0	0	0.41470399	0.000611326	0
N2	0.01248	0.21702	0.000225225	6.43501E-05	0	0.004247104	0.73465251	0	3.2175E-05
N3	0.00557	0.65238	0.000546976	0	0	3.2175E-05	0.3213964	0	0
N4	0.025	0.26393	0	0	0	0	0.69774775	0	0
N5	0.00772	0.51918	0.002574003	0	0	0	0.44671815	0.003088803	3.2175E-05
N6	0.18639	0.2806	6.43501E-05	0	0	0	0.5268018	3.2175E-05	9.65251E-05
N7	0.07761	0.09833	0	0	0	0	0.80717503	0	3.2175E-05
N8	0.00232	0.39266	0	3.2175E-05	0	0	0.58114543	0	6.43501E-05
N10	0.01281	0.27786	9.65251E-05	0	0	0	0.69945302	0	0
N11	0.04633	0.09775	0	0	0	0	0.85357143	0	6.43501E-05
N12	0.00254	0.38977	0	0	0	0	0.56489704	3.2175E-05	9.65251E-05
N14	0.00386	0.56931	0	0	0	0	0.39996782	0	3.2175E-05
N15	0.00151	0.05885	0	0	0	0	0.93227156	0.002413127	0.0002574
N16	0.01065	0.49588	0	0	0	0	0.48426641	0	0
N17	0.00151	0.37719	0	0	0	0	0.59366152	0	3.2175E-05
N18	0.01525	0.54244	9.65251E-05	0	0	0	0.40006435	3.2175E-05	3.2175E-05
N19	0.01071	0.19604	0	0	0	0	0.77889318	0	0
N20	0.01158	0.30302	0	0	0	0	0.67850708	0	3.2175E-05
N21	0.0324	0.36686	0	0	0	0	0.50125483	0	9.65251E-05
N22	0.0029	0.51873	6.43501E-05	0	0	0	0.39005792	0.053603604	0
N23	0.01445	0.4593	0	3.2175E-05	0	0	0.5030888	0.003217503	0
N24	0.00064	0.5353	0	0	0	0	0.38655084	0.014092664	0
N26	0.00354	0.66811	0	0	0	0	0.32351995	3.2175E-05	3.2175E-05
N27	0.00158	0.58858	0	0	0	0	0.35897683	0.001769627	0
N28	0.0092	0.23536	0	0	0	0	0.66808237	0.072361647	6.43501E-05
N29	0.00833	0.69839	0	0	0	0	0.27541828	3.2175E-05	0
N30	0.00727	0.30167	0	0	0	0	0.521139	0.026608752	0
N31	0.04524	0.26422	0	0	0	0	0.65324968	0.001287001	3.2175E-05
N32	0.09591	0.34801	0	0	0	0	0.53458816	0	0
N33	0.16351	0.20718	0	0	0	0	0.62149292	0.000160875	0.000160875
N34	0.01133	0.62407	0	3.2175E-05	0	0	0.34546332	9.65251E-05	0
N35	0.02815	0.34704	3.2175E-05	6.43501E-05	0	0	0.55	0.037162162	9.65251E-05

N36	0.03771	0.34447	0	0	0	0	0.60534106	0.000772201	6.43501E-05
N37	0.0177	0.22696	0	0	0	0.003539254	0.73519949	0	6.43501E-05
N38	0.00035	0.47159	0	0	0	0	0.51544402	0	3.2175E-05
N39	0.02272	0.21744	0	0	0	0	0.7287323	0	0
N40	0.00209	0.63787	0	0	0	0	0.34305019	0	0
N41	0.00672	0.36406	3.2175E-05	0	0	0	0.58072716	0	3.2175E-05
P001	0.00309	0.45103	3.2175E-05	0	0	0	0.51332046	0	6.43501E-05
P002	0.00283	0.42455	0	0	0	0	0.53458816	0.005501931	0
P003	0.01145	0.38105	3.2175E-05	0	0	0	0.56753539	0	0
P004	0.01306	0.10682	3.2175E-05	0	0	0	0.75350708	0.122651223	0
P005	0.00708	0.31577	0.000579151	0	9.65251E-05	0	0.60382883	0.007110682	0.00019305
P006	0.02239	0.2075	6.43501E-05	0	3.2175E-05	0.001222651	0.7505148	0	0
P007	0.00322	0.39607	3.2175E-05	0	0	0	0.58490991	0.000675676	0.00019305
P008	0.01213	0.25328	0	0	3.2175E-05	0	0.72059202	0	6.43501E-05
P009	0.01033	0.34923	0	0	0.000225225	0	0.57702703	3.2175E-05	0
P010	0.01213	0.50952	0	0	0	0	0.3041184	0.108462033	6.43501E-05
P012	0.01364	0.32619	0	0	0	0	0.63513514	0	0
P013	0.0056	0.37709	0	0	0	0	0.56882239	0	6.43501E-05
P014	0.00859	0.40676	3.2175E-05	0	0	0	0.56969112	0	0
P015	0.00524	0.44176	6.43501E-05	0	0	0	0.51737452	0	3.2175E-05
P016	0.00692	0.38941	0	0	0	0	0.57477477	0	3.2175E-05
P017	0.0037	0.4083	0	0	0	0.000160875	0.5472973	0	0
P018	0.01779	0.45923	0	0	0	0	0.4543758	0.060682111	0
P019	0.00309	0.19772	3.2175E-05	0	0	0	0.75833333	0	0
P020	0.01757	0.50502	0	0	0	0	0.39720077	0	6.43501E-05
P021	0.00106	0.58523	0	0	0	0	0.39498069	0.000289575	3.2175E-05
P022	0.00505	0.48784	0	0	0	0	0.48564994	0	6.43501E-05
P023	0.00241	0.49279	0	0	0	0.000225225	0.48346203	0	0
P024	0.01281	0.39672	0.0001287	0	0	0	0.5492278	0	0
P025	0.00907	0.66963	0	3.2175E-05	0	0	0.24620335	0	3.2175E-05
P026	0.00048	0.7249	0	0	0	0	0.20453668	0	0
P027	0.00071	0.37387	0	0	0	0	0.54208494	0.021364221	0.000160875
P028	0.00418	0.54813	0	0	0	0	0.42027027	3.2175E-05	0
P030	0.02165	0.26094	0	0	0	0	0.62181467	0	0

P031	0.01313	0.40351	0	0	0	0	0.56772844	0	0
P033	0.04205	0.19408	0	0.033815959	0	0	0.7014157	0	0.025289575
P034	0.03623	0.55061	0	0.036583012	0	0	0.30363578	0	0.012934363
P035	0.00122	0.36284	0	0	0	0	0.57245817	0.004761905	3.2175E-05
P036	0.02249	0.2722	0	0	0	0	0.64037967	9.65251E-05	9.65251E-05
P037	0.00257	0.55039	3.2175E-05	0	0	0.000353925	0.42712355	0	3.2175E-05
P038	0.01387	0.25325	0	0	0	0	0.66875804	0.007689833	0
P039	0.00161	0.17523	0	0	0	0	0.7527027	0.00045045	3.2175E-05
P040	0.00097	0.61168	3.2175E-05	0	0	0	0.35418275	0	3.2175E-05
P041	0.01271	0.386	0	0	0	0	0.57226512	3.2175E-05	3.2175E-05
P042	0.02246	0.29916	0	0	0	0	0.6256435	0	3.2175E-05
P043	0.00679	0.49607	0	0	0	0	0.46747104	3.2175E-05	3.2175E-05
P044	0.00785	0.29041	9.65251E-05	0	0	0	0.63304376	0.010199485	0
P045	0.00199	0.37262	0	0	0	0	0.34259974	0.207754183	3.2175E-05
P046	0.00808	0.27178	3.2175E-05	0	0	0	0.11483269	0.006531532	0.000225225
P047	0.01873	0.13687	0	0	0	0	0.70453668	0.002059202	6.43501E-05
P048	0.00827	0.48279	0	0	0	0	0.43667954	0.030083655	0
P049	0.01104	0.43475	0	0	0	0	0.50942728	0.00019305	0
P050	0.00045	0.74295	0	0	0	0	0.19430502	0.053507079	3.2175E-05
P051	0.0009	0.19685	0	0	0	0	0.74083012	0	6.43501E-05
P052	0.00653	0.47014	0	0	0	0	0.48381596	0.00019305	0
P053	0.00537	0.55415	0	0	0	0	0.37715573	0.026126126	6.43501E-05
P054	0.01068	0.18658	0.004375804	0	0	0	0.68388031	0	0.000514801
P056	0.00692	0.65586	0	0	0	0	0.28841699	0.004761905	0.000160875
P057	0.00061	0.57886	0	0	0	0	0.39800515	0	9.65251E-05
P058	0.00647	0.3472	0	0	0	0	0.6287323	0	0
P059	0.00447	0.54633	0	3.2175E-05	0	0	0.42184685	0	6.43501E-05
P060	0.01216	0.57005	0	3.2175E-05	0	0	0.35212355	0.000514801	0
P061	0.00647	0.2916	0	0	0	0	0.6759009	6.43501E-05	0.000160875
P062	0.00306	0.20721	3.2175E-05	0	0	0	0.7778314	0	3.2175E-05
P063	0.04614	0.24649	0	0	0	0	0.6962677	0.000353925	3.2175E-05
P064	0.02153	0.23851	0	0	0	0	0.71283784	0	0
P065	0.06795	0.36001	0	0	0	0	0.45630631	0.000740026	0.0001287
P066	0.00148	0.25183	0	0	0	0	0.69527027	0.005823681	0.000160875

P067	0.00122	0.43108	0	0	0	0	0.51940154	3.2175E-05	9.65251E-05
P068	0.00251	0.50273	0	0	0	0	0.4756435	0	0
P069	0.02754	0.17156	0	0	0	0	0.79108752	0	0
P070	0.04131	0.36818	0	0	0	0	0.53407336	0.000868726	3.2175E-05
P071	0.06998	0.29112	0	0	0	0	0.62393822	0.000836551	0.0003861
P072	0.06123	0.05653	3.2175E-05	0	0	0	0.77853925	0	9.65251E-05
P073	0.00322	0.36432	0	0	0	0	0.60125483	0	6.43501E-05
P074	0.01142	0.26313	0	0	0	0	0.70820463	0	0
P075	0.00431	0.47069	0	0	0	0	0.50633848	0.011196911	3.2175E-05
P076	0.0064	0.40203	0	0	0	0	0.51515444	0	0
P077	0.00421	0.3008	0	3.2175E-05	0	0	0.67326255	3.2175E-05	3.2175E-05
P078	0.02423	0.27346	0	0	0	0	0.62432432	0.001383526	3.2175E-05
P079	0.02931	0.18967	0	0	0	0.001640927	0.74745817	0	3.2175E-05
P080	0.00428	0.33246	0	0	0	0	0.62828185	0	0
P081	0.00412	0.30438	3.2175E-05	0	0	0	0.67815315	0	0
P082	0.02841	0.16927	0	0	0	0	0.7759009	9.65251E-05	9.65251E-05
P083	0.00241	0.61181	0	0	0	0	0.36875804	0	0
P084	0.00701	0.70833	0.000418275	0	0	0	0.25312098	0.002155727	3.2175E-05
P085	0.01046	0.42046	0	0	3.2175E-05	0	0.48294723	0.023391248	0
P086	0.00122	0.42127	0	0	0	0	0.54057272	0.000707851	3.2175E-05
P087	0.17046	0.07574	0	0	0	0	0.73092021	9.65251E-05	0.000160875
P088	0.05019	0.64453	0	0	0	0	0.29147362	0	0
P089	0.00354	0.25148	0	0	0	0	0.69720077	6.43501E-05	6.43501E-05
P090	0.0129	0.52593	0	0	0	0.0001287	0.42876448	0.000353925	3.2175E-05
P092	0.00219	0.5323	0	0	0	0	0.42274775	0.003410553	6.43501E-05
P093	0.03169	0.42481	0	0	0	0	0.50707851	0.00019305	0
P094	0.10138	0.30608	0	0	0	0	0.54227799	0.007561133	9.65251E-05
P095	0.00389	0.65183	0	0	0	0	0.30952381	0	3.2175E-05
P096	0.00589	0.38687	0	0	0	0	0.59272844	3.2175E-05	6.43501E-05
P097	0.00148	0.57436	0	0	0	0	0.40267053	0	0
P099	0.00331	0.27342	0	0	0	0	0.70987773	0	0
P100	0.03134	0.35222	0	0	0	0	0.56386744	0.005791506	0.00045045
P101	0.00109	0.53201	0.000160875	0	0	0	0.43291506	0.000804376	0.0001287
P102	0.00203	0.76641	0	0	0	0	0.22039897	3.2175E-05	0

P103	0.01133	0.475	0	0	0	0	0.47561133	0	0
P104	0.02944	0.51464	6.43501E-05	0	0	0	0.4018018	3.2175E-05	9.65251E-05

Proteobacteria	Synergistetes	Tenericutes	Verrucomicrobia
0.058236808	0.000514801	0	0
0.028153153	0.000900901	0	0.002220077
0.02007722	0	0	0
0.013288288	0	0	3.2175E-05
0.020688546	0	0	0
0.006016731	0	0	0
0.016859717	0	0	0
0.023230373	0.000546976	0	0
0.00978121	0	0	0
0.001962677	3.2175E-05	0	0.000289575
0.042631918	0	0	3.2175E-05
0.026769627	6.43501E-05	0	0
0.004697555	0	0	0
0.009202059	0	0	0
0.027606178	0	0	0
0.042084942	0	0	0
0.014350064	0	0	0
0.006756757	0	0	9.65251E-05
0.09520592	0.003635779	0	0.000546976
0.033494208	0.001158301	0	0
0.019916345	0	0	0
0.063416988	0	0	0
0.004761905	0	0	0
0.049099099	0	0	0
0.014864865	0	0	6.43501E-05
0.017792793	0	0	3.2175E-05
0.143307593	0	0	0
0.035971686	0	0	0
0.021492921	0	0	0
0.007464607	0	0	3.2175E-05
0.019015444	0	0	0
0.037451737	0	0	0

0.011615187	0	0	3.2175E-05
0.015765766	0.000643501	0	0.0001287
0.012194337	0	6.43501E-05	0.00032175
0.031081081	0	0	3.2175E-05
0.016505792	9.65251E-05	0	0.0003861
0.048391248	0	0	3.2175E-05
0.032464607	0	0	0
0.032496782	0	0	3.2175E-05
0.03957529	0.000353925	0	0
0.003925354	0	0	0
0.06534749	0	0	0
0.017567568	0.000707851	0	0
0.01489704	0	0	0
0.013899614	0	0	0
0.062129987	9.65251E-05	0.000933076	0
0.065701416	0	0	0
0.025032175	0	0	0
0.047619048	0	0.000804376	0
0.014929215	0	0	0
0.035521236	0	0	0
0.028861004	0	0	0
0.04041184	9.65251E-05	3.2175E-05	0
0.007915058	0	0	0
0.040572716	0.0002574	0	0
0.07992278	0.000160875	0	6.43501E-05
0.018275418	9.65251E-05	3.2175E-05	0
0.021267696	0.0001287	0	0
0.020817246	0.000160875	9.65251E-05	3.2175E-05
0.041119691	0	0	0
0.075032175	0	0	0
0.07007722	0	0	0
0.061808237	0	0	0
0.027380952	0	0	0
0.095592021	0	0	0

0.015637066	0	0	0
0.002574003	0	0	0.000772201
0.005759331	0	0	0.054247104
0.058622909	3.2175E-05	0	3.2175E-05
0.063577864	0.001158301	0	0
0.018951094	0.000546976	0	0
0.056145431	0.000289575	0	0
0.069980695	0	0	0
0.033108108	0	0	0
0.028925354	3.2175E-05	0	0
0.052670528	3.2175E-05	0	0
0.029568855	0	3.2175E-05	0
0.052059202	0.006338481	0	0
0.074967825	3.2175E-05	0	0
0.598519949	0	0	0
0.137741313	0	0	0
0.042181467	0	0	0
0.044594595	0	0	0
0.008751609	0	0	0
0.061357786	0	0	0
0.029021879	0	0	0.01029601
0.037129987	0	0	0
0.113931789	3.2175E-05	0	0
0.043886744	0	0	0
0.022232947	0.00019305	0	0
0.017599743	0	0	0
0.023584299	0	0	0.003667954
0.065122265	0	0	0
0.025804376	0	0	0
0.011808237	3.2175E-05	0	0
0.010682111	3.2175E-05	0	0
0.027059202	0	0	6.43501E-05
0.114864865	0	0	0
0.045431145	0	0	0

0.047779923	0.0003861	0	0
0.016698842	0	0	0.002413127
0.00965251	0	0.000160875	0
0.05511583	0	0	0.000418275
0.012580438	3.2175E-05	0.000997426	0.0001287
0.103507079	3.2175E-05	0	3.2175E-05
0.031145431	0	0	0
0.017052767	0.00019305	0	0
0.007432432	0	0	0
0.076319176	9.65251E-05	0	0
0.021203346	0.00019305	0	0.000225225
0.076544402	3.2175E-05	0	0
0.026061776	0.000418275	0	0.005405405
0.034877735	0	0	9.65251E-05
0.012998713	0	0.00032175	0
0.026158301	6.43501E-05	0	0
0.016988417	3.2175E-05	0	0
0.028925354	0	0	0
0.062709138	0	0	0
0.035939511	0.000225225	3.2175E-05	0
0.022619048	0	0	0
0.013642214	0.0001287	0	3.2175E-05
0.047651223	0	0	0
0.031853282	0	0	3.2175E-05
0.039189189	9.65251E-05	0	0
0.036100386	3.2175E-05	0	9.65251E-05
0.042599743	0	0	0
0.03471686	0	0	0
0.014382239	0	0	3.2175E-05
0.021492921	0	0	0
0.012902188	0	0	0.000482625
0.046299871	0	0	3.2175E-05
0.032850708	3.2175E-05	0	0
0.011100386	0	0	3.2175E-05

0.038030888	0	0	3.2175E-05
0.053925354	0	0	0

Supplementary Table 6 The relative abundance of top bacterial genus taxa among healthy and PCOS pateints

sample	Bacteroide	Faecalibact	Prevotella_	Roseburia	un_f_Lach	Blautia	Megamona	Lachnoclos	Lachnospir	Subdoligra	Prevotella_	Escherichia	Ruminococ
N1	0.479858	0.097619	0	0.092181	0.030373	0.009266	0	0.012516	0.013514	0.006821	0	0.028411	0.019916
N2	0.146622	0.076094	3.22E-05	0.026802	0.097941	0.010264	0	0.01036	0.014125	0.028958	6.44E-05	0.002252	0.016602
N3	0.02101	0.066152	0.520978	0.066216	0.023938	0.010103	0	0.008076	0.002574	0.002928	0.069015	0.002091	0.001866
N4	0.232432	0.192149	0.000193	0.173488	0.049196	0.049968	0.022201	0.006853	0.016795	0.030277	0	0.001577	0.007979
N5	0.514511	0.088417	6.44E-05	0.072716	0.070721	0.052156	3.22E-05	0.009202	0.012484	0.005277	0	0.00666	0.001416
N6	0.275225	0.045335	3.22E-05	0.138771	0.046236	0.065605	3.22E-05	0.050354	0.016216	0.000129	3.22E-05	0.004762	0.017503
N7	0.083366	0.205598	0.002284	0.090347	0.073938	0.072233	0.000322	0.002606	0.005985	0.058269	9.65E-05	0.005952	0.01583
N8	0.036165	0.123327	0.262548	0.057625	0.029955	0.012516	0.026158	0.008977	0.004215	0.014704	0.018565	0.002188	0.002349
N10	0.102542	0.083301	9.65E-05	0.187902	0.033398	0.035071	0.08462	0.009846	0.052606	0.014093	0.158945	3.22E-05	0.0139
N11	0.055245	0.052091	0.011744	0.032014	0.067728	0.097072	0.010843	0.000386	0.011454	0.053121	0	0.00029	0.012773
N12	0.380084	0.137645	6.44E-05	0.049356	0.156306	0.032754	0.034427	0.027349	0.016731	0.0037	0	0.025161	0.016023
N14	0.231918	0.069852	0.318468	0.007304	0.024614	0.008494	0.149517	0.05843	0.000869	0.010489	0	0.00769	0.002896
N15	0.043919	0.235328	0.000193	0.044562	0.118275	0.035039	0.00074	0.005824	0.00296	0.103185	0.003443	0.002252	0.009524
N16	0.028314	0.195753	0.288031	0.037934	0.032272	0.013449	0	0.013803	0.01464	0.012709	0.090444	0.000547	0.003604
N17	0.350515	0.108752	0.000772	0.102864	0.055212	0.014607	0.000322	0.012838	0.003829	0.057432	0.000772	0.00074	0.040541
N18	0.011712	0.066248	0.462291	0.040058	0.037484	0.007947	0.126737	0.0176	0.02899	0.007722	0.020592	9.65E-05	0.00251
N19	0.170753	0.248166	3.22E-05	0.052671	0.033237	0.034427	0.021107	0.006435	0.005438	0.03668	0	0.000418	0.006982
N20	0.294112	0.086486	0.000161	0.030727	0.068404	0.035586	0	0.00843	0.033526	0.099807	0	0.000547	0.028958
N21	0.324839	0.116441	0	0.013514	0.079344	0.024743	0	0.016474	0.020721	0.022748	6.44E-05	0.00769	0.018951
N22	0.387066	0.090959	9.65E-05	0.026062	0.008848	0.016345	0.038127	0.009395	0.032754	0.000515	0.117696	0.006017	0.006178
N23	0.454826	0.219884	0.000161	0.076351	0.027767	0.025161	0	0.015734	0.002059	0.025772	0	0.000483	0.012934
N24	0.520882	0.077284	0.00074	0.031532	0.058366	0.024453	3.22E-05	0.033816	0	0.002156	3.22E-05	0.005792	0.038546
N26	0.042503	0.084813	0.611068	0.055631	0.017214	0.009009	0.014093	0.007046	0.008848	0.006371	0	0.000547	0.002606
N27	0.527864	0.000161	0.000965	0.02545	0.071718	0.019466	0	0.066313	0.011229	0.005985	0	0.002284	0.034781
N28	0.188835	0.12455	0.002188	0.085811	0.134395	0.078861	6.44E-05	0.036197	0.013642	0.025161	0	6.44E-05	0.019852
N29	0.069337	0.050225	0.596686	0.025515	0.019627	0.00991	0.041988	0.013353	0.005309	0.017986	0.000322	0.000257	0.011197
N30	0.270817	0.179665	0	0.149485	0.032883	0.015412	9.65E-05	0.014994	0.03536	0.000418	0.000354	0.012613	0.010039
N31	0.079762	0.122362	0.16908	0.067117	0.066892	0.031435	0.028378	0.015573	0.069048	0.013127	0	0.007079	0.016377
N32	0.331982	0.134588	0.000225	0.032851	0.019788	0.049517	0.076287	0.008977	0.017728	0.006178	0	0.008333	0.018275
N33	0.154376	0.083172	0.01316	0.013127	0.0287	0.13594	3.22E-05	0.006403	0.001737	0.029215	3.22E-05	0.001126	0.012098
N34	0.115283	0.073584	0.505341	0.036229	0.054665	0.015122	3.22E-05	0.02204	0.002156	0.013674	0.000193	0.001512	0.007304
N35	0.330631	0.041892	0.001126	0.112902	0.071525	0.027124	6.44E-05	0.025257	0.033655	0.000515	3.22E-05	0.000354	0.000515
N36	0.31258	0.117696	3.22E-05	0.017375	0.06657	0.027124	3.22E-05	0.022844	0.035039	0.067857	0	0.002574	0.010071
N37	0.194273	0.086358	6.44E-05	0.085296	0.07722	0.084556	0.028925	0.009331	0.004923	0.065766	3.22E-05	0.004569	0.0139
N38	0.139736	0.062033	0.288063	0.07471	0.043147	0.034073	3.22E-05	0.012999	0.01242	0.009556	0.037516	9.65E-05	0.01583
N39	0.193115	0.140347	0.001094	0.144691	0.050901	0.054569	0	0.00769	0.008333	0.017439	9.65E-05	0.000804	0.013353

N40	0.105438	0.066474	0.514414	0.067149	0.026931	0.010553	0.016409	0.007851	0.004472	0.02426	0	0.008269	0.001898
N41	0.326416	0.148166	0.008269	0.073263	0.086422	0.019916	0.000161	0.023102	0.016055	0.010521	0	0.00029	0.010006
P001	0.41509	0.109556	0	0.091216	0.091763	0.015058	0	0.029279	0.022072	0.000676	0	9.65E-05	0.008269
P002	0.415154	0.040283	0	0.052091	0.042921	0.023069	0	0.092246	0.021107	0.010618	0	0.021686	0.023166
P003	0.341538	0.088063	0	0.196364	0.016184	0.015959	0	0.035457	0.022748	0.008398	0	0.013031	0.007883
P004	0.103443	0.139897	0	0.026448	0.065862	0.027671	0	0.01213	0	3.22E-05	0	0.002091	0.022716
P005	0.208205	0.082625	0.071589	0.046654	0.068115	0.02056	0	0.076287	0.050837	0.003443	0	0.026416	0.03565
P006	0.140026	0.114093	0	0.027252	0.044337	0.049421	0	0.006371	0.047651	0.039736	0.011165	0.000933	0.012677
P007	0.115058	0.011068	0.000193	0.086486	0.176673	0.008366	0.155373	0.013417	0.022555	0.000161	0.279344	0.001931	0.041023
P008	0.253282	0.14601	0	0.126995	0.148069	0.035746	0	0.008752	0.018951	0.00029	0	0.001158	0.012838
P009	0.322233	0.094015	0	0.03639	0.05917	0.01509	0	0.023391	0.010586	0.02545	0	0.014414	0.021686
P010	0.490508	0.050901	0	0.026834	0.057529	0.007979	0	0.01686	0.002896	0.000418	0	0.004054	0.04157
P012	0.263578	0.121976	0	0.044208	0.055051	0.118758	0	0.01657	0.02899	0.000515	0	6.44E-05	0.025322
P013	0.318533	0.052252	0	0.139897	0.057465	0.022104	0.01435	0.007786	0.05045	0.016023	0	0.015412	0.005985
P014	0.343082	0.061229	0	0.043179	0.050997	0.03742	0	0.002864	0.016441	0.020045	0	0.002124	0.018533
P015	0.398456	0.108108	0	0.038417	0.064414	0.009781	0	0.013932	0.037999	0.007722	0	0.001641	0.026995
P016	0.127477	0.176963	0.236068	0.072587	0.071976	0.022136	0	0.017246	0.019337	0.014607	0	0.00518	0.022716
P017	0.084813	0.084234	0.138192	0.086229	0.064865	0.025933	0	0.007336	0.002992	0.010875	0.095238	0.002638	0.005631
P018	0.426384	0.040959	0	0.023198	0.0426	0.021139	0.106274	0.029086	0.004183	0.013127	0	0.001255	0.021171
P019	0.116441	0.091377	0	0.057819	0.069562	0.024131	3.22E-05	0.011551	0.023069	0.020109	0	0.000708	0.017181
P020	0.453443	0.05769	0	0.07249	0.028636	0.019015	0	0.021171	0.005212	0.009299	0	0.008205	0.036486
P021	0.077188	0.06332	0.471107	0.043662	0.047265	0.012033	0.017471	0.013256	0.002735	0.030277	0.012613	6.44E-05	0.006918
P022	0.43536	0.089768	0	0.094562	0.02529	0.015444	0	0.011873	0.040122	0.010586	0	3.22E-05	0.005952
P023	0.417825	0.044273	0	0.018565	0.017696	0.024099	0.000611	0.011583	0.010039	0.019048	0	0.000193	0.013932
P024	0.212194	0.138514	0.166795	0.072458	0.075869	0.027864	0.021107	0.016023	0.027606	0.014157	0	0.018533	0.018436
P025	0.256017	0.067342	0.40695	0.007722	0.01287	0.023874	0	0.011744	0.026158	0.00045	0	0.014447	0.006596
P026	0.719112	0.001223	0	0.000515	0.042793	0.010907	0	0.034073	0	9.65E-05	0	0.056853	0.001737
P027	0.371782	0.010457	0	0.033977	0.157819	0.041602	0	0.039028	0.038063	0.000257	0	0.028218	0.00592
P028	0.471364	0.084685	0	0.01435	0.063031	0.018597	0	0.012452	0.033559	0.042696	0	0.000322	0.013578
P030	0.254569	0.092921	0	0.168082	0.067149	0.017921	0	0.034106	0.048423	0.001416	0	0.002284	0.034041
P031	0.145206	0.13964	0.204923	0.064221	0.03713	0.018726	0	0.005534	0.011293	0.009106	0	0.001512	0.020463
P033	0.011873	0.000257	0.000257	0.00029	0.162967	0.000676	3.22E-05	0.022362	0.000129	6.44E-05	0	0.00103	0
P034	0.016634	0	0	0.000129	0.061647	0.000193	0	0.012323	0	0	0	0	0
P035	0.317825	0.09334	0	0.154022	0.046139	0.007819	0.069659	0.008687	0.001609	0.010296	0	0.052896	0.007593
P036	0.259685	0.127831	0	0.0639	0.069144	0.018468	0.059073	0.017728	0.018629	0.022233	0	0.053475	0.012967
P037	0.510586	0.060489	0	0.084138	0.014607	0.015991	0.003346	0.013256	0.011551	0.005631	0	0	0.008494
P038	0.222362	0.065315	0	0.066763	0.040026	0.024775	0.119788	0.013353	0.024228	0.010167	0	0.010393	0.005309
P039	0.172587	0.135843	0.000129	0.054601	0.109073	0.118115	0	0.009331	0.042214	0.002703	3.22E-05	0.016281	0.012291

P040	0.585135	0.047587	0	0.062452	0.047233	0.023005	0.011133	0.025032	0.016538	0.004344	0	0.010811	0.013031
P041	0.099003	0.144562	0	0.096107	0.045785	0.00962	0.011004	0.010618	0.029601	0.025644	0.250354	0.001705	0.023295
P042	0.280277	0.125804	0	0.059202	0.079311	0.019723	0	0.022523	0.040026	0.017471	0	0.007336	0.034202
P043	0.244144	0.03742	0	0.09971	0.028732	0.007175	0	0.027188	0.010843	0.01139	0.126963	0.002413	0.006692
P044	0.23816	0.011905	0	0.041634	0.062967	0.017149	0.020753	0.026866	0.032947	0.042535	3.22E-05	0.008687	0.093179
P045	0.179569	0.007432	0.187066	0.006853	0.033591	0.004344	0.101802	0.043822	0.010039	0.003443	0	0.058076	0.009266
P046	0.261712	0.000193	0	9.65E-05	0.002671	0.00119	0.08639	0.00103	0	0.000483	0	0.578411	0.000515
P047	0.136551	0	0	0.147265	0.090283	0.067632	0	0.013964	0.028218	0	0	0.043243	0.009459
P048	0.422362	0.067889	0	0.048584	0.045753	0.006564	0.014736	0.008172	0.003764	0.01361	0	0.024099	0.039833
P049	0.269916	0.076866	0	0.012259	0.041956	0.038674	0	0.019241	0.00769	0.040894	0	0.00222	0.006789
P050	0.734717	0	0	0.003636	0.012709	0.01657	0	0.012227	0.001609	0.000354	0	0.008044	0.030952
P051	0.171782	0.029762	0	0.104633	0.020109	0.005888	0	0.008333	0.078314	0.002477	0	0.032722	0.003121
P052	0.253797	0.140766	0.11036	0.017246	0.03861	0.00518	0.074421	0.03935	0.053764	0.001384	0	0.000354	0.004022
P053	0.474839	0	0	0.048295	0.028153	0.023359	0	0.037645	0.014929	0.032625	0	0.0111	0.025804
P054	0.026351	0.015991	0.09601	0.000676	0.037194	0.003378	9.65E-05	0.043082	0.000129	0.00045	0.061293	0.102284	0.000257
P056	0.552831	0.030888	0	0.060425	0.02056	0.005566	0	0.03594	0.002188	0.000386	0.059106	0.000161	0.002252
P057	0.530534	0.039093	0	0.09926	0.024003	0.01509	0	0.013674	0.012291	0.010714	0	0.002027	0.03536
P058	0.330502	0.194949	0	0.067889	0.09186	0.009363	9.65E-05	0.017407	0.027252	0.03269	0	0	0.006596
P059	0.458076	0.019852	0	0.109653	0.019852	0.002317	0.032207	0.019595	0.021107	0.024196	0	0.002124	0.008848
P060	0.393951	0.048327	0	0.067568	0.054151	0.007304	6.44E-05	0.027671	0.024356	0.003185	0.123391	0.003958	0.009138
P061	0.264189	0.228539	0	0.061358	0.064961	0.035296	0.053089	0.003378	0.017728	0.027831	0	0.004665	0.02278
P062	0.11731	0.192181	0.029118	0.123552	0.066795	0.05917	0	0.010521	0.006853	0.016506	0.003539	0	0.012484
P063	0.234749	0.177542	0	0.075386	0.090283	0.067632	0	0.007658	0.038867	0.00769	6.44E-05	0.0074	0.014414
P064	0.155727	0.131918	0	0.068308	0.064768	0.012709	0	0.01094	0.019048	0.035071	0.064157	3.22E-05	0.007883
P065	0.358977	9.65E-05	0	0.121815	0.001384	0.101737	0	0.063642	0.023423	0	0	0.056274	0.000129
P066	0.201737	0.036583	0.021332	0.105148	0.116699	0.080277	0.021589	0.031403	0.028893	0.007915	0	0.020463	0.019788
P067	0.42397	0.200515	3.22E-05	0.058462	0.03861	0.017407	0	0.032304	0.032014	0.000354	3.22E-05	0.008494	0.001673
P068	0.470753	0.108623	9.65E-05	0.128732	0.020753	0.012645	0	0.006757	0.009685	0.006918	0	0.003089	0.017889
P069	0.127284	0.106692	3.22E-05	0.11406	0.096171	0.08964	6.44E-05	0.000644	0.009299	0.043147	0	0.000933	0.009781
P070	0.359138	0.13906	6.44E-05	0.040476	0.060328	0.02751	0	0.016795	0.02397	9.65E-05	0	0.007883	0.001351
P071	0.222523	0.147104	3.22E-05	0.020045	0.072748	0.034653	3.22E-05	0.000869	0.002349	0.062613	0	0.002992	0.006725
P072	0.041956	0.137741	3.22E-05	0.115219	0.064479	0.088449	3.22E-05	0.015734	0.044305	0.016377	3.22E-05	0.001319	0.000225
P073	0.356853	0.171943	3.22E-05	0.018983	0.042375	0.016345	0.069402	0.050611	0.035264	0.00045	0	0.006178	0.002542
P074	0.213353	0.048359	0.000129	0.120849	0.092696	0.087387	3.22E-05	0.033784	0.035264	0.018694	0	0.002381	0.009266
P075	0.450837	0.102864	3.22E-05	0.03121	0.046396	0.044176	3.22E-05	0.004376	0.015605	0.026351	0	0.001448	0.024389
P076	0.033301	0.06908	0.308784	0.008848	0.025644	0.008076	0.020689	0.031049	0.000386	0.020753	0.04379	0.03388	0.007786
P077	0.159524	0.159106	0.099743	0.11332	0.040927	0.019627	9.65E-05	0.012484	0.015766	0.025064	0	0.003507	0.019981
P078	0.20666	0.167761	0.039447	0.01538	0.057143	0.017439	0.001866	0.00769	0.0037	0.02056	0	0.065573	0.008398

P079	0.097651	0.037194	6.44E-05	0.018082	0.046911	0.018179	0	0.010425	0.007207	0.075644	0	0.005695	0.029022
P080	0.306338	0.204858	6.44E-05	0.039897	0.106242	0.040026	3.22E-05	0.006885	0.006435	0.011615	0	0.000354	0.010875
P081	0.282658	0.16657	3.22E-05	0.075	0.045528	0.022265	0.060714	0.013642	0.025515	0.028346	0	0.000129	0.008816
P082	0.164479	0.178089	3.22E-05	0.106371	0.061937	0.047104	0	0.022716	0.089704	0.00029	0	0.005792	0.024646
P083	0.09881	0.05	0.436133	0.03832	0.025611	0.005019	0.078507	0.029311	0.035071	0.002317	0.070463	0.00843	0.018694
P084	0.02352	0.053346	0.53121	0.027799	0.038771	0.005084	6.44E-05	0.007979	0.006596	3.22E-05	0.144466	0.002156	0.005084
P085	0.376512	0.05399	0	0.055824	0.040154	0.011776	0.104054	0.014672	0.027059	0.000129	0.038224	0.059459	0.006885
P086	0.137838	0.078153	0.252574	0.186004	0.048777	0.009524	3.22E-05	0.020109	0.008172	0.016795	0	0.008784	0.007979
P087	0.075386	0.07796	0.000225	0.064865	0.086615	0.053314	3.22E-05	0.007979	0.028861	0.013867	0	0.008977	0.003121
P088	0.064897	0.070045	0.525257	0.041248	0.017825	0.011519	0.000515	0.004505	0.00045	0.017214	6.44E-05	0.001737	0.002542
P089	0.21509	0.229054	9.65E-05	0.040669	0.042085	0.041699	0	0.019949	0.021042	0.027445	0	0.017535	0.006178
P090	0.021075	0.16332	0.435779	0.045174	0.028829	0.008205	0.007786	0.003346	0.009588	0.010296	0.053571	0.010586	0.005212
P092	0.159202	0.088256	0.31406	0.050129	0.035071	0.01213	9.65E-05	0.008398	0.020721	0.009202	0.041377	0.00029	0.003057
P093	0.40177	0.079376	0.000193	0.084813	0.038835	0.020174	0.082851	0.028153	0.001255	0.0037	6.44E-05	0.005759	0.007625
P094	0.070817	0.101705	0.233237	0.045592	0.023359	0.033108	0.172683	0.017246	0.020624	0.002317	0.000483	0.002703	0.004247
P095	0.016602	0.074839	0.600772	0.026126	0.015347	0.029633	0	0.005277	0.000161	0.003185	0.022072	0.011712	0.006403
P096	0.229279	0.024743	0.151416	0.037741	0.068404	0.00888	0.256564	0.030695	0.001898	0.001866	0	0.004215	0.009878
P097	0.144048	0.098102	0.408687	0.042439	0.032722	0.019402	0	0.041538	6.44E-05	0.01036	0	0.004601	0.018115
P099	0.233172	0.13343	0.000129	0.133655	0.038127	0.023391	0	0.007658	0.008076	0.014672	0	0.002896	0.013256
P100	0.32677	0.049421	0.00074	0.021493	0.03491	0.038127	0.163739	0.052831	0.075676	0.002381	3.22E-05	6.44E-05	0.011551
P101	0.46361	0.118629	0.006467	0.023713	0.038707	0.01406	0.038385	0.036615	0.017085	0.005598	0.000515	0.003475	0.014961
P102	0.026416	0.072329	0.658526	0.017728	0.024292	0.007465	0.029794	0.006596	0.001802	0.003571	0.076126	0.002799	0.001158
P103	0.453346	0.024131	0.007915	0.050483	0.074324	0.045528	6.44E-05	0.047169	0.090315	0.001673	0.000547	0.018629	0.013481
P104	0.502091	0.02455	6.44E-05	0.030212	0.031692	0.027928	0	0.059781	0.03861	0.000161	3.22E-05	0.034685	0.014575

Ruminococ	Bifidobacte	Alistipes	Phascolarc	Eubacteriu	Anaerostip	Others
0.02471	0.003829	0.021461	0.008526	0.003185	0.000644	0.147169
0.000129	0.01094	0.035521	0.009299	0.058205	0.051705	0.404086
9.65E-05	0.004151	0.001609	0.018855	0.003571	0.005856	0.170914
9.65E-05	0.016988	0.017825	0.007754	0.002671	0.000901	0.170656
0.008687	3.22E-05	0.000804	0.006725	0.006178	0.007883	0.136036
0.015798	0.152574	3.22E-05	0.001223	0	0.003057	0.167085
0.00029	0.074839	0.006918	0.00695	0.023198	0.031596	0.239382
3.22E-05	3.22E-05	0.002799	0.020077	0.061004	0.000515	0.316248
6.44E-05	0.008398	0.002606	0.005598	0.016828	0.00814	0.182014
0	0.027413	0.013835	0.005405	0.069434	0.014897	0.464254
0.01464	0.001544	0.000804	0.003443	6.44E-05	0.002992	0.096911
0.00074	0.000901	0.007529	0.030438	0.000257	0.001158	0.068436
0.010521	0.00029	0.003282	0.002928	0.049968	0.015798	0.311969
0.000225	0.008366	0.001126	0.004633	0.0074	0.004537	0.242214
0.006885	0.001094	0.007819	0.005438	0.007336	0.002703	0.21953
0	0.005019	0.000611	0.000965	0.002574	0.000901	0.159942
0.000129	0.007432	0.010907	0.000354	0.033752	0.009781	0.3213
0.017149	0.010489	0.00119	0.017664	0.015669	0.037613	0.213481
0.000483	0.025193	0.022619	0.007915	0.009266	0.011615	0.277381
0.006145	0.002574	0.001834	0.019208	0.037548	0.000644	0.191988
0.011712	0.014318	0.000225	0.010135	0.003057	0.002413	0.097008
0.039318	6.44E-05	0.000869	0.03269	0	0.009234	0.124196
6.44E-05	0.000161	0.006628	0.010972	0.006113	0.001384	0.114929
0.023777	0.000161	0.040476	0.004923	0	0.015766	0.148681
0.019048	0.008012	0.001223	0.003893	0.000193	0.019884	0.238127
9.65E-05	0.000676	0.007143	0.003539	0.00592	0.002477	0.118436
0.002059	0.001931	0.013288	0.004054	0.000933	0.001319	0.254279
0.001126	0.001448	0.004183	0.007079	0.000644	0.013353	0.28594
0.000257	0.095592	0.006532	0.002896	0.006435	0.006628	0.176931
0.003057	0.158462	0.011486	0.019788	0.039221	0.069048	0.21982
0.021075	0.005212	0.000354	0.000547	0.00222	0.004376	0.11908
0.022651	0.015862	0.001351	0.010972	0.000193	0.053925	0.249453
0.001995	0.035296	0.005631	0.012387	0.017921	0.032754	0.214221
0.000322	0.006274	0.013192	0.016699	0.010843	0.011229	0.286229
0.000322	0	0.00045	0.013288	0.005502	0.008526	0.241699
0.00045	0.021686	0.010167	0.006435	0.010071	0.038481	0.280277

3.22E-05	0.00045	0.008301	0.008591	0.01583	0.000676	0.112001
0.002156	0.000386	0.012516	0.008301	0.003925	0.002799	0.247329
0.003024	0.00177	0.022072	0.019369	0.013224	0.003282	0.154183
0.125257	0	0.007625	0.011969	0	0.008012	0.104794
0.001641	0.003153	0	0	0.036969	0.004987	0.207625
0.108044	0.012323	0.00045	0.001577	0	0.012387	0.464929
0.050354	0.001834	0.010071	0.052831	0.00029	0.005727	0.188514
3.22E-05	0.01805	0.019048	0.009073	0.011712	0.004633	0.44379
0.001416	0	0	0.005084	0.000161	0.006628	0.075064
0.000869	0.009653	0	0.010779	0.052574	0.020785	0.15325
0.000193	0.005309	0.008623	0.015605	0.003507	0.007175	0.337162
0.037291	0	0	0.020367	0	0.002284	0.240508
0.022748	0.007979	0	0.015862	0	0.059813	0.218565
9.65E-05	0.00045	0.036229	0.014961	0.003829	0.001126	0.24305
3.22E-05	3.22E-05	0.038385	0.014254	0.005502	0.01287	0.333012
0.001094	0.004247	0.014768	0.017342	0.002091	0.002542	0.25045
0.001834	0.001094	0.008494	0.025032	0.005051	0.003829	0.168372
0.000322	0.000193	0.001834	0.022169	0.030695	0.005438	0.330373
0.035232	0.00119	0.002188	0.008398	0.000708	0.003121	0.219788
0	0.001866	0.029891	0.006853	0.001416	0.009106	0.518887
0.005888	0.011422	0.036293	0.013707	0.010907	0.005792	0.204344
3.22E-05	0.000161	0.009974	0.016602	0.005759	0.004054	0.165508
0.001384	0.000515	0.03713	0.017793	0.038288	0.004472	0.171429
0.000997	0.000547	0.038835	0.023295	0.020013	0.0037	0.334749
0.000161	0.003539	0.011004	0.000193	0.002703	0.001834	0.17101
0.002928	0.002124	0	0.039286	0	0.005373	0.11612
0.0537	6.44E-05	0.000193	0.029215	0	0.00177	0.047748
0.080019	0	0.000257	0.017085	3.22E-05	0.01094	0.164543
0.000708	0.001802	0.038578	0.014994	0.004086	0.005084	0.180116
0.006306	0.001898	0.002992	0.009878	0	0.003668	0.254344
0.000161	0.006982	0.014736	0.00029	0.001641	0.000418	0.318018
0.001577	6.44E-05	0.068501	6.44E-05	0.000257	6.44E-05	0.729537
0.000483	3.22E-05	0.039414	0	0.000418	0	0.868726
0.000418	6.44E-05	0.01065	0.005309	0.024646	0.001705	0.187323
0.033816	0.017439	0.007529	0.007561	0.002349	0.011326	0.196847
0.016248	0.001255	0.023842	0.009299	0.001641	0.00843	0.211197
0.009653	0.012066	0.013578	0.006532	0.009588	0.001512	0.344595
0.028411	0.001255	6.44E-05	0.00045	0	0.038707	0.257915

0.001384	0.00045	0.018404	0.012548	0.002928	0.00399	0.113996
0.002349	0.010972	0.004762	0.000322	0.030245	0.00103	0.203024
0.001963	0.007432	0.013256	0.008044	0.001898	0.004183	0.277349
0.001834	0.006725	0.006918	0.020335	0.009974	0.003314	0.34823
0.006564	0.000386	0.00695	0.04157	0.003861	0.004955	0.3389
0.036133	0.00074	0.001641	0.027091	0.000965	0.001351	0.286776
0.008559	0.001931	0.000257	0.003861	0.00029	0	0.052413
0.042053	0.018597	3.22E-05	0.001673	0	9.65E-05	0.400933
0.019369	0.007336	0.023198	0.012259	0.003475	0.000579	0.238417
0.001287	0.010682	0.036808	0.02722	0.005405	0.057658	0.344434
0.058462	0.00029	0	0	0	0.008172	0.112259
0.033655	0.000515	0.018468	0.003121	3.22E-05	0.002477	0.484588
0.007207	0.004698	0.017085	0.020624	0.003153	0.001416	0.206564
0.050708	0.004569	0.054279	0.03417	0	0.00473	0.154794
0.018308	0.007336	0.001094	0.002767	0.000225	0.001705	0.581371
0.04675	0.001416	0.004762	0.003443	0.000322	0.000772	0.172233
0.003571	0	0.013964	0	0.002542	0.001577	0.1963
6.44E-05	0.005116	0.006178	0.010779	0	0.003507	0.195753
0.011165	0.002445	0.027059	0.016731	0.001931	9.65E-05	0.222748
0.000193	6.44E-05	0.01538	0.021236	0.000386	0.00148	0.198198
0.000547	0.001544	0.020302	0.007658	0	0.010714	0.175418
0.000257	0	0.016538	0.019176	0.048616	0.012098	0.265283
0.001544	0.04556	0.005405	0.009459	0.019949	0.020302	0.176094
0.003346	0.017664	0.007593	0.000869	0.016795	0.003475	0.379698
0.01332	0.067954	0.00103	0	0	0.004505	0.185714
0.057851	0	0.020238	0.016634	0.003443	0.010972	0.199035
0.029215	0.000483	0	0	0.004923	0.004762	0.14675
0.012066	0.001158	0.020495	0.020142	0.0139	0.00251	0.14379
0.005727	0.025129	0.014607	0.005373	0.022941	0.065927	0.262548
0.002992	0.040058	0.006403	0	0.03816	0.022169	0.213546
0.00621	0.046622	0.01509	0.014994	0.025579	0.013964	0.304858
0.00888	0.033912	0.002896	0.020689	0.020785	0.01583	0.371107
0.112677	0.000418	0.000515	0.009717	0	0.002606	0.103089
0.01834	0.000225	0.010296	0.03092	0.017825	0.018018	0.242181
0.009717	0.002124	0.00473	0.014414	0.035296	0.00177	0.184234
0.000225	0.003636	0.000483	0.018468	0.006564	0.00074	0.357819
0.015508	0.002864	0.022973	0.030084	0.03018	0.006338	0.222909
0.000386	0.017921	0.012162	0.004537	0.06242	0.000965	0.289994

0.002703	0.023134	0.06686	0.006242	0.091248	0.009395	0.454344
6.44E-05	0.002638	0.006017	0.01731	0.002606	0.007497	0.230245
0.000901	0.000997	0.014254	0.006564	0.022104	0.009556	0.216409
0.022394	0.027381	0.000804	0.0074	0.002477	0.022458	0.215927
0.021943	0.000161	0.004376	0.007658	0.009138	0.000579	0.059459
0.000386	0.002059	0.000869	0	0.000193	0.010296	0.14009
0.1176	0.000997	0.002317	0.008301	0.00045	0.003668	0.077928
0.000129	0.000193	0.023134	0.011036	0.005502	0.001802	0.183462
0.027477	0.137387	6.44E-05	0.00029	6.44E-05	0.11184	0.301673
9.65E-05	0.044691	0.023005	0.01213	0.021139	0.000611	0.140508
0.013127	0.002124	0.024517	0.008494	3.22E-05	0.014414	0.276448
0.001255	0.005985	0.000644	0.00325	0.016248	0.000997	0.168855
0	9.65E-05	0.00695	0.013578	0.009813	0.002574	0.225
0.017246	0.031178	0.011197	0.00962	0.002059	0.00251	0.171622
0.000257	0.027445	0.000161	0.002671	3.22E-05	0.006532	0.234781
0.00119	0	0.001126	0.039382	0.002831	0.004826	0.138514
0.012194	0.005373	0.000225	0.004311	6.44E-05	0.011454	0.140798
0.002445	0.000611	0.012516	0.005116	0.015862	0.014929	0.128443
9.65E-05	0.002349	0.014157	0.011647	0.008752	0.030888	0.323649
0.002317	0.029279	0.002928	0.001255	0	0.008945	0.177542
0.009781	0.00029	0.017761	0.007046	0.00325	0.001544	0.178507
6.44E-05	0.000869	0.000547	0.007336	0.002574	0.000322	0.059685
0.0074	0.007207	0.006532	0.011615	0.000129	0.029022	0.110489
0.060199	0.028346	0	0	0	0.02381	0.123263

Supplementary Table 7 Characteristic bacterial biomarkers screened by LEfSe

tax	Sign_Grou	LDA	FDR	Healthy_m	PCOS_mea	Totalmean	N1	N2
k__Bacteri	Healthy	2.130176	0.000247	0.015071	0.001642	0.005394	0	0.003218
k__Bacteri	Healthy	2.412865	0.001164	0.008721	0	0.002437	0	0.003218
k__Bacteri	Healthy	4.498479	0.035188	14.17818	9.240407	10.62008	0	0.009653
k__Bacteri	Healthy	3.448789	0.020213	0.49956	0.047869	0.174076	0	0
k__Bacteri	Healthy	3.426822	0.020213	0.49956	0.047869	0.174076	0	0
k__Bacteri	PCOS	3.530532	0.043414	1.364052	1.611411	1.542296	0	0.006435
k__Bacteri	PCOS	3.508786	0.043414	1.364052	1.611411	1.542296	0	0.006435
k__Bacteri	Healthy	4.455009	0.000193	12.05463	7.298184	8.627191	0	0.003218
k__Bacteri	Healthy	4.468467	0.000193	12.05463	7.298184	8.627191	0	0.003218
k__Bacteri	Healthy	2.614846	0.023773	0.091614	0.020388	0.04029	0	0
k__Bacteri	Healthy	2.643818	0.023773	0.091614	0.020388	0.04029	0	0
k__Bacteri	PCOS	2.479223	0.010573	0.000169	0.065335	0.047127	0	0
k__Bacteri	Healthy	2.205772	0.023841	0.03209	0.004761	0.012397	0.34749	0.006435
k__Bacteri	Healthy	2.162893	0.023841	0.03209	0.004761	0.012397	0.34749	0.006435
k__Bacteri	Healthy	2.818002	0.005243	0.37196	0.244005	0.279757	0	0.530888
k__Bacteri	Healthy	2.818002	0.005243	0.37196	0.244005	0.279757	0	0.530888
k__Bacteri	Healthy	3.201637	0.034875	1.249153	0.898242	0.99629	0.2574	0.563063
k__Bacteri	Healthy	3.201637	0.034875	1.249153	0.898242	0.99629	0.2574	0.563063
k__Bacteri	Healthy	2.215543	0.005581	0.08374	0.046588	0.056969	0	0.093308
k__Bacteri	Healthy	2.214168	0.005581	0.08374	0.046588	0.056969	0	0.093308
k__Bacteri	PCOS	3.530717	0.04063	0.132426	0.928611	0.706147	0.083655	0.06435
k__Bacteri	PCOS	3.530717	0.04063	0.132426	0.928611	0.706147	0.083655	0.06435
k__Bacteri	Healthy	4.337724	0.041901	21.12189	16.39869	17.71841	15.29601	34.82947
k__Bacteri	Healthy	4.061515	0.042144	11.04992	8.677147	9.340128	9.761905	7.609395
k__Bacteri	Healthy	4.061515	0.042144	11.04992	8.677147	9.340128	9.761905	7.609395
k__Bacteri	Healthy	3.445927	0.039251	1.181755	0.542379	0.721028	1.254826	0.222008
k__Bacteri	Healthy	3.445927	0.039251	1.181755	0.542379	0.721028	1.254826	0.222008
k__Bacteri	Healthy	3.676869	0.01059	2.398733	1.27354	1.587932	0.682111	2.895753
k__Bacteri	Healthy	3.676869	0.01059	2.398733	1.27354	1.587932	0.682111	2.895753
k__Bacteri	Healthy	2.4145	0.03951	0.028365	0.001182	0.008777	0.006435	0.019305
k__Bacteri	Healthy	2.42279	0.03951	0.028365	0.001182	0.008777	0.006435	0.019305
k__Bacteri	PCOS	3.832863	0.004122	2.818956	4.452105	3.995784	5.823681	2.815315
k__Bacteri	Healthy	2.93885	0.00042	0.157996	0.025576	0.062576	0.003218	1.525097
k__Bacteri	Healthy	3.047869	0.000151	0.157996	0.025477	0.062505	0.003218	1.525097
k__Bacteri	Healthy	2.959772	0.000151	0.157996	0.025477	0.062505	0.003218	1.525097
k__Bacteri	Healthy	3.515369	0.022638	0.009483	0	0.00265	0	0
k__Bacteri	Healthy	3.527483	0.022638	0.009483	0	0.00265	0	0
k__Bacteri	Healthy	3.256554	0.000268	0.141909	0	0.039651	0.003218	1.525097
k__Bacteri	Healthy	3.250217	0.000268	0.141909	0	0.039651	0.003218	1.525097
k__Bacteri	Healthy	2.825139	0.00193	0.021083	0.000263	0.00608	0	0
k__Bacteri	Healthy	2.847621	0.00193	0.021083	0.000263	0.00608	0	0
k__Bacteri	PCOS	3.863127	0.001089	2.357668	4.046043	3.574291	5.553411	0.974904
k__Bacteri	PCOS	3.379965	0.023668	1.207156	1.744839	1.594604	1.074646	0.730373
k__Bacteri	PCOS	3.37991	0.023668	1.207156	1.744773	1.594557	1.074646	0.730373

N3	N4	N5	N6	N7	N8	N10	N11	N12
0	0	0	0	0	0.003218	0.022523	0.305663	0
0	0	0	0	0	0.003218	0	0.305663	0
61.9305	0.595238	0.009653	0.006435	0.254183	31.11326	16.44466	1.644144	0.022523
2.931145	0	0	0	0.01287	3.001931	0	0	0
2.931145	0	0	0	0.01287	3.001931	0	0	0
6.901544	0	0	0.003218	0.009653	1.856499	15.89447	0	0
6.901544	0	0	0.003218	0.009653	1.856499	15.89447	0	0
52.09781	0.019305	0.006435	0.003218	0.228443	26.25483	0.009653	1.174389	0.006435
52.09781	0.019305	0.006435	0.003218	0.228443	26.25483	0.009653	1.174389	0.006435
0	0	0.003218	0	0	0	0	0.109395	0
0	0	0.003218	0	0	0	0	0.109395	0
0	0	0	0	0	0	0	0	0
0	0	0	0	0.588803	0	0	0.10296	0
0	0	0	0	0.588803	0	0	0.10296	0
0.196268	0.083655	0.498713	0.02574	0.341055	0.10296	0.350708	1.270914	0.138353
0.196268	0.083655	0.498713	0.02574	0.341055	0.10296	0.350708	1.270914	0.138353
0.07722	0.324968	1.608752	8.133848	1.840412	0.279923	1.132561	3.175676	0.537323
0.07722	0.324968	1.608752	8.133848	1.840412	0.279923	1.132561	3.175676	0.537323
0.035393	0.086873	0.003218	0	0.1287	0.109395	0.225225	0.312098	0.045045
0.035393	0.086873	0.003218	0	0.1287	0.109395	0.225225	0.312098	0.045045
0.01287	0.096525	0.106178	0.01287	0.170528	0.048263	0	0.350708	0.01287
0.01287	0.096525	0.106178	0.01287	0.170528	0.048263	0	0.350708	0.01287
12.45817	26.74389	15.14801	5.733591	38.22716	30.99421	17.12999	34.20849	15.59202
6.615187	19.21493	8.841699	4.533462	20.55985	12.33269	8.330116	5.209138	13.76448
6.615187	19.21493	8.841699	4.533462	20.55985	12.33269	8.330116	5.209138	13.76448
0.894466	0.254183	0.009653	0	4.227799	2.393822	0.447233	0.34749	0.06435
0.894466	0.254183	0.009653	0	4.227799	2.393822	0.447233	0.34749	0.06435
0.292793	3.027671	0.527671	0.01287	5.826898	1.470399	1.409266	5.312098	0.370013
0.292793	3.027671	0.527671	0.01287	5.826898	1.470399	1.409266	5.312098	0.370013
0	0	0	0	0	0	0	0.009653	0
0	0	0	0	0	0	0	0.009653	0
2.007722	1.328829	2.068855	0.601673	1.685972	2.323037	0.978121	0.196268	4.263192
0	0.01287	0	0	0	0	0	0	0
0	0.01287	0	0	0	0	0	0	0
0	0.01287	0	0	0	0	0	0	0
0	0	0	0	0	0	0	0	0
0	0	0	0	0	0	0	0	0
0	0.01287	0	0	0	0	0	0	0
0	0.01287	0	0	0	0	0	0	0
0	0	0	0	0	0	0	0.054698	0
0	0	0	0	0	0	0	0.054698	0
1.924067	1.126126	1.171171	0.601673	1.563707	0.907336	0.923423	0.109395	4.205277
1.673102	0.897683	0.006435	0	0.315315	0.601673	0.891248	0.028958	1.039254
1.673102	0.897683	0.006435	0	0.315315	0.601673	0.891248	0.028958	1.039254

N14	N15	N16	N17	N18	N19	N20	N21	N22
0	0	0.041828	0	0.02574	0	0	0.061133	0
0	0	0	0	0.019305	0	0	0	0
32.45174	0.701416	45.38288	0.160875	52.74775	0.392535	0.022523	0.028958	11.77928
0	0	7.239382	0.003218	4.427284	0	0.003218	0	0
0	0	7.239382	0.003218	4.427284	0	0.003218	0	0
0	0.344273	9.044402	0.07722	2.059202	0	0	0.006435	11.76963
0	0.344273	9.044402	0.07722	2.059202	0	0	0.006435	11.76963
31.84685	0.019305	28.80309	0.07722	46.22909	0.003218	0.016088	0	0.009653
31.84685	0.019305	28.80309	0.07722	46.22909	0.003218	0.016088	0	0.009653
0.006435	0.003218	0	0	0.022523	0.234878	0	0	0
0.006435	0.003218	0	0	0.022523	0.234878	0	0	0
0	0	0	0	0	0	0	0	0
0	0.003218	0.035393	0	0	0.003218	0	0	0
0	0.003218	0.035393	0	0	0.003218	0	0	0
0.183398	0.585586	0.534106	0.173745	0.196268	0.508366	0.836551	0.382883	0.173745
0.183398	0.585586	0.534106	0.173745	0.196268	0.508366	0.836551	0.382883	0.173745
0.20592	1.042471	0.254183	1.000644	0.209138	1.274131	2.918275	1.393179	0.518018
0.20592	1.042471	0.254183	1.000644	0.209138	1.274131	2.918275	1.393179	0.518018
0.009653	0.09009	0.09009	0.006435	0.032175	0.045045	0.209138	0.096525	0
0.009653	0.09009	0.09009	0.006435	0.032175	0.045045	0.209138	0.096525	0
0	0.074003	0.019305	0.315315	0.016088	0.022523	0.45045	0.083655	0.01287
0	0.074003	0.019305	0.315315	0.016088	0.022523	0.45045	0.083655	0.01287
9.925997	58.787	23.64221	25.7529	8.803089	44.10875	26.23874	24.01223	19.16345
6.985199	23.53282	19.57529	10.87516	6.624839	24.8166	8.648649	11.64414	9.095882
6.985199	23.53282	19.57529	10.87516	6.624839	24.8166	8.648649	11.64414	9.095882
0.003218	11.64093	0.083655	0.366795	0.234878	3.217503	0	0.045045	4.099099
0.003218	11.64093	0.083655	0.366795	0.234878	3.217503	0	0.045045	4.099099
1.048906	10.31853	1.270914	5.743243	0.772201	3.667954	9.980695	2.274775	0.05148
1.048906	10.31853	1.270914	5.743243	0.772201	3.667954	9.980695	2.274775	0.05148
0	0	0	0	0	0	0	0.119048	0
0	0	0	0	0	0	0	0.119048	0
2.676963	0.469756	0.920206	2.760618	4.208494	1.435006	0.675676	9.520592	3.349421
0	0	0	0	0.009653	0.189833	0	0	0
0	0	0	0	0.009653	0.189833	0	0	0
0	0	0	0	0.009653	0.189833	0	0	0
0	0	0	0	0	0	0	0	0
0	0	0	0	0	0	0	0	0
0	0	0	0	0	0	0	0	0
0	0	0	0	0	0	0	0	0
0.238095	0	0	0	0.009653	0.041828	0	0.456885	0
0.238095	0	0	0	0.009653	0.041828	0	0.456885	0
2.162162	0.402188	0.846203	2.483913	4.137709	0.694981	0.353925	8.844916	2.741313
1.389961	0.016088	0.363578	2.38417	1.657014	0.646718	0.222008	1.911197	2.055985
1.389961	0.016088	0.363578	2.38417	1.657014	0.646718	0.222008	1.911197	2.055985

N23	N24	N26	N27	N28	N29	N30	N31	N32
0	0	0	0	0	0	0	0	0
0	0	0	0	0	0	0	0	0
0.019305	0.119048	61.41248	0.096525	4.192407	61.23552	0.06435	16.90798	0.022523
0	0	0	0	0.003218	1.354569	0	0	0
0	0	0	0	0.003218	1.354569	0	0	0
0	0.003218	0	0	0	0.032175	0.035393	0	0
0	0.003218	0	0	0	0.032175	0.035393	0	0
0.016088	0.074003	61.10682	0.096525	0.21879	59.6686	0	16.90798	0.022523
0.016088	0.074003	61.10682	0.096525	0.21879	59.6686	0	16.90798	0.022523
0	0	0.238095	0	2.863578	0	0	0	0
0	0	0.238095	0	2.863578	0	0	0	0
0	0	0	0.003218	0	0	0	0	0.003218
0	0	0	0	0	0	0.003218	0.003218	0
0	0	0	0	0	0	0.003218	0.003218	0
0.106178	0.1287	0.148005	0.173745	0.405405	0.122265	0.138353	0.460103	0
0.106178	0.1287	0.148005	0.173745	0.405405	0.122265	0.138353	0.460103	0
0.23166	0.427928	0.511583	0.974903	1.843629	0.260618	1.396396	1.853282	1.344916
0.23166	0.427928	0.511583	0.974903	1.843629	0.260618	1.396396	1.853282	1.344916
0.01287	0.022523	0.061133	0	0.070785	0.028958	0.028958	0.083655	0.267053
0.01287	0.022523	0.061133	0	0.070785	0.028958	0.028958	0.083655	0.267053
0.070785	0.093308	0.173745	0.009653	0.186615	0.006435	0.370013	0.215573	0.048263
0.070785	0.093308	0.173745	0.009653	0.186615	0.006435	0.370013	0.215573	0.048263
28.98005	10.34749	12.36486	5.20592	17.94402	10.81725	21.1036	18.23037	24.24389
21.98842	7.728443	8.481338	0.016088	12.45495	5.022523	17.96654	12.23616	13.45882
21.98842	7.728443	8.481338	0.016088	12.45495	5.022523	17.96654	12.23616	13.45882
0.254183	0.003218	0.817246	0	0.067568	0.173745	0.775418	0.048263	4.350064
0.254183	0.003218	0.817246	0	0.067568	0.173745	0.775418	0.048263	4.350064
2.57722	0.215573	0.637066	0.598456	2.516088	1.798584	0.041828	1.312741	0.617761
2.57722	0.215573	0.637066	0.598456	2.516088	1.798584	0.041828	1.312741	0.617761
0	0.019305	0	0.009653	0	0	0.016088	0	0
0	0.019305	0	0.009653	0	0	0.016088	0	0
1.991635	6.341699	0.476191	4.90991	1.486487	1.779279	14.33076	3.597169	2.149292
0	0	0	0.2574	0.048263	0	0	0	0
0	0	0	0.2574	0.048263	0	0	0	0
0	0	0	0.2574	0.048263	0	0	0	0
0	0	0	0.2574	0	0	0	0	0
0	0	0	0.2574	0	0	0	0	0
0	0	0	0	0	0	0	0	0
0	0	0	0	0	0	0	0	0
0	0	0	0	0	0	0	0	0
0	0	0	0	0	0	0	0	0
1.805019	5.839768	0.273488	3.729086	1.402831	1.406049	14.23102	3.445946	2.081725
1.660232	5.2574	0.170528	3.500644	1.325611	1.290219	4.105534	0.765766	1.184041
1.660232	5.2574	0.170528	3.500644	1.325611	1.290219	4.105534	0.765766	1.184041

N33	N34	N35	N36	N37	N38	N39	N40	N41
0.006435	0.045045	0.032175	0.022523	0	0	0	0.003218	0
0	0	0	0	0	0	0	0	0
2.380952	50.55341	0.122265	0.598456	0.395753	32.56435	0.119048	51.44144	0.826898
0	0	0.006435	0	0	0	0	0	0
0	0	0.006435	0	0	0	0	0	0
0.003218	0.019305	0.003218	0	0.003218	3.751609	0.009653	0	0
0.003218	0.019305	0.003218	0	0.003218	3.751609	0.009653	0	0
1.315959	50.53411	0.112613	0.003218	0.006435	28.80631	0.109395	51.44144	0.826898
1.315959	50.53411	0.112613	0.003218	0.006435	28.80631	0.109395	51.44144	0.826898
0	0	0	0	0	0	0	0	0
0	0	0	0	0	0	0	0	0
0	0	0	0	0	0	0	0	0
0.11583	0.003218	0	0	0.003218	0	0	0.003218	0
0.11583	0.003218	0	0	0.003218	0	0	0.003218	0
1.122909	0.19305	1.068211	0.685328	0.794723	0.765766	0.276705	0.183398	0.247748
1.122909	0.19305	1.068211	0.685328	0.794723	0.765766	0.276705	0.183398	0.247748
3.877091	0.315315	3.632561	0.791506	1.396396	0.344273	1.036036	0.27027	0.212355
3.877091	0.315315	3.632561	0.791506	1.396396	0.344273	1.036036	0.27027	0.212355
0.074003	0.109395	0.009653	0.054698	0.228443	0.241313	0.148005	0.07722	0.045045
0.074003	0.109395	0.009653	0.054698	0.228443	0.241313	0.148005	0.07722	0.045045
0.199485	1.142214	0.131918	0.003218	0.02574	0.106178	0.009653	0.05148	0.234878
0.199485	1.142214	0.131918	0.003218	0.02574	0.106178	0.009653	0.05148	0.234878
19.16023	10.19305	6.425354	25.41506	28.30759	15.74968	25.01609	12.50322	23.82883
8.317246	7.35843	4.189189	11.76963	8.635779	6.203346	14.03475	6.647362	14.8166
8.317246	7.35843	4.189189	11.76963	8.635779	6.203346	14.03475	6.647362	14.8166
0.913771	0.402188	0.019305	0.222008	4.925997	0.341055	0.839768	0.511583	0.434363
0.913771	0.402188	0.019305	0.222008	4.925997	0.341055	0.839768	0.511583	0.434363
2.921493	1.367439	0.05148	6.785714	6.576577	0.955598	1.743887	2.425997	1.052124
2.921493	1.367439	0.05148	6.785714	6.576577	0.955598	1.743887	2.425997	1.052124
0.14157	0	0	0	0.736808	0	0	0	0
0.14157	0	0	0	0.736808	0	0	0	0
0.746461	1.901544	3.745174	1.161519	1.576577	1.219434	3.108108	1.650579	4.839125
0.003218	0	0	0	0	0	2.329472	0	1.624839
0.003218	0	0	0	0	0	2.329472	0	1.624839
0.003218	0	0	0	0	0	2.329472	0	1.624839
0	0	0	0	0	0	0.10296	0	0
0	0	0	0	0	0	0.10296	0	0
0	0	0	0	0	0	2.226512	0	1.624839
0	0	0	0	0	0	2.226512	0	1.624839
0	0	0	0	0	0	0	0	0
0	0	0	0	0	0	0	0	0
0.524453	1.862934	3.577864	0.997426	0.762548	0.888031	0.659588	1.373874	3.001931
0.344273	0.675676	2.16538	0.740026	0.299228	0.855856	0.392535	0.36036	2.87323
0.344273	0.675676	2.16538	0.740026	0.299228	0.855856	0.392535	0.36036	2.87323

P001	P002	P003	P004	P005	P006	P007	P008	P009
0	0	0	0	0	0	0	0	0
0	0	0	0	0	0	0	0	0
0.006435	0	2.567568	0	7.213642	1.599099	27.95367	0	0
0	0	1.161519	0	0	0	0	0	0
0	0	1.161519	0	0	0	0	0	0
0	0	0	0	0	1.116474	27.93436	0	0
0	0	0	0	0	1.116474	27.93436	0	0
0	0	0	0	7.158945	0	0.019305	0	0
0	0	0	0	7.158945	0	0.019305	0	0
0.006435	0	0	0	0	0	0	0	0
0.006435	0	0	0	0	0	0	0	0
0	0	0.070785	0	0	0	0	0.119048	0.003218
0	0	0	0	0.006435	0.028958	0	0.003218	0
0	0	0	0	0.006435	0.028958	0	0.003218	0
0.10296	0.135135	0.022523	0.273488	0.228443	0.10296	0.003218	0.267053	0.260618
0.10296	0.135135	0.022523	0.273488	0.228443	0.10296	0.003218	0.267053	0.260618
0.267053	0.199485	0.46332	0.820463	0.254183	0.357143	0.112613	1.106821	0.299228
0.267053	0.199485	0.46332	0.820463	0.254183	0.357143	0.112613	1.106821	0.299228
0	0	0	0	0	0.080438	0.009653	0.057915	0.02574
0	0	0	0	0	0.080438	0.009653	0.057915	0.02574
0.48906	0	0	8.063063	0.14157	0.148005	0.061133	0.286358	0.093308
0.48906	0	0	8.063063	0.14157	0.148005	0.061133	0.286358	0.093308
16.49936	7.35843	18.30759	17.81532	10.6982	33.38803	1.695624	25.3861	23.83205
10.9556	4.028314	8.806306	13.9897	8.262548	11.40927	1.106821	14.60103	9.401544
10.9556	4.028314	8.806306	13.9897	8.262548	11.40927	1.106821	14.60103	9.401544
0.469755	0	1.148649	0	0	0.29601	0	0.083655	0.485843
0.469755	0	1.148649	0	0	0.29601	0	0.083655	0.485843
0.067568	1.061776	0.839768	0.003218	0.344273	3.973616	0.016088	0.028958	2.545045
0.067568	1.061776	0.839768	0.003218	0.344273	3.973616	0.016088	0.028958	2.545045
0	0	0	0	0	0	0	0	0
0	0	0	0	0	0	0	0	0
3.246461	3.249678	3.957529	0.392535	6.534749	1.756757	1.489704	1.389961	6.212999
0	0	0	0	0.009653	0	0	0	0
0	0	0	0	0	0	0	0	0
0	0	0	0	0	0	0	0	0
0	0	0	0	0	0	0	0	0
0	0	0	0	0	0	0	0	0
0	0	0	0	0	0	0	0	0
0	0	0	0	0	0	0	0	0
0	0	0	0	0	0	0	0	0
0	0	0	0	0	0	0	0	0
2.490348	3.243243	3.957529	0.37323	5.698198	1.409266	1.476834	1.338481	5.881596
1.628057	1.036036	1.377091	0.15444	2.16538	1.299871	1.277349	0.852638	4.324324
1.628057	1.036036	1.377091	0.15444	2.16538	1.299871	1.277349	0.852638	4.317889

P010	P012	P013	P014	P015	P016	P017	P018	P019
0	0	0	0	0	0	0	0	0
0	0	0	0	0	0	0	0	0
1.315959	0	0.003218	0.299228	0.720721	23.82883	26.1583	0	0.183398
0	0	0	0	0	0	1.782497	0	0
0	0	0	0	0	0	1.782497	0	0
0	0	0	0	0	0	9.52381	0	0
0	0	0	0	0	0	9.52381	0	0
0	0	0	0	0	23.60682	13.81918	0	0
0	0	0	0	0	23.60682	13.81918	0	0
0	0	0	0	0	0	0	0	0
0	0	0	0	0	0	0	0	0
0	0.03861	0	0	0	0.003218	0	0	0.035393
0	0	0	0	0	0	0.06435	0	0
0	0	0	0	0	0	0.06435	0	0
0.344273	0.794723	0.093308	0.14157	0.070785	0.125483	0.099743	0.18018	0.067568
0.344273	0.794723	0.093308	0.14157	0.070785	0.125483	0.099743	0.18018	0.067568
0.109395	0	0.501931	1.287001	0.627413	1.090734	0.151223	0.775418	1.895109
0.109395	0	0.501931	1.287001	0.627413	1.090734	0.151223	0.775418	1.895109
0.003218	0	0.067568	0.186615	0.112613	0.03861	0.109395	0.006435	0.086873
0.003218	0	0.067568	0.186615	0.112613	0.03861	0.109395	0.006435	0.086873
0	2.882883	0.16731	0.048263	0.479408	0.41184	0.444015	1.132561	0.199485
0	2.882883	0.16731	0.048263	0.479408	0.41184	0.444015	1.132561	0.199485
6.306306	15.67889	17.44208	20.30888	21.8018	23.9704	22.72523	10.06435	26.43501
5.09009	12.19755	5.225225	6.122909	10.81081	17.69627	8.423423	4.095882	9.137709
5.09009	12.19755	5.225225	6.122909	10.81081	17.69627	8.423423	4.095882	9.137709
0	0.070785	3.092021	3.124196	3.304376	0	0.814028	0.324968	0.588803
0	0.070785	3.092021	3.124196	3.304376	0	0.814028	0.324968	0.588803
0.041828	0.05148	1.602317	2.004505	0.772201	1.460746	1.087516	1.312741	2.01094
0.041828	0.05148	1.602317	2.004505	0.772201	1.460746	1.087516	1.312741	2.01094
0	0	0	0.032175	0.006435	0	0.006435	0	0
0	0	0	0.032175	0.006435	0	0.006435	0	0
6.570142	2.503218	4.761905	1.492922	3.552124	2.8861	4.041184	0.791506	4.057272
0	0	0	0	0	0	0	0	0.656371
0	0	0	0	0	0	0	0	0.656371
0	0	0	0	0	0	0	0	0.656371
0	0	0	0	0	0	0	0	0
0	0	0	0	0	0	0	0	0
0	0	0	0	0	0	0	0	0
0	0	0	0	0	0	0	0	0
0	0	0	0	0	0	0	0	0
0	0	0	0	0	0	0	0	0
6.106821	2.503218	4.430502	1.290219	3.365508	2.59009	3.018018	0.707851	2.52574
5.701416	2.464607	2.8861	1.003861	2.657658	1.792149	2.20399	0.559846	2.432432
5.701416	2.464607	2.8861	1.003861	2.657658	1.792149	2.20399	0.559846	2.432432

P020	P021	P022	P023	P024	P025	P026	P027	P028
0	0	0	0	0	0	0	0	0
0	0	0	0	0	0	0	0	0
0.003218	48.53604	0	0	16.68919	40.72072	0	0	0
0	0	0	0	0	0	0	0	0
0	0	0	0	0	0	0	0	0
0	1.261261	0	0	0	0	0	0	0
0	1.261261	0	0	0	0	0	0	0
0	47.11068	0	0	16.67954	40.69498	0	0	0
0	47.11068	0	0	16.67954	40.69498	0	0	0
0	0	0	0	0	0	0	0	0
0	0	0	0	0	0	0	0	0
0	0	0	0	0.003218	0	0	0	0
0	0	0	0	0.01287	0.003218	0	0	0
0	0	0	0	0.01287	0.003218	0	0	0
0	0.061133	0.070785	0	0.27027	0.003218	0	1.048906	0.263835
0	0.061133	0.070785	0	0.27027	0.003218	0	1.048906	0.263835
0.344273	0.34749	1.315959	0.19305	1.032819	0.144788	0	0.756113	1.119691
0.344273	0.34749	1.315959	0.19305	1.032819	0.144788	0	0.756113	1.119691
0.01287	0.028958	0.035393	0	0.138353	0.032175	0	0.006435	0.074003
0.01287	0.028958	0.035393	0	0.138353	0.032175	0	0.006435	0.074003
0.112613	0.003218	0.176963	0.070785	0.415058	0	0.028958	0.21879	0.164093
0.112613	0.003218	0.176963	0.070785	0.415058	0	0.028958	0.21879	0.164093
13.18855	18.2529	20.18018	16.05534	19.72651	8.240026	0.704633	3.301158	19.13449
5.768983	6.332046	8.976834	4.427284	13.85135	6.734234	0.122265	1.045689	8.468468
5.768983	6.332046	8.976834	4.427284	13.85135	6.734234	0.122265	1.045689	8.468468
1.341699	1.299871	0.730373	0.826898	1.052124	0	0	0	0.131918
1.341699	1.299871	0.730373	0.826898	1.052124	0	0	0	0.131918
0.929858	3.027671	1.058559	1.904762	1.415701	0.045045	0.009653	0.02574	4.269627
0.929858	3.027671	1.058559	1.904762	1.415701	0.045045	0.009653	0.02574	4.269627
0.016088	0	0	0	0	0	0	0	0
0.016088	0	0	0	0	0	0	0	0
7.992278	1.827542	2.12677	2.081725	4.111969	7.503218	7.007722	6.180824	2.738095
0	0	0	0	0	0	0	0	0
0	0	0	0	0	0	0	0	0
0	0	0	0	0	0	0	0	0
0	0	0	0	0	0	0	0	0
0	0	0	0	0	0	0	0	0
0	0	0	0	0	0	0	0	0
0	0	0	0	0	0	0	0	0
0	0	0	0	0	0	0	0	0
0	0	0	0	0	0	0	0	0
7.310167	1.412484	0.746461	1.566924	4.057272	7.5	5.984556	5.859073	2.133205
6.483269	1.396396	0.678893	1.515444	1.319176	6.032819	0	3.027671	2.10103
6.483269	1.396396	0.678893	1.515444	1.319176	6.032819	0	3.027671	2.10103

P030	P031	P033	P034	P035	P036	P037	P038	P039
0	0	0	0	0	0	0	0.006435	0
0	0	0	0	0	0	0	0	0
0	20.80438	0.540541	4.025097	0.003218	0.006435	0.11583	0.968468	0.016088
0	0	0.057915	0.933076	0.003218	0	0	0	0
0	0	0.057915	0.933076	0.003218	0	0	0	0
0	0	0	0	0	0	0	0	0.003218
0	0	0	0	0	0	0	0	0.003218
0	20.49228	0.02574	0	0	0	0	0	0.01287
0	20.49228	0.02574	0	0	0	0	0	0.01287
0	0	0.032175	0.328185	0	0	0	0	0
0	0	0.032175	0.328185	0	0	0	0	0
0	0	0.003218	0	0	0.772201	0	0	0.756113
0	0	0	0	0	0	0	0.016088	0
0	0	0	0	0	0	0	0.016088	0
1.097169	0.1287	0.006435	0.003218	0.202703	1.515444	0.045045	0.18018	0.176963
1.097169	0.1287	0.006435	0.003218	0.202703	1.515444	0.045045	0.18018	0.176963
0.608108	0.32175	0.006435	0	0.176963	0.862291	0.006435	1.573359	2.158945
0.608108	0.32175	0.006435	0	0.176963	0.862291	0.006435	1.573359	2.158945
0.045045	0.350708	0	0	0.016088	0.032175	0.035393	0.041828	0.14157
0.045045	0.350708	0	0	0.016088	0.032175	0.035393	0.041828	0.14157
0.962033	0.21879	0	0	0.920206	2.985843	0.093308	0.32175	1.631274
0.962033	0.21879	0	0	0.920206	2.985843	0.093308	0.32175	1.631274
12.06564	26.48327	6.148649	3.754826	21.2677	19.07658	20	20.54376	14.80695
9.292149	13.96396	0.02574	0	9.333977	12.78314	6.048906	6.531532	13.5843
9.292149	13.96396	0.02574	0	9.333977	12.78314	6.048906	6.531532	13.5843
0	0.807593	0.06435	0.003218	0.119048	0.01287	1.010296	1.653797	0
0	0.807593	0.06435	0.003218	0.119048	0.01287	1.010296	1.653797	0
0.14157	0.910553	0.006435	0	1.029601	2.223295	0.563063	1.016731	0.27027
0.14157	0.910553	0.006435	0	1.029601	2.223295	0.563063	1.016731	0.27027
0	0.016088	0	0	0	0	0	0	0
0	0.016088	0	0	0	0	0	0	0
9.559202	1.563707	0.2574	0.575933	5.862291	6.357786	1.895109	5.614543	6.99807
0	0	0	0	0	0	0	0	0
0	0	0	0	0	0	0	0	0
0	0	0	0	0	0	0	0	0
0	0	0	0	0	0	0	0	0
0	0	0	0	0	0	0	0	0
0	0	0	0	0	0	0	0	0
0	0	0	0	0	0	0	0	0
0	0	0	0	0	0	0	0	0
0	0	0	0	0	0	0	0	0
9.49807	1.476834	0.1287	0.453668	5.666023	6.354569	1.689189	5.131918	6.99807
9.079794	1.248391	0.016088	0.453668	0.328185	0.682111	1.657014	1.901544	0.492278
9.079794	1.248391	0.016088	0.453668	0.328185	0.682111	1.657014	1.901544	0.492278

P040	P041	P042	P043	P044	P045	P046	P047	P048
0	0	0	0	0	0	0	0	0
0	0	0	0	0	0	0	0	0
0.003218	25.03539	0	12.69627	0.02574	18.70656	0.43758	0	0.981338
0	0	0	0	0	0	0	0	0
0	0	0	0	0	0	0	0	0
0	25.03539	0	12.69627	0.003218	0	0	0	0
0	25.03539	0	12.69627	0.003218	0	0	0	0
0	0	0	0	0	18.70656	0	0	0
0	0	0	0	0	18.70656	0	0	0
0	0	0	0	0	0	0	0	0
0	0	0	0	0	0	0	0	0
0	0.698198	0	0	0	0	0	0.968468	0
0	0	0.032175	0.003218	0	0	0	0	0
0	0	0.032175	0.003218	0	0	0	0	0
0.469755	0.054698	0.135135	0.106178	0.032175	0.164093	0	0.006435	0.086873
0.469755	0.054698	0.135135	0.106178	0.032175	0.164093	0	0.006435	0.086873
0.48906	0.810811	2.619048	0.740026	0.312098	0.006435	0.003218	5.331403	0.540541
0.48906	0.810811	2.619048	0.740026	0.312098	0.006435	0.003218	5.331403	0.540541
0.05148	0.070785	0.067568	0.06435	0	0.003218	0	0	0.016088
0.05148	0.070785	0.067568	0.06435	0	0.003218	0	0	0.016088
0.119048	0	0.057915	0.080438	0.357143	0.408623	0.009653	1.811454	0.138353
0.119048	0	0.057915	0.080438	0.357143	0.408623	0.009653	1.811454	0.138353
9.140927	25.66602	19.16345	16.49292	23.52317	1.927284	0.579151	1.589447	13.36873
4.758687	14.45624	12.58044	3.741956	1.190476	0.743243	0.019305	0	6.788932
4.758687	14.45624	12.58044	3.741956	1.190476	0.743243	0.019305	0	6.788932
1.669884	0.524453	0.534106	0.746461	0	0.022523	0	0	1.171171
1.669884	0.524453	0.534106	0.746461	0	0.022523	0	0	1.171171
0.434363	2.56435	1.747104	1.138996	4.253539	0.344273	0.048263	0	1.361004
0.434363	2.56435	1.747104	1.138996	4.253539	0.344273	0.048263	0	1.361004
0	0	0	0	0	0	0	0.003218	0.003218
0	0	0	0	0	0	0	0.003218	0.003218
3.310811	2.892535	5.267053	2.956886	5.20592	7.496783	59.85199	13.77413	4.218147
0	0	0	0	0.881596	0	0	0	0
0	0	0	0	0.881596	0	0	0	0
0	0	0	0	0.881596	0	0	0	0
0	0	0	0	0	0	0	0	0
0	0	0	0	0	0	0	0	0
0	0	0	0	0	0	0	0	0
0	0	0	0	0	0	0	0	0
0	0	0	0	0	0	0	0	0
0	0	0	0	0	0	0	0	0
3.243243	2.580438	4.877735	2.786358	2.51287	7.184685	58.21429	13.77413	3.941441
1.962677	2.300515	2.702703	2.53861	1.644144	1.364221	0.160875	0	1.512227
1.962677	2.300515	2.702703	2.53861	1.644144	1.364221	0.160875	0	1.512227

P049	P050	P051	P052	P053	P054	P056	P057	P058
0.006435	0	0	0.045045	0	0	0	0	0
0	0	0	0	0	0	0	0	0
0.006435	0	0	16.30952	0	15.76255	5.910553	0.003218	0
0	0	0	0	0	0.003218	0	0	0
0	0	0	0	0	0.003218	0	0	0
0	0	0	0	0	6.129344	5.910553	0	0
0	0	0	0	0	6.129344	5.910553	0	0
0	0	0	11.03604	0	9.60103	0	0	0
0	0	0	11.03604	0	9.60103	0	0	0
0	0	0	0	0	0	0	0	0
0	0	0	0	0	0	0	0	0
0	0	0	0	0	0	0	0	0
0.003218	0	0	0	0	0	0	0	0
0.003218	0	0	0	0	0	0	0	0
0.170528	0.02574	0.608108	0.032175	0.096525	0.016088	0.006435	0.035393	0.138353
0.170528	0.02574	0.608108	0.032175	0.096525	0.016088	0.006435	0.035393	0.138353
2.776705	0	0.003218	0.543758	0	0.2574	0.028958	0.482625	0.427928
2.776705	0	0.003218	0.543758	0	0.2574	0.028958	0.482625	0.427928
0.096525	0	0.03861	0	0	0.003218	0.009653	0.016088	0.041828
0.096525	0	0.03861	0	0	0.003218	0.009653	0.016088	0.041828
0.016088	0.03861	1.692407	0.032175	0.041828	48.05341	0.138353	0.23166	0.006435
0.016088	0.03861	1.692407	0.032175	0.041828	48.05341	0.138353	0.23166	0.006435
18.35586	2.86036	4.7426	19.05084	8.564994	1.930502	5.225225	11.1583	33.83848
7.686615	0	2.97619	14.07658	0	1.599099	3.088803	3.909266	19.49485
7.686615	0	2.97619	14.07658	0	1.599099	3.088803	3.909266	19.49485
0.907336	0	0	0	0	0	0.01287	0.466538	0.003218
0.907336	0	0	0	0	0	0.01287	0.466538	0.003218
4.089447	0.035393	0.247748	0.138353	3.262548	0.045045	0.03861	1.071429	3.268983
4.089447	0.035393	0.247748	0.138353	3.262548	0.045045	0.03861	1.071429	3.268983
0	0	0	0	0	0	0	0	0
0	0	0	0	0	0	0	0	0
4.45946	0.875161	6.135779	2.902188	3.712999	11.39318	4.388674	2.223295	1.759974
0	0	0	0	0	0	0	0	0
0	0	0	0	0	0	0	0	0
0	0	0	0	0	0	0	0	0
0	0	0	0	0	0	0	0	0
0	0	0	0	0	0	0	0	0
0	0	0	0	0	0	0	0	0
0	0	0	0	0	0	0	0	0
0	0	0	0	0	0	0	0	0
0	0	0	0	0	0	0	0	0
4.012227	0.842986	6.042471	1.676319	2.26834	11.38996	4.231017	1.528314	1.759974
3.581081	0	1.013514	1.576577	1.142214	0.569498	4.179537	1.293436	1.753539
3.581081	0	1.013514	1.576577	1.142214	0.569498	4.179537	1.293436	1.753539

P059	P060	P061	P062	P063	P064	P065	P066	P067
0	0	0	0	0	0	0	0	0
0	0	0	0	0	0	0	0	0
0.756113	12.34234	0.263835	3.970399	0.006435	6.972329	0	2.13964	0.006435
0.003218	0.003218	0	0	0	0	0	0	0
0.003218	0.003218	0	0	0	0	0	0	0
0	12.33912	0	0.353925	0.006435	6.415701	0	0	0.003218
0	12.33912	0	0.353925	0.006435	6.415701	0	0	0.003218
0	0	0	2.91184	0	0	0	2.133205	0.003218
0	0	0	2.91184	0	0	0	2.133205	0.003218
0	0	0	0	0	0	0	0	0
0	0	0	0	0	0	0	0	0
0	0.009653	0	0.003218	0	0	0.241313	0	0.009653
0	0	0.035393	0	0.176963	0	0	0	0
0	0	0.035393	0	0.176963	0	0	0	0
0.022523	0.656371	0.344273	0.778636	0.28314	0.318533	0	0.028958	0.408623
0.022523	0.656371	0.344273	0.778636	0.28314	0.318533	0	0.028958	0.408623
0.03861	0.119048	0.900901	0.653153	1.013514	0.955598	0.003218	1.821107	0
0.03861	0.119048	0.900901	0.653153	1.013514	0.955598	0.003218	1.821107	0
0.02574	0.019305	0.144788	0.093308	0.045045	0.045045	0.006435	0.01287	0
0.02574	0.019305	0.144788	0.093308	0.045045	0.045045	0.006435	0.01287	0
0.009653	0.659588	0.173745	0.131918	0.11583	0.125483	0.341055	1.718147	0.016088
0.009653	0.659588	0.173745	0.131918	0.11583	0.125483	0.341055	1.718147	0.016088
15.33462	8.104891	31.6731	34.58816	25.78507	28.38803	0.916988	8.120978	24.54955
1.985199	4.83269	22.85393	19.21815	17.75418	13.19176	0.009653	3.658301	20.05148
1.985199	4.83269	22.85393	19.21815	17.75418	13.19176	0.009653	3.658301	20.05148
0.151223	0.106178	0.006435	0.302445	0	1.119691	0	0	0
0.151223	0.106178	0.006435	0.302445	0	1.119691	0	0	0
2.419562	0.318533	2.78314	1.650579	0.768983	3.507079	0	0.791506	0.035393
2.419562	0.318533	2.78314	1.650579	0.768983	3.507079	0	0.791506	0.035393
0	0	0	0	0	0	0	0	0
0	0	0	0	0	0	0	0	0
2.35843	6.512227	2.580438	1.180824	1.068211	2.70592	11.48649	4.543115	4.777992
0	0	0	0	0	0	0	0	0
0	0	0	0	0	0	0	0	0
0	0	0	0	0	0	0	0	0
0	0	0	0	0	0	0	0	0
0	0	0	0	0	0	0	0	0
0	0	0	0	0	0	0	0	0
0	0	0	0	0	0	0	0	0
0	0	0	0	0	0	0	0	0
0	0	0	0	0	0	0	0	0
2.146075	5.495496	2.08816	0.913771	0.823681	2.261905	11.48649	3.880309	4.777992
1.933719	4.388674	1.267696	0.888031	0.003218	2.01094	4.301802	0.965251	3.809524
1.933719	4.388674	1.267696	0.888031	0.003218	2.01094	4.301802	0.965251	3.809524

P068	P069	P070	P071	P072	P073	P074	P075	P076
0	0	0	0	0	0	0.028958	0	0
0	0	0	0	0	0	0	0	0
0.009653	0.170528	0.009653	1.029601	0.006435	0.006435	0.135135	0.006435	36.5991
0	0	0	0.736808	0	0	0	0	0
0	0	0	0.736808	0	0	0	0	0
0	0	0	0	0.003218	0	0	0	4.379022
0	0	0	0	0.003218	0	0	0	4.379022
0.009653	0.003218	0.006435	0.003218	0.003218	0.003218	0.01287	0.003218	30.87838
0.009653	0.003218	0.006435	0.003218	0.003218	0.003218	0.01287	0.003218	30.87838
0	0	0	0	0	0	0	0	0
0	0	0	0	0	0	0	0	0
0	0	0	0	0	0	0	0	0.009653
0	0	0	0	0	0	0	0	0
0	0	0	0	0	0	0	0	0
0.42471	0.518018	0.003218	0.582368	0.003218	0.109395	0.814028	0.324968	0.099743
0.42471	0.518018	0.003218	0.582368	0.003218	0.109395	0.814028	0.324968	0.099743
2.451737	1.846847	1.222651	0.733591	4.259974	0.846203	3.610039	0.630631	0.070785
2.451737	1.846847	1.222651	0.733591	4.259974	0.846203	3.610039	0.630631	0.070785
0.054698	0.15444	0	0.045045	0.11583	0.003218	0.07722	0.21879	0.067568
0.054698	0.15444	0	0.045045	0.11583	0.003218	0.07722	0.21879	0.067568
0.057915	0.627413	0.189833	0.01287	0.627413	0.186615	0.003218	0.119048	0
0.057915	0.627413	0.189833	0.01287	0.627413	0.186615	0.003218	0.119048	0
18.00837	28.70013	25.96525	29.33076	24.58816	19.18275	13.49743	23.81274	10.98777
10.86229	10.66924	13.90605	14.71042	13.77413	17.19434	4.835907	10.28636	6.907979
10.86229	10.66924	13.90605	14.71042	13.77413	17.19434	4.835907	10.28636	6.907979
0.688546	0.3861	4.269627	0.215573	0.749678	0	0.447233	1.151866	0.659588
0.688546	0.3861	4.269627	0.215573	0.749678	0	0.447233	1.151866	0.659588
0.691763	4.314672	0.009653	6.261261	1.637709	0.045045	1.869369	2.635135	2.07529
0.691763	4.314672	0.009653	6.261261	1.637709	0.045045	1.869369	2.635135	2.07529
0	0	0	0	0.003218	0	0.009653	0	0
0	0	0	0	0.003218	0	0.009653	0	0
1.669884	0.965251	5.511583	1.258044	10.35071	3.114543	1.705277	0.743243	7.631918
0	0	0	0	0	0	0	0	0
0	0	0	0	0	0	0	0	0
0	0	0	0	0	0	0	0	0
0	0	0	0	0	0	0	0	0
0	0	0	0	0	0	0	0	0
0	0	0	0	0	0	0	0	0
0	0	0	0	0	0	0	0	0
0	0	0	0	0	0	0	0	0
0	0	0	0	0	0	0	0	0
1.447876	0.823681	5.23166	0.913771	10.09653	3.114543	1.090734	0.228443	6.833977
1.132561	0.582368	4.083012	0.48906	0.659588	2.454955	0.852638	0.06435	2.863578
1.132561	0.582368	4.083012	0.48906	0.659588	2.454955	0.852638	0.06435	2.863578

P077	P078	P079	P080	P081	P082	P083	P084	P085
0	0.05148	0	0	0	0	0	0	0
0	0	0	0	0	0	0	0	0
9.97426	3.947876	0.006435	1.229086	0.019305	0.003218	50.65959	68.28507	3.822394
0	0	0	0	0	0	0	0	0
0	0	0	0	0	0	0	0	0
0	0	0	0	0	0	7.046332	14.44659	3.822394
0	0	0	0	0	0	7.046332	14.44659	3.822394
9.97426	3.944659	0.006435	0.006435	0.003218	0.003218	43.61326	53.12098	0
9.97426	3.944659	0.006435	0.006435	0.003218	0.003218	43.61326	53.12098	0
0	0	0	0.723938	0.003218	0	0	0.711068	0
0	0	0	0.723938	0.003218	0	0	0.711068	0
0.003218	0	0.016088	0	0	0	0	0	0
0.045045	0	0	0	0	0	0	0	0
0.045045	0	0	0	0	0	0	0	0
0.157658	0.698198	0.382883	0.183398	0	1.026384	0.14157	0.10296	0.035393
0.157658	0.698198	0.382883	0.183398	0	1.026384	0.14157	0.10296	0.035393
0.949163	0.601673	2.709138	2.741313	1.058559	2.181467	0.196268	0.260618	0.260618
0.949163	0.601673	2.709138	2.741313	1.058559	2.181467	0.196268	0.260618	0.260618
0.057915	0.06435	0.160875	0.09009	0.028958	0.24453	0.03861	0.006435	0.009653
0.057915	0.06435	0.160875	0.09009	0.028958	0.24453	0.03861	0.006435	0.009653
0.041828	0.164093	0.135135	0.041828	0.119048	0.759331	0.009653	0.611326	0.032175
0.041828	0.164093	0.135135	0.041828	0.119048	0.759331	0.009653	0.611326	0.032175
30.82046	29.8166	46.25483	29.09588	26.55084	21.77284	8.873874	6.772844	6.309524
15.91055	16.77606	3.719434	20.48584	16.65701	17.80888	5	5.33462	5.39897
15.91055	16.77606	3.719434	20.48584	16.65701	17.80888	5	5.33462	5.39897
1.155084	0.688546	0.569498	5.074003	1.026384	0.057915	1.869369	0.836551	0
1.155084	0.688546	0.569498	5.074003	1.026384	0.057915	1.869369	0.836551	0
2.506435	2.055985	7.56435	1.161519	2.83462	0.028958	0.23166	0.003218	0.01287
2.506435	2.055985	7.56435	1.161519	2.83462	0.028958	0.23166	0.003218	0.01287
0	0	0.003218	0	0	0	0	0	0
0	0	0.003218	0	0	0	0	0	0
2.120335	7.65444	2.606178	3.487774	1.299871	2.61583	1.698842	2.892535	6.270914
0	0	0	0.955598	0	0	0	0	0
0	0	0	0.955598	0	0	0	0	0
0	0	0	0.955598	0	0	0	0	0
0	0	0	0	0	0	0	0	0
0	0	0	0	0	0	0	0	0
0	0	0	0	0	0	0	0	0
0	0	0	0	0	0	0	0	0
0	0	0	0	0	0	0	0	0
0	0	0	0	0	0	0	0	0
1.679537	7.38417	2.197555	2.483913	1.019949	2.52574	1.509009	2.844273	6.071429
0.723938	0.817246	1.611969	1.953024	0.994208	1.557272	0.666023	2.313385	0.045045
0.723938	0.817246	1.611969	1.953024	0.994208	1.557272	0.666023	2.313385	0.045045

P086	P087	P088	P089	P090	P092	P093	P094	P095
0.01287	0	0	0	0	0.009653	0	0	0
0	0	0	0	0	0	0	0	0
25.2574	0.022523	52.59009	0.028958	49.95495	35.54698	0.02574	23.37516	62.90541
0	0	0	0	0	0	0	0	0.003218
0	0	0	0	0	0	0	0	0.003218
0	0	0.006435	0	5.357143	4.137709	0.006435	0.048263	2.207207
0	0	0.006435	0	5.357143	4.137709	0.006435	0.048263	2.207207
25.2574	0.022523	52.52574	0.009653	43.57786	31.40605	0.019305	23.32368	60.07722
25.2574	0.022523	52.52574	0.009653	43.57786	31.40605	0.019305	23.32368	60.07722
0	0	0	0	0.19305	0	0	0	0
0	0	0	0	0.19305	0	0	0	0
0	1.965894	0	0	0	0	0.009653	0.09009	0
0.003218	0.028958	0	0	0	0.003218	0	0	0
0.003218	0.028958	0	0	0	0.003218	0	0	0
0.135135	0.492278	0.138353	0.862291	0.42471	0.202703	0.067568	0.186615	0.2574
0.135135	0.492278	0.138353	0.862291	0.42471	0.202703	0.067568	0.186615	0.2574
0.238095	4.382239	0.653153	6.763192	0.135135	0.2574	0.492278	2.175032	0.16731
0.238095	4.382239	0.653153	6.763192	0.135135	0.2574	0.492278	2.175032	0.16731
0.019305	0	0.083655	0.06435	0.022523	0.032175	0.057915	0.009653	0.06435
0.019305	0	0.083655	0.06435	0.022523	0.032175	0.057915	0.009653	0.06435
0.086873	3.034106	0.003218	0.579151	0.33462	0.006435	0.061133	0.279923	0.01287
0.086873	3.034106	0.003218	0.579151	0.33462	0.006435	0.061133	0.279923	0.01287
14.1184	10.26384	15.29923	31.84041	21.63449	15.95238	12.23295	10.9009	8.918919
7.815315	7.79601	7.004505	22.90541	16.33205	8.825611	7.93758	10.17053	7.483912
7.815315	7.79601	7.004505	22.90541	16.33205	8.825611	7.93758	10.17053	7.483912
1.048906	0	0.112613	0.003218	0.19305	0.144788	0.913771	0	0.009653
1.048906	0	0.112613	0.003218	0.19305	0.144788	0.913771	0	0.009653
1.679537	1.386744	1.721364	2.74453	1.029601	0.920206	0.370013	0.23166	0.318533
1.679537	1.386744	1.721364	2.74453	1.029601	0.920206	0.370013	0.23166	0.318533
0	0	0	0	0	0	0	0	0
0	0	0	0	0	0	0	0	0
3.593951	2.261905	1.364221	4.765122	3.185328	3.918919	3.610039	4.259974	3.471686
0	0	0	0	0	0	0	0	0
0	0	0	0	0	0	0	0	0
0	0	0	0	0	0	0	0	0
0	0	0	0	0	0	0	0	0
0	0	0	0	0	0	0	0	0
0	0	0	0	0	0	0	0	0
0	0	0	0	0	0	0	0	0
0	0	0	0	0.02574	0	0	0	0
0	0	0	0	0.02574	0	0	0	0
3.259331	2.258687	1.287001	4.424067	2.921493	3.745174	3.439511	4.253539	3.471686
2.213642	1.003861	1.010296	2.631918	1.685972	3.484556	2.207207	0.907336	2.187902
2.213642	1.003861	1.010296	2.631918	1.685972	3.484556	2.207207	0.907336	2.187902

P096	P097	P099	P100	P101	P102	P103	P104
0	0	0	0	0	0	0	0
0	0	0	0	0	0	0	0
15.14157	40.87838	0.016088	0.07722	2.80888	73.53604	0.849421	0.009653
0	0.003218	0	0	0	0	0	0
0	0.003218	0	0	0	0	0	0
0	0	0	0.003218	0.05148	7.612613	0.054698	0.003218
0	0	0	0.003218	0.05148	7.612613	0.054698	0.003218
15.14157	40.86873	0.01287	0.074003	0.646718	65.85264	0.791506	0.006435
15.14157	40.86873	0.01287	0.074003	0.646718	65.85264	0.791506	0.006435
0	0	0	0	0	0	0	0
0	0	0	0	0	0	0	0
0	0	0	0	0	0	0	0.572716
0	0	0	0	0	0	0	0
0	0	0	0	0	0	0	0
0.250965	0.186615	0	0.086873	0.305663	0.086873	0.563063	0.643501
0.250965	0.186615	0	0.086873	0.305663	0.086873	0.563063	0.643501
0.199485	0.871943	0.453668	0.485843	0.209138	0.21879	0.42471	1.103604
0.199485	0.871943	0.453668	0.485843	0.209138	0.21879	0.42471	1.103604
0.009653	0.032175	0.02574	0.03861	0	0.01287	0.006435	0
0.009653	0.032175	0.02574	0.03861	0	0.01287	0.006435	0
0.176963	0.019305	0.022523	0.009653	2.123552	0.106178	0.267053	0.24453
0.176963	0.019305	0.022523	0.009653	2.123552	0.106178	0.267053	0.24453
4.694337	16.21943	26.75032	9.475547	14.99678	8.729086	3.516731	3.902831
2.47426	9.810167	13.34299	4.942085	11.86293	7.232947	2.413127	2.454955
2.47426	9.810167	13.34299	4.942085	11.86293	7.232947	2.413127	2.454955
0	0.003218	0.209138	0.003218	0.016088	0.048263	0	0
0	0.003218	0.209138	0.003218	0.016088	0.048263	0	0
0.186615	1.036036	1.467181	0.238095	0.559846	0.357143	0.16731	0.016088
0.186615	1.036036	1.467181	0.238095	0.559846	0.357143	0.16731	0.016088
0	0	0	0	0.016088	0	0	0
0	0	0	0	0.016088	0	0	0
1.438224	2.149292	1.290219	4.629987	3.285071	1.110039	3.803089	5.392535
0	0.003218	0	0	0	0	0	0
0	0.003218	0	0	0	0	0	0
0	0.003218	0	0	0	0	0	0
0	0	0	0	0	0	0	0
0	0	0	0	0	0	0	0
0	0	0	0	0	0	0	0
0	0	0	0	0	0	0	0
0	0	0	0	0	0	0	0
0	0	0	0	0	0	0	0
1.396396	1.840412	1.077864	0.292793	2.715573	1.029601	3.474904	5.01287
0.846203	1.373874	0.781853	0.286358	1.113256	0.701416	1.595882	0.003218
0.846203	1.373874	0.781853	0.286358	1.113256	0.701416	1.595882	0.003218

Supplementary Table 8 Distinguished bacterial genus between pairs of healthy and PCOS

Genus	PCOS-HB	PCOS-LB	p value	Fold change
Alistipes	0.010559847	0.014968095	0.014316469	1.417453796
Butyricimonas	0.001181469	0.002325989	0.036720705	1.968726202
Enterorhabdus	0.000900259	0.000849959	0.002441721	0.944126895
Erysipelotrichaceae_UCG.003	0.00617632	0.002914522	0.03034363	0.471886457
Eubacterium._nodatum_group	7.65771E-05	0.000114629	0.032125587	1.496913969
Eubacterium._ventriosum_group	0.000866797	0.00166305	0.019937985	1.91861456
Family_XIII_UCG.001	0.000209141	0.000322422	0.040423451	1.541648501
Holdemania	5.27775E-05	0.000115305	0.020117381	2.184742157
Lachnoclostridium	0.025337194	0.015814028	0.014495952	0.624142833
Oscillibacter	0.000715574	0.001650986	0.041959567	2.307219368
Ruminococcaceae_UCG.002	0.002538613	0.003822798	0.02381716	1.505861187
Sellimonas	9.00966E-05	0.000341192	0.004388034	3.786963713
Terrisporobacter	0.000299234	0.00076617	0.037028739	2.560435356
Turicibacter	0.000143505	0.000496035	0.017002465	3.456569517
Tyzzerella	0.000351353	0.000195732	0.026499969	0.55708242
un_f_Christensenellaceae	3.2178E-05	2.81609E-05	0.037571621	0.875160669
un_f_Clostridiales_vadinBB60_group	0.000166669	0.000229253	0.033102623	1.375503549
un_f_Flavobacteriaceae	1.02985E-05	9.38464E-05	0.039548591	9.112631694

Genus	Healthy	PCOS-LB	p value	Fold change
Alistipes	0.010559847	0.014968095	0.014891748	1.417453796
Alloprevotella	0.00039318	0.000567757	0.042784735	1.444010974
Catenibacterium	0.000377736	0.000229918	0.016826599	0.608673005
Dorea	0.00237838	0.002504294	0.006829676	1.052940716
Gemella	0.00002446	3.35208E-05	0.028063874	1.370434723
Granulicatella	0.000123561	6.30234E-05	0.002811083	0.510061119
Lachnospiraceae_FCS020_group	0.000410558	0.000523514	0.023129464	1.275126816
Lachnospiraceae_UCG.008	9.07415E-05	0.000130054	0.0475425	1.43323287
Lactococcus	2.50995E-05	7.10542E-05	0.042518198	2.830902176
Mailhella	0.000005148	0	0.010400226	0
Megamonas	0.025711716	0.016605671	0.020880066	0.645840634
Prevotella_9	0.086876453	0.058508287	0.000519224	0.673465413
Romboutsia	0.004613901	0.014152993	0.003880624	3.06746811
Ruminococcus._gauvreauii_group	0.000915704	0.001059768	0.016468778	1.157325733
Slackia	8.3675E-06	2.48011E-05	0.007703576	2.963975202
un_f_Muribaculaceae	0.00680567	0.011575645	0.040895907	1.700882417

Genus	Healthy	PCOS-HB	p value	Fold Change
Dorea	0.003719603	0.00237838	0.026683996	0.639417805
Enterorhabdus	0.00027349	0.000900259	0.010117397	3.291746972
Escherichia.Shigella	0.0042742	0.023231663	0.006024062	5.435324715
Eubacterium._ventriosum_group	0.001557949	0.000866797	0.000716431	0.556370623
Faecalibacterium	0.110499221	0.085180823	0.049182509	0.770872612
Family_XIII_AD3011_group	0.00064943	0.000319822	0.047773449	0.492465741
Fournierella	0.000114306	0	0.04590338	0
Fusicatenibacter	0.012491533	0.008484559	0.041479089	0.679224805
Granulicatella	2.2023E-05	0.000123561	0.003806576	5.610516839
Holdemania	8.72158E-05	5.27775E-05	0.016965696	0.605136986
Lachnoclostridium	0.016980797	0.025337194	0.016735821	1.492108709
Lachnospiraceae_FCS020_group	0.000837398	0.000410558	0.009270827	0.490278596
Lachnospiraceae_NC2004_group	0.000319213	8.23701E-05	0.031509937	0.258040758
Lachnospiraceae_UCG.008	0.000180355	9.07415E-05	0.00422163	0.503128231
Lactobacillus	3.38711E-05	0.002063066	0.014230467	60.90941614
Mailhella	0.00021083	0.000005148	0.039128867	0.024417722
Marvinbryantia	0.000157499	0.000064999	0.023135122	0.412695373
Prevotella_9	0.120546303	0.086876453	0.002361622	0.720689486
Ruminococcaceae_UCG.002	0.005613696	0.002538613	0.002242934	0.452217637
Ruminococcaceae_UCG.004	0.001207413	0.000448521	0.003131237	0.371472653
Ruminococcaceae_UCG.014	0.007713543	0.002390607	0.008201	0.30992338
Ruminococcus._gauvreauii_group	0.002712018	0.000915704	0.019068052	0.337646709
Ruminococcus._gnavus_group	0.006745751	0.018780569	0.040609608	2.784058806
Slackia	0.000150718	8.3675E-06	0.001524613	0.055517638
Subdoligranulum	0.023987333	0.012081082	0.008264837	0.503644231
un_f_un_o_Rhodospirillales	0.001419089	0	0.008946566	0

Supplementary Table 9 The NSTI values in each sample

sample	NSTI Value	sample	NSTI Value	sample	NSTI Value
N1	0.058412968	P009	0.0863645	P059	0.085514
N2	0.119318714	P010	0.0495405	P060	0.088162
N3	0.102016305	P012	0.0531846	P061	0.06775
N4	0.078835495	P013	0.0750214	P062	0.086563
N5	0.057595868	P014	0.0777947	P063	0.06988
N6	0.051654325	P015	0.0829671	P064	0.091297
N7	0.081876984	P016	0.0769928	P065	0.063104
N8	0.11612009	P017	0.1005227	P066	0.063333
N10	0.080948006	P018	0.0550401	P067	0.059427
N11	0.104409482	P019	0.1233564	P068	0.065123
N12	0.06420647	P020	0.0570762	P069	0.08347
N14	0.076460454	P021	0.0949879	P070	0.078079
N15	0.113021002	P022	0.0767652	P071	0.078727
N16	0.083497851	P023	0.0941961	P072	0.065415
N17	0.078524469	P024	0.0704757	P073	0.052177
N18	0.080045483	P025	0.0781439	P074	0.067124
N19	0.090981758	P026	0.0374375	P075	0.078285
N20	0.073084951	P027	0.0497517	P076	0.076578
N21	0.070314408	P028	0.0771082	P077	0.079505
N22	0.094734067	P030	0.0551904	P078	0.078677
N23	0.059364002	P031	0.0907671	P079	0.109084
N24	0.05287757	P033	0.165063	P080	0.099099
N26	0.098681019	P034	0.2237109	P081	0.071556
N27	0.062277964	P035	0.0781268	P082	0.063969
N28	0.062313133	P036	0.0555136	P083	0.087311
N29	0.096321335	P037	0.0788318	P084	0.097478
N30	0.057479974	P038	0.0725223	P085	0.052127
N31	0.081979752	P039	0.0634507	P086	0.074996
N32	0.069778625	P040	0.0707827	P087	0.061534
N33	0.069729591	P041	0.0994683	P088	0.098686
N34	0.08015555	P042	0.0653631	P089	0.074242
N35	0.059600968	P043	0.1055916	P090	0.093539
N36	0.063750218	P044	0.0831195	P092	0.089554
N37	0.083652224	P045	0.0625346	P093	0.063875
N38	0.09325404	P046	0.0404138	P094	0.062211
N39	0.099332087	P047	0.0595481	P095	0.085801
N40	0.092003997	P048	0.0608834	P096	0.06095
N41	0.083363487	P049	0.0813149	P097	0.085612
P001	0.075599609	P050	0.0402791	P099	0.089855
P002	0.051573806	P051	0.1154136	P100	0.050518
P003	0.073272317	P052	0.0719939	P101	0.06246
P004	0.065459978	P053	0.0543146	P102	0.100693
P005	0.055174594	P054	0.1804858	P103	0.057169
P006	0.106227175	P056	0.0738778	P104	0.046099
P007	0.081019285	P057	0.0579342		
P008	0.076251454	P058	0.0749007		

Supplement Table 10 Distinguished metabolites between healthy and PCOS-HB

sample	N10	N11	N12	N16	N18
Group	Healthy	Healthy	Healthy	Healthy	Healthy
PC (20:4/22:6)	1571222.823	431169.7411	107274.7217	102932.2616	878515.2631
D-Sphingosine	157107185.2	94601395.81	85656184.29	162663601.6	96562675.54
PC (18:3e/10:0)	563054.2014	521737.0243	775216.9426	731007.8796	1136229.282
2-[1-(4-isobutylphenyl)ethyl]-5-(3-nitrophenyl)-1,3,4-oxadiazole	7222751.363	3477844.914	4987251.653	5142830.121	1870244.57
1-(4-benzylpiperazino)-2-(pyridin-2-ylamino)propan-1-one	385714.3436	438069.4614	196391.0373	193130.181	243268.8934
Caffeine	3469713.281	3045413569	125209891.2	465515219.5	4627239.434
LPC 17:2	7522688.146	3034049.449	7285392.484	4829449.456	6501767.352
all-cis-4,7,10,13,16-Docosapentaenoic acid	10878619.41	10213167.7	7484861.367	7289931.52	13957121.04
1,7-bis(4-hydroxyphenyl)heptan-3-one	473330.8135	483665.5301	398961.7755	420384.0557	402192.308
N,N'-di[4-(2,6-dimethylmorpholino)phenyl]thiourea	1331210.87	310028.5328	2169413.212	1674489.453	2773249.806
Lysopc 15:0	24855668.49	6706526.82	10074304.12	21328516.72	19845023.27
5-(tert-butyl)-2-methyl-N-(5-methyl-3-isoxazolyl)-3-furamide	750937.428	630115.9534	1664402.83	1193387.598	433254.9597
PC (14:1e/8:0)	5450370.556	2767473.297	16671587.23	1182268.529	6192358.539
11-α-Hydroxy-17-methyltestosterone	1678291.844	8703548.004	1370092.954	1069031.357	2640200.646
PC (22:6e/19:1)	829225.4984	532828.0568	513319.5885	506496.8121	468624.2752
Noroxycodone-d3	2272969.911	2246496.656	1322968.675	1271562.493	1806376.115
Thiamine	322605.4029	289347.5357	398897.8969	369557.1184	384278.7243
Mesterolone	7797133.607	15975696.89	8107387.199	4911645.187	13419164.17
Argininosuccinic acid	109503.4199	238727.2685	233853.7494	139300.5815	280441.042
Irganox 259	3241697.897	815848.6239	10352707.1	6543773.577	13745280.12
3-(3,4,5-trimethoxyphenyl)propanoic acid	688404411.3	1112900099	1656390252	137031457.4	377431730.3
4-methoxy-6-(prop-2-en-1-yl)-2H-1,3-benzodioxole	17146230.65	13832238.11	23911265.2	4603440.412	15259869.29
Acetylcarnitine	2779396.521	2542821.054	1540974.821	1638611.251	2847342.274
Agnuside	820941.9788	93389.85899	104261.3556	112958.0891	223488.9406
LPC 20:5	25689784.42	26307161.77	47154959.27	7439831.367	3376159.559
Estrone sulfate	396844.0695	416290.6623	164943.0548	677269.6573	279903.429
5-Methoxyindole-3-Carbaldehyde	3656537.741	41708859.13	6231373.666	1828224.245	3601288.263
4-(octyloxy)benzoic acid	1718908.272	1338970.069	1177577.416	1230225.833	1549140.319
LPC 22:1	994971.5795	758306.436	4864686.541	475062.8745	2067237.013
Guanidinosuccinic acid	1765622.551	1919252.866	5559889.42	1510991.011	2630847.559
L-Cystine	4521834.912	17571130	7106986.15	9105452.425	10111891.04
Glu-Gln	7623795.744	4409908.149	8354233.032	10013557.75	8755911.928
LPC 18:2	202690983.7	89684044.01	97231732.01	113027226.6	141603649.3
Glycoursodeoxycholic acid	9657252.61	3513724.792	10666253.07	16287754.39	20388725.1
ethyl 2-cyano-3-(tetrahydro-3-thiophenylamino)acrylate	5748109.449	7556518.917	5620638.424	12360882.98	15540099.69
OxPC (16:0-20:3+1O)	10598134.23	32433509.2	23906911.64	30189049.92	24465532.64
Lysopc 18:1	94959201.36	52230275.17	54967301.04	49655148.28	82778518.46
Bilirubin	479760.3575	13967073.78	64707475.81	55377587.11	391875.9812

sample	P003	P004	P40	P012	P024
Group	PCOS-HB	PCOS-HB	PCOS-HB	PCOS-HB	PCOS-HB
PC (20:4/22:6)	181889.726	95821.2821	170847.2234	1270936.401	2316624.738
D-Sphingosine	229425362.2	352185997	246389076.5	249183281.2	216558051.7
PC (18:3e/10:0)	556679.1607	663273.6047	1525458.472	4446694.177	8783130.325
2-[1-(4-isobutylphenyl)ethyl]-5-(3-nitrophenyl)-1,3,4-oxadiazole	990954.7242	246780.7654	1776623.021	2062375.555	2442538.787
1-(4-benzylpiperazino)-2-(pyridin-2-ylamino)propan-1-one	158575.5664	257062.1221	458677.6184	946413.6721	1217876.852
Caffeine	2426569535	49669576.56	2073291649	787956698.7	4250393.941
LPC 17:2	1026398.333	3350856.612	3194727.397	3736643.487	1202489.838
all-cis-4,7,10,13,16-Docosapentaenoic acid	56329766.31	14501377.83	26921017.09	29420067.45	5467315.698
1,7-bis(4-hydroxyphenyl)heptan-3-one	414388.7978	391282.9932	365741.272	386342.4334	524737.8863
N,N'-di[4-(2,6-dimethylmorpholino)phenyl]thiourea	126126.9388	4135280.077	7429380.607	7577895.042	17203871.67
Lysopc 15:0	4811928.388	66004355.57	23530056.07	20496278.46	6994566.314
5-(tert-butyl)-2-methyl-N-(5-methyl-3-isoxazolyl)-3-furamide	471868.6505	1189099.678	489140.0877	766839.3271	521340.5652
PC (14:1e/8:0)	2606717.246	2562015.466	2111531.078	1553193.15	1824445.686
11-α-Hydroxy-17-methyltestosterone	7097202.859	7885297.151	8079292.318	6063370.423	951465.6676
PC (22:6e/19:1)	2221121.984	594599.4593	1491034.045	601691.1947	2127975.925

Noroxycodone-d3	725761.1497	1392832.035	2185195.082	5108315.269	13360399.27
Thiamine	703224.0658	885791.9379	664216.1077	511342.1663	856299.0853
Mesterolone	40513513.33	29151184.06	41085190.85	24247095.87	5827665.184
Argininosuccinic acid	90189.61213	300096.6427	637529.8546	711535.2985	1103390.187
Irganox 259	542155.9135	13826127.54	27076044.99	22107611.1	30969978.76
3-(3,4,5-trimethoxyphenyl)propanoic acid	53697437.63	495732671.3	366346326.5	314124145.6	207421750.6
4-methoxy-6-(prop-2-en-1-yl)-2H-1,3-benzodioxole	2579664.886	7332460.589	6703447.52	9246114.931	6883159.458
Acetylcarnitine	9143789.309	10982654.54	9871153.787	5937195.229	3053500.483
Agnuside	86059.98881	76961.0263	91294.5828	75659.91794	102674.1114
LPC 20:5	3537034.17	14327735.44	14132135.84	22363221.66	3325442.898
Estrone sulfate	1669590.501	1064493.166	718636.1116	493246.9848	802297.0542
5-Methoxyindole-3-Carbaldehyde	2808624.034	4889602.756	4403795.503	2151393.917	2919634.025
4-(octyloxy)benzoic acid	1000749.162	3647466.177	8819845.705	5231121.425	3483623.077
LPC 22:1	460486.5024	418343.2203	580680.9551	484938.7655	578788.2791
Guanidinosuccinic acid	1329699.778	420629.2758	1505223.364	464593.7531	1320290.792
L-Cystine	23168081.8	33294851.97	34917440.94	19162115.41	14117129.86
Glu-Gln	1586592.194	731737.2308	2944674.146	3651044.691	6731658.318
LPC 18:2	30509088.36	36148056.77	70206563.12	51151849.4	43769856.71
Glycoursodeoxycholic acid	2863694.721	5123612.216	10827604.2	16280942.05	6180321.349
ethyl 2-cyano-3-(tetrahydro-3-thiophenylamino)acrylate	59365863.69	65823005.31	12204828.23	5728627.221	12939978.1
OxPC (16:0-20:3+1O)	17983750.62	7720348.201	18647728.15	16819243.25	23297776.43
Lysopc 18:1	17274808.15	21017767.24	35338802.34	32908880.56	19499939.65
Bilirubin	1012836.202	30770790.67	291588.0244	9674911.041	3612106.875

N20	N21	N23	N27	N28	N29	N3	N30	N32
Healthy	Healthy	Healthy	Healthy	Healthy	Healthy	Healthy	Healthy	Healthy
1854419.242	302888.3956	860715.0875	195078.3274	223625.8762	211205.0614	5198314.138	83824.69723	86192.52945
118391724.7	73558081.4	132198408.5	37737681.19	62581155.22	36278447.34	109988510.8	70426663.51	87741489.42
1178471.446	1048677.226	2206177.494	1455270.236	818034.4285	1124652.904	528206.9615	999609.0521	1996457.911
6584375.645	5353462.903	6433023.793	7884722.049	23806404.41	32376413.2	4482311.405	35558577.16	5879693.854
310080.992	252859.9539	424185.6981	318951.4614	203047.9477	170758.0366	246929.2299	210707.3807	422418.3995
351686601.3	636319149.9	604633679.4	773137485.3	3447960.37	24499510.79	2767803.073	57735817.87	618218728.5
11819544.48	6380532.426	3392892.556	4765858.413	3304236.153	20397597.91	4225482.663	2243205.819	5482320.75
4083954.822	3201367.922	18212653.42	5900775.492	4365267.18	3584750.097	8532605.029	5061681.76	6374878.085
380262.7563	436874.6019	401534.2366	358784.7654	390966.22	401965.3365	376818.6446	352602.24	358134.2968
3333124.533	2291129.207	2736239.195	2937980.364	3367623.909	2503545.448	2950721.135	2570256.418	4048353.324
20876819.79	11135385.94	10917321.46	8171974.888	11252920.28	9595121.22	15094190.51	16124977.68	15058470.33
481629.2379	578169.6359	448931.4951	470926.3102	461103.4382	460235.7715	1263159.561	434127.9781	453820.4475
2620932.947	3289553.564	2705775.94	2098114.265	2315934.408	24588032.26	3692223.071	2032307.549	1661835.424
1140944.488	921999.4449	4156894.402	1278153.608	973148.2743	895982.8055	1278321.706	864738.1118	882993.8456
528338.7705	511875.4908	512105.9387	1072036.544	505830.6695	554451.0633	1050334.137	574196.9923	696573.3351
1987246.523	1688731.995	2258697.857	2376463.977	1638581.82	1303793.116	927880.275	1682149.362	2686136.354
174693.0216	172406.2481	361429.8923	432502.8622	463276.2368	357622.3801	188318.5838	976714.8168	456388.3786
1483755.595	4502226.588	24536950.77	7140198.51	5378859.378	4489653.596	18906354.4	5424740.224	5220079.702
281576.4006	355459.0916	453990.5926	328406.4963	288608.7453	223118.4114	256949.1498	299763.5827	370238.6105
14697218.07	6671044.943	12938820.57	10817826.94	18115277.59	9750057.233	12422015.4	14429431.46	18658904.53
910273670.5	753767901.8	171798830.4	1816615242	221798521.5	785104268.2	82598018.63	71205337.65	706111971.8
29343907.56	20161500.68	4214201.673	37694533.83	5021311.687	25611678.76	4961042.473	2775015.265	29421506.32
2566852.941	2583867.288	2812122.051	1709525.48	1813049.286	959057.0441	2666966.676	1820118.274	2464450.464
1522014.081	859814.6622	162435.2111	103223.4171	1825658.812	422212.1641	89234.29782	1882813.455	116233.0557
19614272	30450103.94	9025458.839	12035199.33	15314310.92	73064275.89	3496690.525	3626820.405	15538013.28
416770.3668	620208.3669	353068.8704	334148.3633	465587.868	316852.5511	92220.76263	409021.0382	359808.5254
3233639.905	3104771.699	6931914.488	11862176.23	4796561.8	4094071.49	2111025.14	3661278.823	4952367.36
1572325.95	1084685.534	1559204.63	975365.8176	1591737.513	1281807.997	918036.7251	1287202.709	1357621.28
362129.0948	709197.5142	698214.3635	565792.9309	621518.9937	7760486.221	991074.5283	647058.9177	727328.2514
2040839.461	3230398.738	1924129.774	4105925.603	2475994.2	1281566.184	711022.1618	611295.0855	2694278.538
9472920.382	19243189.71	12732587.28	6080160.898	5742897.25	1274578.743	11863880.85	1160614.067	4988575.367
4579243.176	8999145.431	4923623.629	8012896.969	14496269.8	15180088.24	7936569.954	8547288.304	9610906.865
82102149.25	85790059.48	93375805.01	78716827.49	94682873	97250783.94	123409058	88068219.44	115692533.9
16172344.38	37489510.81	75872076.14	191919050.5	42117757.7	27407710.99	22039182.79	18628838.39	4592609.815
6052042.432	5521585.807	16718396.93	9372997.904	9136082.013	4112876.234	1473269.161	5045715.29	7931675.596
28403580.11	119325907.3	13458067.18	29077272.57	36477810.22	26024368.05	23716271.59	146324420.7	41130087.65
53520064	49268338.81	37082756.58	43468936.18	60222581.78	45175446.19	56350892.18	26120683.47	40008490.07
259150.1188	10652767.51	11510215.93	4990825.785	482826.9897	2066892.918	64401457.66	3259977.586	1212935.725
P026	P033	P035	P049	P058	P065	P072	P073	P074
PCOS-HB	PCOS-HB	PCOS-HB	PCOS-HB	PCOS-HB	PCOS-HB	PCOS-HB	PCOS-HB	PCOS-HB
145185.0401	163683.6786	117179.3628	149623.1595	253741.1941	319204.9428	96444.04938	134070.2743	143417.583
199541988.3	201571441.6	313208218.1	270588058.4	154932890.2	404025930.4	89712659.58	154629446.8	168365033.6
778645.5658	10796185.97	7440494.656	778057.555	2458483.477	744204.3476	7587131.927	6263435.442	1854360.511
2297822.947	1593389.826	936606.4824	8661920.502	5474994.291	3463270.821	1206837.653	724103.6214	3460052.769
248859.7174	1131173.097	1040356.525	835913.0019	608703.1483	537389.9219	932742.4702	1077086.409	481238.7697
49867374.41	70464304.13	2057749.914	200123497.4	2849502434	2916214.994	596403010.3	1339748418	2335965643
1954523.03	3877262.091	3802850.806	1061080.611	2570533.995	897785.4074	1593808.734	3560269.151	5667213.131
12055994.17	11343133.03	36293252.33	9919881.156	12753626	21021906.92	6617828.54	27414957	8738129.46
425879.7995	941308.8563	5324609.271	577764.9547	433207.218	569739.9806	807012.4393	1401675.485	380026.064
3606583.017	19256654.25	14764758.89	117956.1198	4148238.516	129663.9775	11607591.64	10772350.51	5088183.575
7000327.201	8338254.482	10077635.25	10484348.18	10546795.94	52105377.73	54986454.85	40300101.82	128872128.4
560675.3992	846522.1494	3113840.525	820045.6481	543253.2747	991279.2689	412096.2502	485464.4552	507090.9497
3000856.782	2957093.938	4227119.859	2826784.751	2949374.493	2190101.446	1409772.989	2037013.958	3211950.933
998676.9388	1218200.509	26364730.72	2553456.942	1822099.419	28201540.4	1628221.346	21908278.71	1757867.553
2595473.245	1506223.975	1227129.487	4938775.479	1460977.423	666141.1433	425527.4553	566578.1038	1910699.357

1665954.743	11311417.95	10059028.63	3179799.415	4004531.332	3218806.035	14041001.1	13180333.22	2990589.306
529474.291	534587.0544	686146.0933	592316.7839	759426.195	748364.0871	851574.4814	462589.4703	750084.8829
9455371.549	12575738.4	32082702.86	18505960.13	22625594.56	30082701.01	12151703.69	35216934.28	14013786.31
561563.7948	2764543.413	1339164.554	97026.36034	758043.9925	100971.8093	1395404.088	984878.6863	555719.1622
27420929.78	76357929.06	54088994.52	668033.5008	19913316.41	2958149.81	66542962.07	61280279.79	23114689.06
242282336.3	275784409.2	130370496.7	97105422.47	420662552.8	112324791	251347481.3	563354347	583290142.5
12788496.61	10166020.14	3817131.562	5094365.221	11323155.56	3316765.326	6146614.185	19767931.66	10918849.55
3330154.102	2974588.841	13811821.81	5666171.037	5578080.875	11332564.17	4987592.312	11491933.72	4548218.036
92333.10776	111840.9012	97631.47047	81556.00385	113520.3531	92904.25523	93847.28513	83894.43605	70018.86685
6635976.116	9913861.63	35223881.7	4144250.864	6814817.381	4573632.548	5621227.509	17937538.91	17472790.93
622158.6085	934960.159	1159022.592	1178580.339	788187.7365	644352.9903	1043042.552	496053.138	522445.5742
3243262.488	3020953.524	3960607.628	2332003.926	2829841.54	2700339.124	3996347.541	13419525.21	4925072.034
14765570.83	4454564.481	6812812.147	687338.3955	18186279.83	1152336.182	5967637.646	5478126.436	611733.8676
370166.3685	495385.5919	867714.0226	333973.2007	234529.9519	360316.1902	269765.4873	431015.3548	402679.8529
683382.4265	540927.9246	462946.618	1319178.995	521196.3527	293879.9543	669849.899	1435417.242	1217855.701
17045256.61	37874561.04	56493907.71	25239977.86	25528614.23	48691777.51	34200888.19	47887104.2	17964878.74
4956716.003	7175700.51	2233209.552	3641998.722	4491853.71	2882405.469	7463640.785	2388845.214	3465068.204
43444600.25	57228969.91	36870148.41	29835919.25	28627948.5	50710537	32794916.46	39268875.39	43181793.19
14102523.29	16612471.61	6521438.685	20960654.52	8252452.352	4450214.71	26787884.52	38386912.08	24703141.69
4447604.519	26851779.59	23761148.28	13760825.62	7577093.703	13087795.49	32223663.82	28913196.18	18887458.2
13570819.34	9919243.869	13300602.45	27450482.22	12834601.58	18690165.23	13566972.81	10893432.36	12270331.14
18955248.1	24194506.88	31087997.82	16144687.7	19378803.22	28022663.53	18162988.45	24330426.37	23891818.53
7179488.254	34787342.11	187945041.8	42860112.57	5559142.084	66456988.95	849993.2893	256796.5448	311990.6806

N33	N34	N35	N36	N4	N5
Healthy	Healthy	Healthy	Healthy	Healthy	Healthy
189405.4539	105990.9443	110330.3217	2130512.183	241817.5323	5018578.501
158824124.5	135283281.9	183522289.6	170262476	118263917.7	132807090.7
1276124.145	2440136.916	1296902.778	9352878.22	705714.2165	6580858.904
5022086.597	11101419.98	17562882.97	10521623.7	5166973.28	3479862.335
234498.3503	516737.414	291660.1975	1031148.232	268006.5193	902062.345
222399132.3	771621019.8	258402479	51106177.82	95277640.84	1722806.8
15153303.8	4491800.463	2840720.631	3176121.832	12120736.37	3080732.142
9461795.558	14025728.22	4158662.946	10063479.52	14836200.85	4633566.898
397423.9819	423749.7912	410899.1902	686014.5825	439066.9053	371252.5903
2936350.866	2262872.465	3412061.302	9781356.402	4288263.454	10176630.73
7451479.702	7908165.667	6887912.189	11714526.46	9920760.573	7513765.08
1759131.052	3318562.834	6117147.53	4187271.896	7374524.307	448222.7996
1900755.212	2102998.569	2361563.803	2284087.069	19964899.88	1069369.077
1510365.93	2823497.583	839913.112	1330592.752	13706353.48	757537.3637
2081598.871	642192.2326	553499.9424	1419828.799	1554034.742	1514089.702
1778967.324	2483031.486	2112318.786	10554615.82	1967516.064	10280881.83
481888.7725	188234.2783	214128.1022	164852.0943	275113.0978	312516.8195
10931378.84	13385396.78	4679491.696	8804136.593	27780562.44	2659321.483
281484.135	307993.8959	313281.2904	970810.3269	380167.5447	992858.2067
16123419.63	10003279.85	11947538.79	39669874.65	9990317.677	25398848.43
274517032.8	699740187	576593205.2	515420466.5	371574267.4	505111135
15612797.72	12945726.73	14932054.27	10808257.42	24875237.59	12542598.78
2891280.022	2009038.687	2158158.599	2182134.77	7750784.764	2375551.009
166346.2127	165273.7564	208622.8482	2717847.625	235553.2786	113580.8891
31819289.82	14363340.02	7174489.252	8747064.498	65297192.04	17652264.96
446605.1131	131075.498	345771.2932	460485.5531	329858.5872	381800.1056
5888415.12	19621380.05	4485409.887	7604403.667	5036047.05	17219431.14
1371398.022	1177418.258	1759167.62	3429928.951	5064458.236	3381676.249
849333.708	471164.6844	563174.7382	509008.981	8292226.663	841313.1811
1781292.808	1734040.51	1959886.395	2560733.518	1263540.076	2774398.716
7063442.586	12520838.13	7333259.155	7909321.026	36508597.74	14719227.78
8609485.103	7801853.642	11713809.79	9291304.863	4106900.726	11959806.42
105645112.8	53264746.97	64416621.93	60040958.47	82824751.82	99022647.6
15413517.03	7243209.578	48382093.79	40928066.36	8230769	181932953.2
3929102.063	4907950.651	10277692.85	9995378.364	4410277.521	8097481.579
48255135.04	140650494	11503826.44	22252835.44	23212193.53	29594792.9
42708165.05	32181941.55	34255780.82	36457384.93	43986458.4	44103693.56
137550876.9	155238512.7	240019496.1	184290308.1	371318424.7	316092.6184

P077	P080	P096	P101	P103	P104
PCOS-HB	PCOS-HB	PCOS-HB	PCOS-HB	PCOS-HB	PCOS-HB
241945.7341	109174.2657	136201.3737	94966.88501	118756.9058	119522.675
220554874.1	98865552.48	313387640.9	258152912.1	139068506.5	200465389.2
651954.5448	11997692.75	7624449.38	782924.9783	968526.5342	806857.7103
2935280.947	12757510.73	4790335.006	1775969.34	4577405.177	4203774.048
1022911.096	1606165.893	1027738.515	292490.946	321177.7387	456867.0225
2013446513	2899678.775	1598669732	1697237911	168097571.7	2778772.277
1149193.1	1876831.778	2791466.494	1891475.889	2693071.09	1998040.284
16259512.03	7006636.557	23980049.88	7568503.09	7969332.838	9165546.648
538901.5147	1757002.076	2382125.339	382670.9048	344164.974	433528.3017
114268.7963	15056799.85	7482369.663	2948363.776	3317407.603	3466012.85
5343031.897	13766258.11	53892761.14	103530225.2	92678591.95	11258120.67
636131.125	475771.4192	445785.8249	482412.6147	506858.3139	565112.8891
3114002.083	2803950.496	1868516.667	2049159.988	2392975.678	1848617.527
2553204.504	1910494.652	7851704.979	1552487.844	1340935.384	1483110.336
4833881.218	2096306.752	1233387.669	1354950.548	1471347.515	1718094.889

3723618.052	13531106.32	8878667.961	1548344.249	1766308.643	1804049.41
827036.0548	882908.2519	807605.3206	920325.8138	1224037.109	335075.506
12666111.1	11459209.34	23266371.94	18067884.66	10356687.38	12435719.07
89610.6974	1896381.716	780589.464	398997.1725	645980.5241	548950.118
674424.9643	82322240.87	31404992.44	16414372.91	11448099.78	10865620
481262918.1	84859534.36	432703861.9	564004213.4	488268442.8	51550580.2
7016058.965	2912032.201	13114319.01	10374340.33	9885057.737	2092047.155
6497674.187	2890258.02	10315900.9	5991171.128	2260817.067	2735144.067
784621.8736	2366364.349	80941.01953	75875.70452	82119.62002	139671.455
14016183.39	7364493.199	7492941.643	8469628.602	4887661.235	4131650.842
480697.8992	525915.3167	653024.1813	809214.7561	904026.5633	1024674.725
4983041.487	3359579.135	2497544.933	4288435.711	3140066.954	2853623.308
1103518.726	7502593.065	3350992.068	1293149.934	1433750.312	1327121.829
437777.2622	363711.962	371430.7243	373587.9678	302666.2702	235522.9482
1151485.682	3981967.329	930899.1888	986137.1119	812848.6521	772903.1116
27264684.19	14199878.24	38388402.18	7715964.838	10850107.53	7024494.622
8465154.507	6308922.098	2895475.86	5880137.138	3332161.137	5001916
31613179.38	66744058.61	26882047.3	23658233.39	37639066.76	36594437.41
5154481.842	21367548.31	13836901.94	5688516.397	48536900.62	14555190.62
21812348.97	16837148.87	18359945.97	29999718.91	20952502.54	20788123.39
14960362.54	13435644.86	7467547.388	13207230.03	11369658.15	10639834.19
21789747.75	28401272.97	19311795.6	18600971.03	19786478.87	21944628.2
705567.2454	672366.4459	276198.8315	10795341.5	606626.7985	14153225.81

Supplement Table 11 Distinguished metabolites between healthy and PCOS-LB

sample	N10	N11	N12	N16	N18	N20	N21	N23
Group	Healthy	Healthy	Healthy	Healthy	Healthy	Healthy	Healthy	Healthy
3-Acetoxyurs-12-en-23-oic acid	4796496.061	2986405.213	4144935.88	4362319.544	4519910.04	4670121.777	5760503.744	7493626.124
PC (22:6e/19:1)	829225.4984	532828.0568	513319.589	506496.8121	468624.275	528338.7705	511875.4908	512105.9387
Estrone sulfate	396844.0695	416290.6623	164943.055	677269.6573	279903.429	416770.3668	620208.3669	353068.8704
PC (16:1/17:2)	88729.84834	83944.49213	74718.7297	131930.8435	75174.279	184061.4908	464732.0508	76225.2654
Lysopc 15:0	24855668.49	6706526.82	10074304.1	21328516.72	19845023.3	20876819.79	11135385.94	10917321.46
4-(octyloxy)benzoic acid	1718908.272	1338970.069	1177577.42	1230225.833	1549140.32	1572325.95	1084685.534	1559204.63
Agnuside	820941.9788	93389.85899	104261.356	112958.0891	223488.941	1522014.081	859814.6622	162435.2111
4-Chlorophenol	298058.9076	166709.3044	253785.155	283725.4933	224640.781	258416.9134	237470.4537	347843.0464
2-[1-(4-isobutylphenyl)ethyl]-5-(3-nitrophenyl)-1,3,4-oxadiazole	7222751.363	3477844.914	4987251.65	5142830.121	1870244.57	6584375.645	5353462.903	6433023.793
2-Amino-1,3,4-octadecanetriol	22220249.21	4293119.806	3094838.83	5455628.774	4354035.78	39225120.11	26255317.22	4220782.519
Hesperetin	225928.2241	554378.495	223890.822	214195.4681	211405.938	212026.5989	222558.6918	202300.9594
LPC 22:1	994971.5795	758306.436	4864686.54	475062.8745	2067237.01	362129.0948	709197.5142	698214.3635
D-(+)-Maltose	2350282.174	719503.8699	1263431.86	1917530.945	1752447.68	2361446.298	7150545.794	2007046.605
SM (d21:1/21:0)	92488.37611	77466.74649	72458.1407	71081.37789	73255.2803	584877.5192	742991.921	746369.5973
(2E)-4-Hydroxy-3,7-dimethyl-2,6-octadien-1-yl beta-D-glucopyranoside	66244.8717	98055.64854	939859.778	65075.72836	60449.9146	45374.11204	88160.65512	48049.5132

sample	P001	P014	P022	P031	P032	P043	P034	P037
Group	PCOS-LB	PCOS-LB	PCOS-LB	PCOS-LB	PCOS-LB	PCOS-LB	PCOS-LB	PCOS-LB
3-Acetoxyurs-12-en-23-oic acid	9413576.217	8098844.515	7701523.29	13582924.65	13251303.3	15002270.17	11566707.79	12975653.26
PC (22:6e/19:1)	1394797.924	1085971.542	1698408.77	2894732.811	1822557.23	2113994.264	1767440.41	2608819.879
Estrone sulfate	893132.2457	873684.3048	1296523.98	1735516.727	865450.762	488293.2813	1223837.045	856885.2563
PC (16:1/17:2)	268400.0928	370121.1847	381966.572	177153.3653	766584.188	346096.8279	414346.5004	376308.3996
Lysopc 15:0	73285567.13	8428302.389	42811449.1	32441542.56	1832940.67	47703939.56	44414347.71	49761934.28
4-(octyloxy)benzoic acid	1016539.463	6283147.922	8953798.22	11007201.4	1020103.22	17029537	22261399.32	4539515.655
Agnuside	136797.3694	88343.77713	108548.033	94036.50431	94439.1573	101992.0709	115886.8798	117485.5182
4-Chlorophenol	2281310.033	1329382.871	743997.245	264556.0725	1295869.72	316143.5169	721166.2205	5015729.588
2-[1-(4-isobutylphenyl)ethyl]-5-(3-nitrophenyl)-1,3,4-oxadiazole	3391214.991	2456189.812	1402982.27	5987656.484	9078307.14	2026559.477	3699776.692	2639120.169
2-Amino-1,3,4-octadecanetriol	2844833.265	2320610.102	2814513.94	2932122.964	2663903.31	2276617.525	4248340.445	2708126.824
Hesperetin	199265.2116	270528.8407	328888.495	340828.9884	1174542.62	1494893.265	2439334.574	340380.986
LPC 22:1	486641.2055	281630.92	996642.137	620314.2703	427074.403	823542.3529	563889.8887	468419.6535
D-(+)-Maltose	1731930.967	1542884.235	1258733.39	785139.7905	1220326.57	416566.9029	1141208.866	947841.6809
SM (d21:1/21:0)	1414477.275	1266167.252	879471.784	1617902.848	919988.477	1559328.074	1391369.67	2090464.304
(2E)-4-Hydroxy-3,7-dimethyl-2,6-octadien-1-yl beta-D-glucopyranoside	52653.47302	50787.95004	144595.053	55687.26338	143058.079	59056.48619	80765.41049	65429.87709

N27	N28	N29	N3	N30	N32	N33	N34	N35	N36	N4	N5
Healthy	Healthy	Healthy	Healthy	Healthy	Healthy	Healthy	Healthy	Healthy	Healthy	Healthy	Healthy
4909183.676	7605574.636	5864579.657	5252623.743	5911201.972	4723029.067	10280568.13	7140716.72	5881738.47	416455.782	8394210.95	7283181.35
1072036.544	505830.6695	554451.0633	1050334.137	574196.9923	696573.3351	2081598.871	642192.2326	553499.942	1419828.8	1554034.74	1514089.7
334148.3633	465587.868	316852.5511	92220.76263	409021.0382	359808.5254	446605.1131	131075.498	345771.293	460485.553	329858.587	381800.106
293400.8217	86266.38333	122891.7581	84667.62859	247959.9602	76753.8167	350934.5845	281647.973	200919.153	505302.678	194329.895	260853.089
8171974.888	11252920.28	9595121.22	15094190.51	16124977.68	15058470.33	7451479.702	7908165.667	6887912.19	11714526.5	9920760.57	7513765.08
975365.8176	1591737.513	1281807.997	918036.7251	1287202.709	1357621.28	1371398.022	1177418.258	1759167.62	3429928.95	5064458.24	3381676.25
103223.4171	1825658.812	422212.1641	89234.29782	1882813.455	116233.0557	166346.2127	165273.7564	208622.848	2717847.63	235553.279	113580.889
322043.7978	286377.4041	146534.6209	345585.4559	137878.0618	253794.0347	247792.518	225518.1882	236125.443	316886.21	392332.924	249336.391
7884722.049	23806404.41	32376413.2	4482311.405	35558577.16	5879693.854	5022086.597	11101419.98	17562883	10521623.7	5166973.28	3479862.34
5596573.394	65897243.26	29743115.58	5366035.811	104210819.8	5012890.509	4674439.172	5889464.481	5644006.23	210432370	6136561.24	4067527.22
214655.8174	199422.5364	214769.3644	198494.683	214781.8693	207312.9781	231029.437	221816.5414	208141.369	280442.494	403609.754	265102.775
565792.9309	621518.9937	7760486.221	991074.5283	647058.9177	727328.2514	849333.708	471164.6844	563174.738	509008.981	8292226.66	841313.181
2095856.063	1885051.428	10526584.42	1564778.171	15172734.86	1829494.363	1065775.829	1525115.766	1056803.3	1371158.76	1049293.66	1666017.33
858266.2232	1384225.023	1035282.465	983621.2668	986550.5698	720192.7605	1179853.633	1266002.015	1279151.63	903410.511	986030.655	1498498.68
136967.5032	65017.21954	1829015.003	54580.7065	37010.73812	75743.92148	107037.0078	57153.42183	52714.4478	96427.8205	1910589.58	146153.496
P047	P051	P053	P057	P061	P062	P085	P084	P086	P089	P094	P092
PCOS-LB	PCOS-LB	PCOS-LB	PCOS-LB	PCOS-LB	PCOS-LB	PCOS-LB	PCOS-LB	PCOS-LB	PCOS-LB	PCOS-LB	PCOS-LB
13053138.77	13714573.83	13749370.14	15975735.44	11379907.31	10671048.67	14936716.05	11821097.46	19133997.2	11259346	11213320.7	8395483.6
1765079.684	3386382.285	5058565.286	2237671.576	2900087.518	1579863.27	1332394.164	2725918.33	1807115.24	1948549.76	2011996.61	2828821.75
643656.7998	823453.4658	743625.9967	985964.825	773901.7068	696216.4377	978295.6314	568596.1459	636826.119	1093347.1	1638706.72	263798.706
377964.3863	696258.4162	733510.5074	591831.6557	452650.124	465209.7896	250464.6338	451603.7013	229101.01	451874.031	896192.699	378858.03
50552166.89	11046642.45	17656143.79	7737220.271	13379402.08	10926875.71	74523317.33	68330717.4	9837908.48	59962659.4	62880937.4	5719701.66
7740184.231	5826726.311	817798.9525	17575091.35	889852.6252	1149500.928	3018868.208	4494179.368	5278396.78	1107728.56	1649592.56	1905931.37
76658.92892	158191.885	128244.0308	80416.92339	90426.90862	110521.1916	107346.4255	142808.232	98282.8913	72109.3725	89150.1122	149718.451
246419.0751	8946636.772	370225.3271	255718.7634	780853.3772	1570002.846	6558154.638	551949.2361	339929.246	150507.402	268354.165	951739.065
1738069.903	8542727.036	3507106.198	1105144.167	3382906.093	2206282.019	2749590.113	5146361.704	5806307.5	7184429.12	10173423.8	7244176.04
2077432.917	4772381.988	4563006.859	2747500.98	3345523.547	2656441.81	6724470.513	3888988.322	2838099.02	4323546.71	3708947.41	4484366.64
9061317.763	389476.3233	298555.616	253055.459	5252247.931	392183.1912	267781.2241	237649.4129	2971007.56	219545.661	232275.478	202867.808
383529.5872	604947.554	710192.5154	445820.1439	1259885.379	680940.5576	530707.7454	751344.075	527294.996	464462.383	457344.471	269028.114
939068.4791	1215996.567	1453593.13	1447967.588	2132622.099	2690092.415	1888430.285	1000946.579	1686982.07	1283912.84	2476982.03	1389097.54
1823521.982	19881780.9	1453218.111	952190.7142	1170331.82	2629425.773	2334797.274	1496054.495	1442676.46	1429881.8	1647107.2	853223.124
44410.98223	48532.92757	65334.75722	49298.13292	121684.1233	62897.47937	132159.238	65338.65316	60305.2271	57649.4267	87115.4774	65221.5014
