# Supplementary material for: Reduced stress-associated FKBP5 DNA methylation together with gut microbiota dysbiosis is linked with the progression of obese PCOS patients
Source: NPJ Biofilms Microbiomes. 2021 Jul 15;7:60. doi: 10.1038/s41522-021-00231-6 (PMC8282850; doi:10.1038/s41522-021-00231-6)
Supplement: Supplementary file 2 — Reporting Summary [file 41522_2021_231_MOESM2_ESM.pdf]

nature research

Corresponding author(s): Guoshu Yin

Last updated by author(s): Jun 20, 2021

Reporting Summary

Nature Research wishes to improve the reproducibility of the work that we publish. This form provides structure for consistency and transparency in reporting. For further information on Nature Research policies, see our Editorial Policies and the Editorial Policy Checklist.

Statistics

For all statistical analyses, confirm that the following items are present in the figure legend, table legend, main text, or Methods section.

n/a	Confirmed	
☐	☒	The exact sample size (*n*) for each experimental group/condition, given as a discrete number and unit of measurement
☐	☒	A statement on whether measurements were taken from distinct samples or whether the same sample was measured repeatedly
☐	☒	The statistical test(s) used AND whether they are one- or two-sided<br>*Only common tests should be described solely by name; describe more complex techniques in the Methods section.*
☐	☒	A description of all covariates tested
☐	☒	A description of any assumptions or corrections, such as tests of normality and adjustment for multiple comparisons
☐	☒	A full description of the statistical parameters including central tendency (e.g. means) or other basic estimates (e.g. regression coefficient) AND variation (e.g. standard deviation) or associated estimates of uncertainty (e.g. confidence intervals)
☐	☒	For null hypothesis testing, the test statistic (e.g. *F*, *t*, *r*) with confidence intervals, effect sizes, degrees of freedom and *P* value noted<br>*Give P values as exact values whenever suitable.*
☒	☐	For Bayesian analysis, information on the choice of priors and Markov chain Monte Carlo settings
☒	☐	For hierarchical and complex designs, identification of the appropriate level for tests and full reporting of outcomes
☒	☐	Estimates of effect sizes (e.g. Cohen's *d*, Pearson's *r*), indicating how they were calculated

Our web collection on statistics for biologists contains articles on many of the points above.

Software and code

Policy information about availability of computer code

Data collection	The clinical data were collected with WPS (2019).
Data analysis	Bacterial diversity was determined by sampling-based OTU analysis and is presented by Shannon, Simpson, Chao1, ACE, observed species, and Pielou’s evenness (J) index, which was calculated using the R program package ‘vegan’ (version 2.5.6). Bray-Curtis distance-based β-diversity metrics were obtained with vegdist and PERMANOVA with the Adonis function, and analysis of similarity (ANOSIM) was conducted to compare the bacterial differences among different sample subgroups. The shared OTUs were calculated and visualized using the R package VennDiagram (version 1.6.20). The significantly distinguished taxa and predicted pathways by PICRUSt were screened by comparison between the PCOS and healthy groups by the Wilcoxon test. LEfSe analysis was performed to identify taxa with differentiating abundance in the different groups. Pearson’s correlation between the abundances of differential genus taxa and pathways was computed by the R package stats (version 3.6.0), and the package pheatmap (version 1.0.12) was used to conduct the correlation heatmap. The network graphs were made using Cytoscape.

For manuscripts utilizing custom algorithms or software that are central to the research but not yet described in published literature, software must be made available to editors and reviewers. We strongly encourage code deposition in a community repository (e.g. GitHub). See the Nature Research guidelines for submitting code & software for further information.

Data

Policy information about availability of data

All manuscripts must include a data availability statement. This statement should provide the following information, where applicable:

- Accession codes, unique identifiers, or web links for publicly available datasets
- A list of figures that have associated raw data
- A description of any restrictions on data availability

The dataset supporting the conclusions of this article is available in the NCBI Sequence Read Archive repository under the accession number SUB8691740 (https://submit.ncbi.nlm.nih.gov/subs/sra/SUB8691740/attributes). Code and scripts used in the analyses are available upon request.

Field-specific reporting

Please select the one below that is the best fit for your research. If you are not sure, read the appropriate sections before making your selection.

☒ Life sciences ☐ Behavioural & social sciences ☐ Ecological, evolutionary & environmental sciences

For a reference copy of the document with all sections, see nature.com/documents/nr-reporting-summary-flat.pdf

Life sciences study design

All studies must disclose on these points even when the disclosure is negative.

Sample size	Ninety-eight PCOS patients with a normal BMI (PCOS-LB, BMI<24), 50 PCOS patients with high BMI (PCOS-HB, BMI≥24) and 38 healthy individuals with a normal BMI were recruited from the First Affiliated Hospital Shantou University Medical College Hospital between September 2018 and July 2020.
Data exclusions	No exclusions.
Replication	The DNA derived from all samples were remained for further analysis if necessary.
Randomization	This study is not randomization as we recruited healthy and PCOS patients based on clinical diagnose.
Blinding	This study is not blinding as we recruited healthy and PCOS patients based on clinical diagnose.

Reporting for specific materials, systems and methods

We require information from authors about some types of materials, experimental systems and methods used in many studies. Here, indicate whether each material, system or method listed is relevant to your study. If you are not sure if a list item applies to your research, read the appropriate section before selecting a response.

Materials & experimental systems

n/a	Involved in the study	
☒	☐	Antibodies
☒	☐	Eukaryotic cell lines
☒	☐	Palaeontology and archaeology
☒	☐	Animals and other organisms
☐	☒	Human research participants
☐	☒	Clinical data
☒	☐	Dual use research of concern

Methods

n/a	Involved in the study	
☒	☐	ChIP-seq
☒	☐	Flow cytometry
☒	☐	MRI-based neuroimaging

Human research participants

Policy information about studies involving human research participants

Population characteristics	The healthy volunteers who had regular menstrual cycles and normal ovarian morphology were from the general community. Women with PCOS were diagnosed according to the 2003 Rotterdam criteria, which require the presence of at least two of the following: (1) oligo-ovulation and/or anovulation; (2) clinical and/or biochemical signs of hyperandrogenism; and (3) ultrasound findings of polycystic ovaries in 1 or 2 ovaries, ≥12 follicles measuring 2 to 9 mm in diameter, and/or ovarian volume ≥10 mL.
Recruitment	Women with PCOS were diagnosed according to the 2003 Rotterdam criteria.The study and all experimental procedures were approved by the Ethics Committee of the First Affiliated Hospital Shantou University Medical College according to the Council for International Organizations of Medical Science (ChiCTR2000041108). All participants were recruited from the

	Department of Endocrinology at the First Affiliated Hospital Shantou University Medical College between June 2019 and September 2020.
Ethics oversight	The study was approved by the First Affiliated Hospital Shantou University Medical College.

Note that full information on the approval of the study protocol must also be provided in the manuscript.

Clinical data

Policy information about clinical studies

All manuscripts should comply with the ICMJE guidelines for publication of clinical research and a completed CONSORT checklist must be included with all submissions.

Clinical trial registration	International Organizations of Medical Science (ChiCTR2000041108)
Study protocol	http://www.chictr.org.cn/showproj.aspx?proj=61008
Data collection	The study and all experimental procedures were approved by the Ethics Committee of the First Affiliated Hospital Shantou University Medical College according to the Council for International Organizations of Medical Science (ChiCTR2000041108). All participants were recruited from the Department of Endocrinology at the First Affiliated Hospital Shantou University Medical College between June 2019 and September 2020.
Outcomes	PCOS patients with PCOS had higher concentrations of total testosterone, androstenedione, dehydroepiandrosterone sulfate, luteinizing hormone and insulin resistance (HOMA-IR), but FKBP5 DNA methylation was higher in healthy controls.
